# Phosphoproteome-derived peptide libraries for deep specificity profiling of phosphatases and phospholyases

**DOI:** 10.1073/pnas.2523183123

**Published:** 2026-06-12

**Authors:** Katarzyna Radziwon, Laura A. Campbell, Lauren E. Mazurkiewicz, Sopo Jalalishvili, Izabelle Eppinger, Aanika Parikh, Amy M. Weeks

**Affiliations:** ^a^ Department of Biochemistry, University of Wisconsin–Madison, Madison, WI 53706; ^b^ Department of Chemistry, University of Wisconsin–Madison, Madison, WI 53706

**Keywords:** phosphoproteomics, phosphatases, chemical biology, phospholyases, phosphorylation

## Abstract

Protein phosphorylation is dynamically regulated by the opposing activities of phosphowriter enzymes (kinases) and phosphoeraser enzymes (phosphatases and phospholyases). While significant progress has been made toward defining the sequence preferences of kinases, the selectivity of phosphoerasers has not been explored at scale. Here, we develop an experimental platform based on tandem mass spectrometry analysis of phosphoproteome-derived peptide libraries (PhosPropels) to map phosphoeraser activity across thousands of biologically relevant phosphosites. We extract positional residue preferences to rapidly define sequence motifs recognized by eight phosphoerasers spanning diverse species of origin, protein folds, and enzymatic mechanisms, yielding biological insights into pathways targeted by these enzymes. Taking advantage of the throughput of our approach, we profiled 20 variants of the phosphothreonine lyase OspF from **Shigella* flexneri*, uncovering an intrinsic preference for p38 and Erk MAP kinase activation loops and revealing the enzyme residues that influence its selectivity for phosphothreonine. Our results establish a general method for linking phosphorylation sites to the enzymes that remove them, providing a means to dissect a key component of cellular regulatory networks.

## Significance

Protein phosphorylation regulates nearly every aspect of biology and is dynamically controlled by the opposing activities of writer and eraser enzymes. While the sequences preferences of writer enzymes have been characterized extensively, eraser specificity remains less well defined due to limitations of available tools. Here, we introduce a simple, scalable, and accessible platform for deep profiling of phosphoeraser specificity using human phosphoproteome-derived peptide libraries. Our approach enabled rapid identification of sequence motifs recognized by eight phosphoeraser enzymes spanning diverse protein folds and enzymatic mechanisms, yielding insights into the pathways targeted by these enzymes. More broadly, our results establish a general method for connecting phosphorylation sites to their erasers to shed light on a key component of cellular signaling circuits.

Protein phosphorylation is a dynamic posttranslational modification that regulates nearly every aspect of biology (1, 2). Phosphorylation signaling can be understood in terms of a well-established read/writer/eraser framework: kinases write phosphorylation marks, reader proteins (or intramolecular sensing mechanisms) interpret them to direct downstream signaling, and erasers (such as phosphatases and phospholyases) remove them to regulate signaling dynamics (3, 4). While significant progress has been made in understanding the specificity of kinases (5, 6) and reader domains (7, 8), much less is known about phosphoeraser sequence specificity. This gap stems largely from the challenges and costs of phosphopeptide synthesis and the need to obtain precise positional information.

Synthetic phosphopeptide-based approaches present challenges for the high-throughput analysis that is needed for comprehensive phosphoeraser substrate selectivity profiling (9). Array-based, bead-based, or barcoded library approaches rely on individually addressable peptides that are synthesized one by one and typically use binary readouts of phosphorylation, precluding analysis of multiply phosphorylated peptides (10–14). Pooled libraries offer greater synthetic scalability but are limited in size by the constraints of typical MS analysis workflows, requiring researchers to choose which motifs and variations to include in the library (15). Recent studies have leveraged the endogenous phosphoproteome to examine in vitro phosphatase specificity by quantifying peptide-level depletion (16) or monitoring activity on intact phosphoproteins (17). These studies demonstrate that the native phosphoproteome can serve as a valuable reservoir of biologically relevant substrates and have provided important insights into phosphatase activity. However, substrates are defined by fold-change thresholds, such that motif inference can depend on quantification depth, weaker substrates may be less readily captured, and multiply phosphorylated substrates may be difficult to deconvolute at the site level. These workflows are also less readily extended to enzymes that modify rather than remove phosphorylated sites, such as phospholyases.

These considerations motivated us to develop a complementary approach to query the intrinsic specificity of enzymes that act on phosphorylated sites. Inspired by proteomic approaches for probing the specificity of proteases and ligases (18–20), we developed an LC–MS/MS-based in vitro assay for dephosphorylation or modification of phosphopeptides derived from the human proteome. Rather than defining substrates by applying per-peptide fold-change cutoffs, we quantify enrichment and depletion of amino acid residues in positions flanking the phosphosite in enzyme-treated versus control samples. This population-level analysis enables statistical inference of enzymatic specificity without requiring precise quantification of individual peptides, and is broadly applicable to enzymes that act on phosphosites. To facilitate adoption and reproducible analysis, we provide an openly available Python package (*phospropel*) implementing the statistical methodology described here.

We applied our method to extract positional residue preferences for five phosphatases and three phosphothreonine lyases derived from phage, human, and bacteria, highlighting its utility for analyzing enzymes with diverse species of origin, protein folds, enzymatic mechanisms, and molecular functions. Leveraging the throughput of our approach, we profiled 20 variants of the phosphothreonine (pThr) lyase OspF from **Shigella* flexneri* to define the molecular basis of its substrate specificity. We find that OspF’s catalytic domain has an intrinsic preference for the pThr-Xaa-pTyr (where Xaa ≠ Pro) motif found in p38 and Erk MAP kinase (MAPK) activation loops, independent of its MAPK docking motif. We also observe that residues that do not directly contact the substrate have a strong influence on OspF’s selectivity for pThr over phosphoserine (pSer) sites. Together, our results provide significant insights into phosphoeraser specificity and establish a general method for querying enzyme specificity at proteome scale.

## Results

### Phosphoproteome-Derived Peptide Libraries (PhosPropels) Encompass Biologically Relevant Phosphoeraser Substrates.

**Fig. 1.**
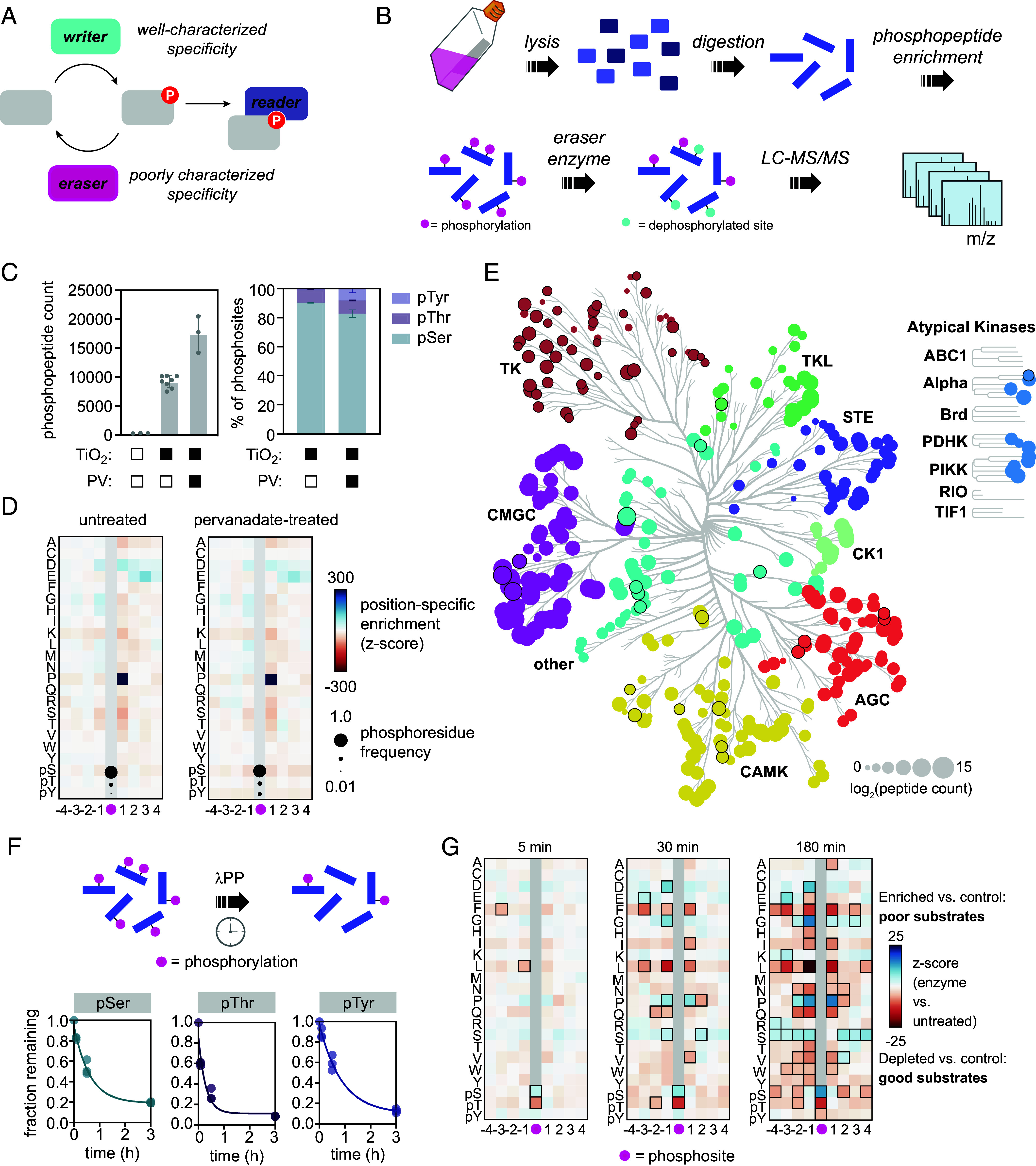
Phosphoproteome-derived peptide libraries (PhosPropels) for probing phosphoeraser specificity at scale. (*A*) The reader/writer/eraser framework for phosphorylation signaling. (*B*) Phosphopeptides enriched from biological samples provide a proteome-scale substrate library for profiling phosphoeraser specificity using LC–MS/MS. (*C*) Count and distribution of phosphosites in TiO_2_-enriched phosphopeptides from HEK293T cells in the presence or absence of pervanadate, as measured by LC–MS/MS. (*D*) LC–MS/MS analysis of phosphopeptides enables precise localization of phosphorylation modifications and enables analysis of the identity of flanking residues. (*E*) Count of phosphosites that are potential substrates of kinases across the human kinome based on kinase PSSM scoring. Node size represents the average log_2_ count of sequences that score at least 2 SD above the kinase-specific PSSM mean. Nodes with a significant (*P* < 0.05) increase in sequence count upon pervanadate treatment are outlined in black. (*F*) λ phosphatase treatment of PhosPropels leads to depletion of pSer, pThr, and pTyr sites. (*G*) Z-score heatmap showing positional enrichment or depletion of amino acid residues flanking phosphosites in λ phosphatase-treated samples compared to a no enzyme control. Position-residue combinations outlined in black were significantly (*P* < 0.0001) enriched or depleted compared to an untreated control. Gray boxes indicate insufficient representation of the residue-position combination.

To generate a diverse, biologically relevant pool of substrates for phosphoeraser profiling, we prepared phosphoproteome-derived peptide libraries (PhosPropels) from HEK293T cells by tryptic digestion followed by TiO_2_ enrichment (Fig. 1 *A* and *B*) (21). This workflow yielded 9,012 ± 767 unique phosphopeptides and 9,888 ± 989 high confidence phosphosites per replicate whose composition was consistent with previous measurements of the human phosphoproteome (90.3 ± 0.3% phosphoserine (pSer), 9.1 ± 0.1% phosphothreonine (pThr), and 0.6 ± 0.2% phosphotyrosine (pTyr) residues) (Fig. 1*C* and *SI Appendix*, Fig. S1 and Dataset S1) (22). Pretreatment of cells with pervanadate, a broad spectrum pTyr phosphatase inhibitor (23), increased representation of pTyr sites by approximately 20-fold in the treated library (1,276 ± 346 pTyr sites) compared to the untreated library (58 ± 26 pTyr sites), expanding coverage for profiling phosphoerasers that recognize pTyr residues (Fig. 1*C* and *SI Appendix*, Fig. S2 and Dataset S2). Comparable results were obtained across different cell lines, enrichment strategies, and digest proteases (*SI Appendix*, Figs. S3 and S14 and Datasets S3–S4, S17–S19, and S40). Library composition reflected protease specificity, with partial depletion of protease-recognized residues at internal positions, mitigated by protease-specific missed cleavage rates (trypsin 10 to 30%, LysC <10%, GluC >30%).

To characterize the diversity of flanking sequences in our libraries, we aligned all high-confidence phosphosites and calculated the position-specific frequency of each amino acid in positions −4 to +4 relative to the phosphosite. We then compared these frequencies to the overall amino acid frequencies in the library by calculating z-scores, enabling detection of position-specific enrichment and depletion (Fig. 1*D* and *SI Appendix*, Note 1). All 20 proteinogenic amino acids, including Lys and Arg, as well as pSer, pThr, and pTyr, were represented at every position in this window (*SI Appendix*, Figs. S8–S14). Most position-specific frequencies reflected overall amino acid abundance in the library; however, Pro at +1 was substantially enriched. Phosphosite scoring with kinase position-specific scoring matrices indicated broad representation of predicted kinase substrates across the human kinome, with increased representation of Tyr kinase substrates following pervanadate treatment (Fig. 1*E* and *SI Appendix*, Fig. S3 and Dataset S5) (5, 6). These data establish a statistical background for downstream profiling of phosphoeraser specificity. Additional characterization of PhosPropel composition and coverage is described in the *SI Appendix*, SI Text (*SI Appendix*, Figs. S15–S18).

### Statistical Profiling of Phosphoeraser Specificity Using PhosPropels.

PhosPropels enable statistical inference of enzyme specificity by comparing residue frequencies in enzyme-treated libraries to matched untreated controls. For each residue r at position i, enrichment (overrepresentation) or depletion is quantified by a z-score comparing its observed frequency in the enzyme-treated sample (f_phosphatase_) to its frequency in the untreated control (f_control_), where σ is the population SD:

$$z=\frac{f_{\mathrm{phosphatase}}-f_{\mathrm{control}}}{\sigma }.$$

Positive and negative z-scores indicate enrichment and depletion, respectively. A full derivation of this statistical framework is provided in *SI Appendix*, Note 1. We implemented these calculations in *phospropel* (https://github.com/aweeks8/phospropel), an open-source Python package that automates frequency calculation, statistical analysis, and visualization.

To benchmark PhosPropels for profiling phosphoeraser specificity, we treated them with λ phosphatase (λPP), a broad specificity enzyme that is often used for global dephosphorylation (24, 25). We quenched reactions at multiple timepoints and analyzed samples by LC–MS/MS to quantify the frequency of pSer, pThr, and pTyr among the remaining phosphorylation sites (Fig. 1*F*) and the position-specific frequency of each amino acid flanking those sites (Fig. 1*G*). λ PP had robust activity on PhosPropels, reducing phosphopeptides from 90 to 14 ± 1% of total peptides over 3 h (Fig. 1*F* and *SI Appendix*, Dataset S6). In agreement with previous studies (26), λ PP treatment resulted in progressive depletion of pSer, pThr, and pTyr sites, and phosphosites containing Pro at the +1 position were strongly disfavored as substrates (Fig. 1 *F* and *G*). We also identified previously unrecognized λ PP substrate preferences: Pro was disfavored at the −1 and −2 positions, while Phe, Leu, and Gln were preferred at the +1 and −1 positions. Similar results were obtained using PhosPropels generated with GluC and LysC (*SI Appendix*, Figs. S19 and S20 and Datasets S20 and S27). However, some protease-specific features were observed, including enrichment of Glu at −3 and Asp at −1 only in tryptic libraries, enrichment of Lys at −2 only in GluC libraries, and enrichment of Lys at −3 only in GluC and tryptic libraries. These results demonstrate that our method accurately reports on phosphatase substrate specificity, recapitulating known trends and revealing previously unappreciated ones.

### PhosPropels for Profiling the Selectivity of Human pTyr and pSer/pThr Phosphatases.

**Fig. 2.**
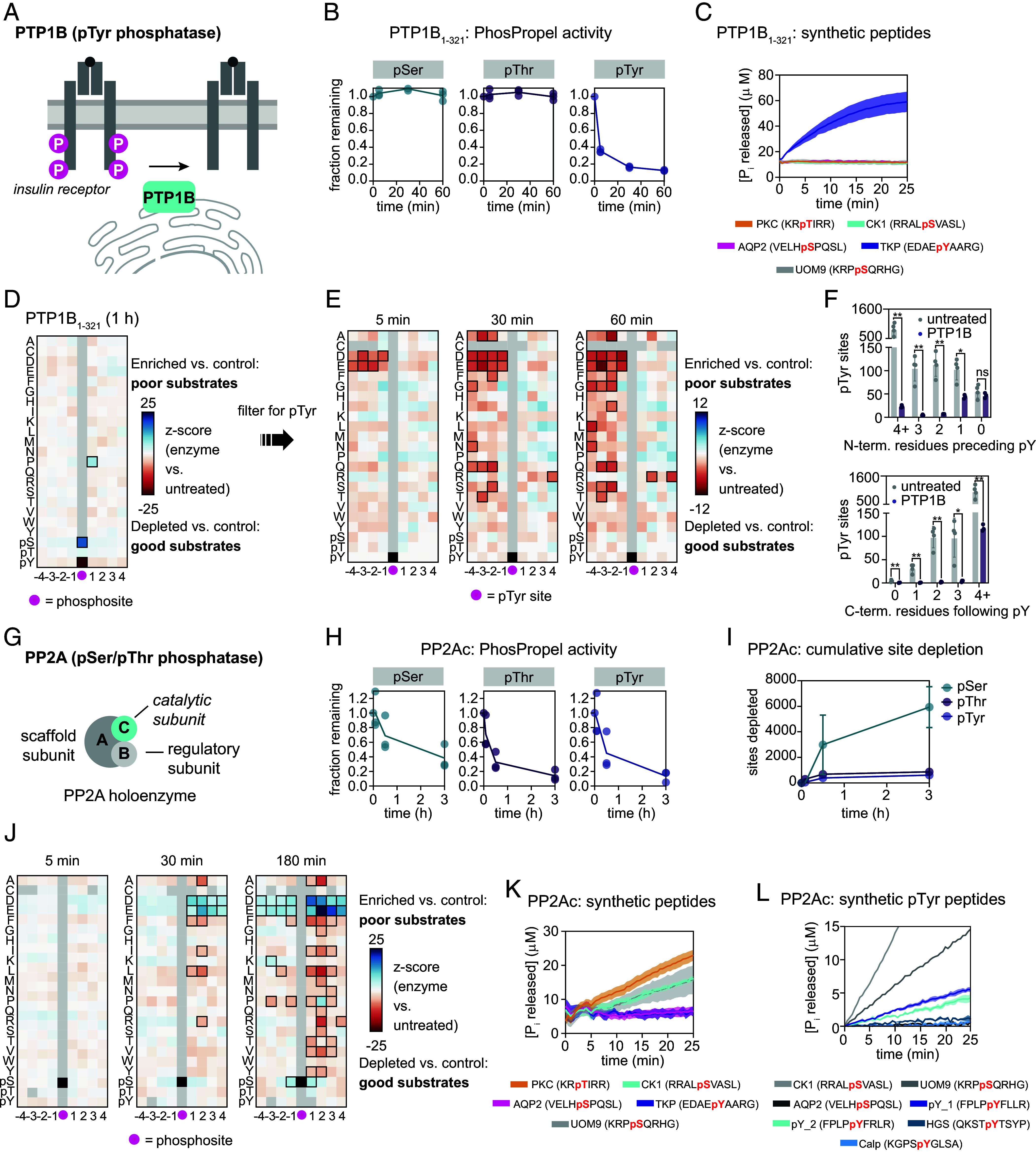
Deep profiling of human phosphatase specificity using PhosPropels. (*A*) Human PTP1B is protein tyrosine phosphatase that regulates metabolic signaling. (*B*) PTP1B_1-321_ rapidly depletes pTyr sites, but not pSer or pThr sites, from PhosPropels (n = 3 biological replicates). (*C*) PTP1B_1-321_-catalyzed phosphate release from synthetic human phosphopeptides (mean ± SD, n = 3). (*D*) Z-score heatmap comparing phosphosites in PTP1B_1-321_-treated samples versus control. (*E*) Z-score heatmap showing positional enrichment or depletion of amino acid residues flanking pTyr sites PTP1B_1-321_-treated samples compared to a no enzyme control. (*F*) Dependence of PTP1B_1 to 321_ activity on the number of residues N-terminal (*Top*) or C-terminal (*Bottom*) to pTyr. Data represent pTyr counts (n = 3 to 5 biological replicates). Unpaired *t* tests with Holm–Šídák correction were used to compare treated and untreated samples. *P < 0.05; *P* < 0.01; ns, not significant. (*G*) PP2Ac is the catalytic subunit of the pSer/pThr phosphatase PP2A. (*H*) PP2Ac depletes pSer, pThr, and pTyr sites from PhosPropels (n = 3 biological replicates). (*I*) PP2Ac-catalyzed loss of pSer, pThr, and pTyr sites from the library (mean ± SD, n = 3 biological replicates). (*J*) Z-score heatmap showing positional enrichment or depletion of amino acid residues flanking pSer sites PP2Ac-treated samples versus control. (*K*) PP2Ac-catalyzed phosphate release from synthetic human phosphopeptides (mean ± SD, n = 3). (*L*) PP2Ac-catalyzed phosphate release from synthetic pTyr peptides. (mean ± SD, n = 3). For panels (*D*, *E*, and *J*), z-scores were calculated from positional frequencies relative to the 0-min timepoint using counts summed across n = 3 biological replicates. Residue-position combinations with Benjamini–Hochberg FDR-adjusted *P* < 0.0001 are outlined in black. Gray boxes indicate insufficient representation.

We next profiled the catalytic domain of the human pTyr phosphatase PTP1B (residues 1 to 321), an important regulator of metabolic and oncogenic signaling pathways that is mechanistically distinct from λ PP (Fig. 2*A*) (27, 28). We treated PhosPropels (0.1 mg/mL) with PTP1B_1-321_ (20 nM), quenched the reactions at various timepoints, and analyzed the position-specific enrichment and depletion of each amino acid surrounding the remaining phosphosites. PTP1B_1-321_ rapidly depleted pTyr, but not pSer or pThr, from the library, consistent with both its well-established pTyr specificity and measurements of phosphate release from individual synthetic peptides (Fig. 2 *B*–*D* and *SI Appendix*, Figs. S21–S23 and Datasets S7, S22, and S29). We therefore focused our analysis on features flanking pTyr sites.

Consistent with previous studies, our data revealed strong sequence preferences on the N-terminal side of the pTyr site (Fig. 2*E*) (13, 29). Within 5 min, pTyr sites with acidic residues in the −1 to −4 positions were substantially depleted from the library, a preference that was more robustly detected in tryptic libraries compared to GluC libraries (*SI Appendix*, Fig. S24). After 30 min, pTyr sites with Gln at positions −2 to −4 were also depleted. At the 60 min timepoint, the majority of pTyr sites (88 ± 1%) were depleted. Most (72 ± 3%) pTyr sites remaining after 60 min had the pTyr residue within two amino acids of the N terminus, highlighting the importance of N-terminal amino acids in substrate recognition (Fig. 2*F*). This feature was uniquely identifiable using our method, which incorporates phosphopeptides of variable length.

In contrast, C-terminal sequence preferences were weaker. Only pTyr sites with Arg at the +2 or +4 positions were significantly depleted among the pTyr sites remaining after 60 min (Fig. 2*E*), suggesting that PTP1B tolerates a broad array of C-terminal residues. Many of PTP1B’s best studied substrates, including insulin receptor, contain a tandem pTyr motif (pYpY) (30). While pYpY sites with pTyr at the +1 position were rapidly depleted from the library upon PTP1B treatment, depletion was not statistically significant (p > 0.05), likely due to the relatively small number of pYpY sites in the library. However, we note that the pYpY site in insulin receptor (^1158^pYETDpYpYRKGG^1167^) is flanked by other features we found to be favorable (Fig. 2*E*), including acidic residues on the N-terminal side and Arg at the +2 position.

We next profiled the catalytic subunit of PP2A (PP2Ac), a pSer/pThr phosphatase involved in apoptosis, metabolism, and cell migration (31, 32). In cells, PP2Ac assembles with a scaffold A subunit and regulatory B subunits that direct its substrate selectivity (Fig. 2*G*). A complete understanding of how PP2Ac’s activity is shaped by these protein partners requires detailed knowledge of its intrinsic specificity prior to maturation and holoenzyme formation. To gain this understanding, we profiled PP2Ac using PhosPropels. Over 3 h, PP2Ac depleted pSer, pThr, and pTyr from the library (Fig. 2*H* and Datasets S8, S21, and S28). Analysis of cumulative phosphosite loss versus time revealed that despite strong fractional depletion of pThr and pTyr, total dephosphorylation events were driven by PP2Ac’s pSer phosphatase activity (Fig. 2*I*), consistent with PP2Ac’s function as a pSer/pThr phosphatase with basal pTyr phosphatase activity (33). Because the strong fractional depletion of pTyr from the library was somewhat unexpected, we sought to exclude the possibility that this activity arose from a contaminating tyrosine phosphatase in the commercial PP2Ac preparation. We therefore tested whether pTyr dephosphorylation was sensitive to okadaic acid, a selective inhibitor of PPP-type phosphatases (*SI Appendix*, Fig. S25 and Dataset S36) (34). We found that okadaic acid inhibited the depletion of pSer, pThr, and pTyr sites, consistent with the conclusion that pTyr depletion is catalyzed by PP2Ac. In contrast, no inhibition of PTP1B or two bacterial PPP-type phosphatases reported to be insensitive to okadaic acid was observed in our assay (*SI Appendix*, Fig. S25 and Datasets S37–S39).

Analysis of flanking residues preferred by PP2Ac broadly agreed with previous specificity studies using synthetic pSer/pThr peptides (Fig. 2*J* and *SI Appendix*, Figs. S26–S28) (15). Among pSer sites (Fig. 2*J*), those with Pro at the +2 position or with acidic residues on the C-terminal side were poor substrates. The latter trend was apparent only in trypsin and LysC libraries (*SI Appendix*, Figs. S28 and S29). pSer sites with aromatic or hydrophobic residues at +1 or +2 or with Arg or Ala at +2 were preferred. Phosphothreonine sites showed similar trends, although fewer residue-position combinations were as significantly enriched or depleted compared to pSer sites (*SI Appendix*, Fig. S26). In contrast, little selectivity for flanking sequences was observed for pTyr sites.

Measurement of phosphate release from individual peptides confirmed that PP2Ac displays a range of activities across substrates (Fig. 2*K*). While some pSer/pThr substrates (PKC, CK1, and UOM9, net charge +2 to +4) were dephosphorylated efficiently, others showed little activity (AQP2, net charge −1). Although we observed measurable dephosphorylation on pTyr substrates (Fig. 2*L*), their rates were substantially lower than those of the most efficient pSer/pThr substrates, consistent with basal pTyr activity (32). These results demonstrate that PP2Ac can discriminate among diverse biological substrates based on its active site properties, even in the absence of regulatory subunits, and lay the groundwork for future studies exploring how holoenzyme formation shapes substrate selectivity.

### PhosPropels Reveal Unique Substrate Profiles of Related Bacterial Effector Phosphatases.

**Fig. 3.**
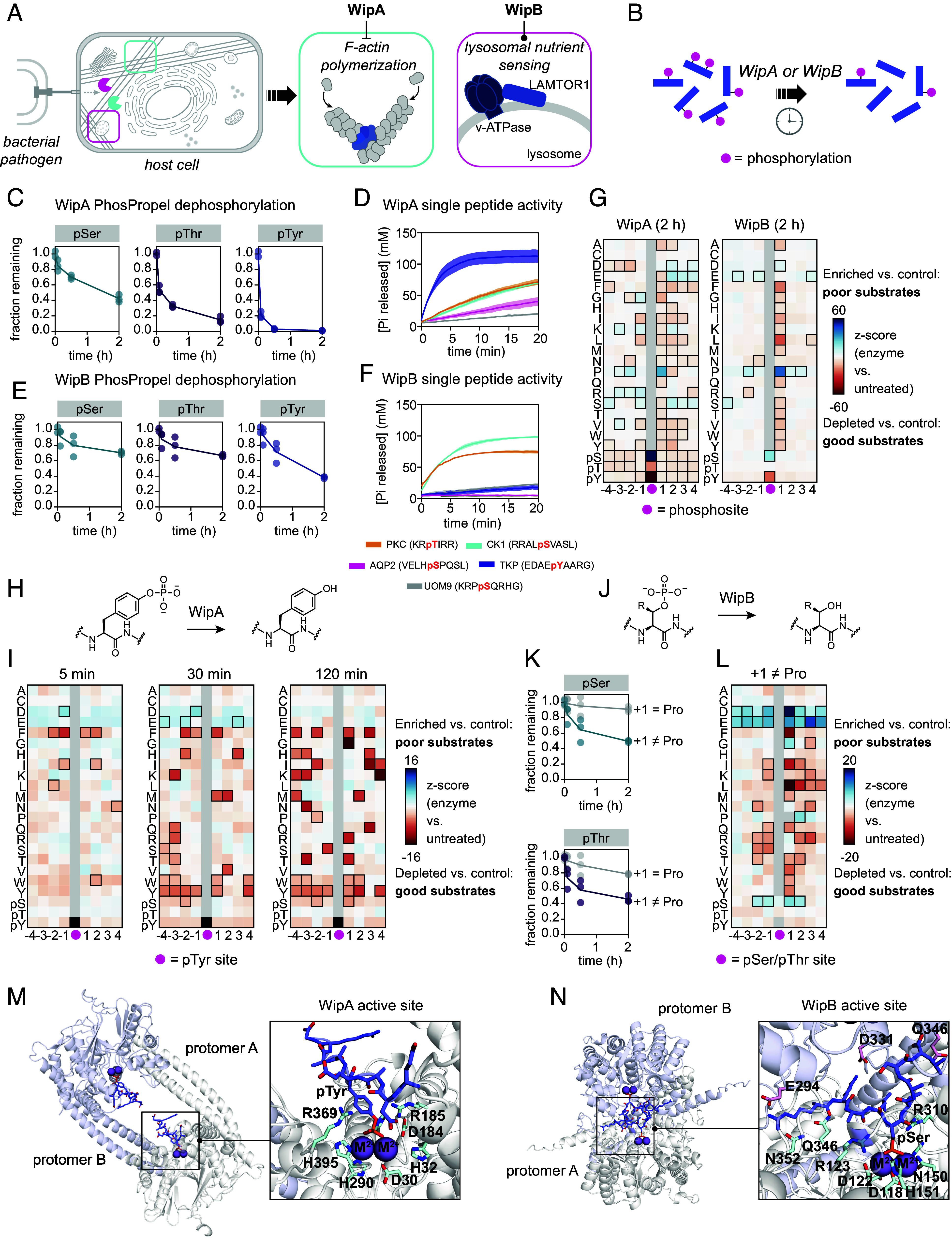
PhosPropels reveal unique substrate profiles of bacterial effector phosphatases WipA and WipB. (*A*) WipA and WipB are phosphatase effectors from **Legionella pneumophila*.* (*B*) WipA and WipB specificity were profiled using PhosPropels. (*C*) WipA depletes pSer, pThr, and pTyr from PhosPropels at different rates (n = 3 biological replicates). (*D*) WipA activity on synthetic human phosphopeptides (mean ± SD, n = 3). (*E*) WipB depletes pSer, pThr, and pTyr from PhosPropels at different rates (n = 3 biological replicates). (*F*) WipB activity on synthetic human phosphopeptides (mean ± SD, n = 3). (*G*) Z-score heatmaps comparing phosphosite features in WipA-treated (*Left*) or WipB-treated (*Right*) samples versus control. (*H*) WipA is annotated as a pTyr phosphatase. (*I*) Z-score heatmaps of pTyr sites following WipA treatment (0 to 120 min). (*J*) WipB is annotated as a pSer/pThr phosphatase. (*K*) WipB-catalyzed pSer/pThr depletion for +1 = Pro and +1 ≠ Pro. (*L*) Z-score heatmaps of pSer/pThr sites where +1 ≠ Pro following WipB treatment (0 to 120 min). (*M*) Alphafold3 model of the WipA dimer bound to the peptide EDAEpYAARG. (*N*) Alphafold3 model of the WipB dimer bound to the peptide RRALpSVASL. For panels (*G*, *I*, and *L*), z-scores were calculated from positional frequencies relative to the 0 min timepoint using counts summed across n = 3 biological replicates. Residue-position combinations with Benjamini–Hochberg FDR-adjusted *P*-values < 0.0001 are outlined in black. Gray boxes indicate insufficient representation.

Bacterial pathogens have evolved effector enzymes that modulate host phosphorylation signaling to promote infection (Fig. 3*A*) (35, 36). WipA and WipB are two phosphatase effectors from *Legionella pneumophila* whose specificity profiles remain incompletely characterized (37–39). Both enzymes contain phosphoprotein phosphatase (PPP) family phosphatase domains, but WipA has been classified as a pTyr phosphatase that inhibits F-actin polymerization and WipB has been classified as a pSer/pThr phosphatase that targets proteins involved in lysosomal nutrient sensing. These assignments were based on limited in vitro assays on individual peptides. To better define their specificities, we profiled their activity on PhosPropels (Fig. 3*B*).

We treated PhosPropels (0.1 mg/mL) from pervanadate-treated HEK293T cells with WipA (100 nM) or WipB (100 nM) (Fig. 3 *C*–*G* and Datasets S9, S10, S23, S24, S30, and S31 and *SI Appendix*, Figs. S30–S37). WipA rapidly depleted pTyr sites from the library (t_1/2_ = 2.2 ± 0.2 min), with slower depletion of pSer sites (t_1/2_ = 100 ± 13 min) and pThr sites (t_1/2_ = 19 ± 6 min) (Fig. 3 *C* and *G* and Dataset S9). This broader activity contrasts with previous studies reporting that WipA has no activity on pSer and pThr. We validated our results by measuring WipA-catalyzed phosphate release from a panel of synthetic phosphopeptides (Fig. 3*D*) and observed similarly broad activity. Given WipA’s relationship to PPP family enzymes, which typically target pSer and pThr, this broad activity is not unexpected (40). Nonetheless, WipA’s faster kinetics toward pTyr are consistent with its proposed physiological function as a pTyr phosphatase (Fig. 3 *D* and *H*).

We next examined sequence features associated with WipA activity on pTyr sites (Fig. 3 *H* and *I*). At early timepoints, pTyr sites with Phe in the −1, −2, +1, and +2 positions were significantly depleted from the library, suggesting that they are good WipA substrates, while pTyr sites with acidic residues in the +1, +3, −4, and −3 positions became enriched, suggesting that they are poor substrates. However, after 2 h, <1% of pTyr sites remained, indicating that even poor substrates are eventually dephosphorylated. WipA’s broad activity raises the question of how substrate selectivity is achieved in cells. We note that WipA contains a C-terminal domain outside the phosphatase core that may direct its activity toward specific substrates or subcellular locations (37).

WipB exhibited lower overall activity, dephosphorylating 35 ± 5% of phosphosites within 2 h (Fig. 3*E* and Dataset S10). Despite its annotation as a pSer/pThr phosphatase (39), WipB also showed weaker activity toward pTyr sites. Phosphosites with Pro in the +1 position became enriched in the library and persisted strongly after 2 h (Fig. 3*G*), suggesting that substrates with +1 Pro are disfavored. Phosphate release assays on a panel of five synthetic peptide substrates supported exclusion of +1 Pro, as we were unable to detect WipB-catalyzed dephosphorylation of a pSer-Pro peptide derived from aquaporin-2 (Fig. 3*F*).

To further analyze the role of +1 Pro in WipB substrate recognition, we grouped sites based on its presence or absence (Fig. 3 *J* and *K*). Sites lacking +1 Pro were depleted from the library more rapidly, consistent with the hypothesis that these sites are preferred by WipB. Among these, pSer/pThr sites with flanking acidic residues (Asp, Glu, and pSer) persisted among the remaining phosphosites in trypsin and LysC libraries (*SI Appendix*, Figs. S36 and S37), suggesting charge-based exclusion (Fig. 3*L*). In contrast, pSer/pThr sites with aromatic (Phe, Tyr, Trp), hydrophobic (Ile, Leu, Met, Val), and polar uncharged residues at the +1, +2, and +3 positions and those with basic residues (Arg, Lys) at the +2 and +3 positions were depleted from the library. These results suggest in the absence of +1 Pro, other flanking features shape WipB specificity.

To gain insights into structural determinants of specificity, we used Alphafold3 to model dimeric WipA and WipB in complex with high-activity substrates (Fig. 3 *M* and *N*) (41). Consistent with an existing WipA crystal structure (37), the top WipA model revealed a large cavity between the two subunits formed by an α-helical hairpin insertion. The pTyr residue formed extensive contacts with the active site, while flanking residues were solvent-exposed, consistent with WipA’s broad pTyr specificity. WipB, which lacks the helical insertion, formed a tighter dimer interface in which substrate contacts spanned both protomers. Glu 294 and Asp 331 are poised to interact with the substrate, potentially explaining the unfavorability of acidic substrates.

Our results reveal insights into the substrate sequences preferred by WipA and WipB. WipA has broad activity on pTyr, pSer, and pThr residues, while WipB has more restricted activity based on exclusion of +1 Pro and flanking residue context. Many preferred WipB substrates resemble motifs recognized by MAP3K, MAP4K, and NEK/ASK kinases (5), raising the possibility that WipB may preferentially act on targets phosphorylated by these enzymes. In contrast, substrates of Pro-directed and acidophilic kinases may be resistant to WipB-catalyzed dephosphorylation. Together, our data suggest that sequence-level substrate specificity may play an important role in defining the distinct cellular functions of WipA and WipB and lay the groundwork for identifying additional physiological targets.

### PhosPropels Reveal the Sequence Preferences of pThr Lyases, a Functionally and Mechanistically Distinct Class of Phosphoerasers.

**Fig. 4.**
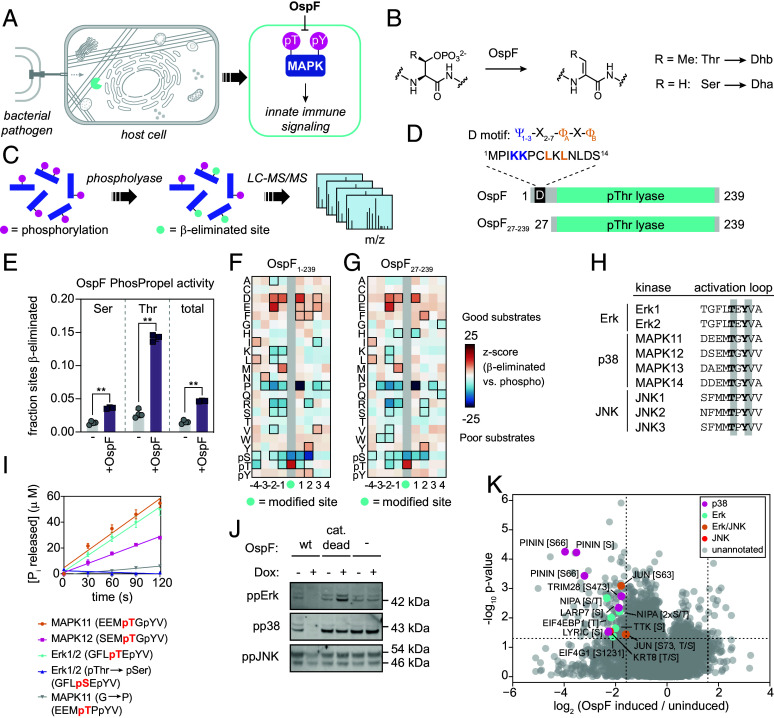
PhosPropels reveal intrinsic MAPK activation loop selectivity in OspF, a functionally distinct phosphoeraser. (*A*) OspF is a pThr lyase from **S.* flexneri*. (*B*) OspF catalyzes β-elimination of pThr and pSer, generating Dhb or Dha. (*C*) OspF activity on PhosPropels introduces Dha or Dhb in place of pSer or pThr (−18.01 Da mass shift). (*D*) Full-length OspF harbors a canonical D motif for MAPK docking. OspF_27-239_ has this motif deleted. (*E*) OspF b-elimination activity on PhosPropels. Fraction β-eliminated sites in treated (purple) vs. untreated (gray) samples were compared by the unpaired *t* test with Holm–Šídák correction. ****P < 0.0001. (*F*) Z-score heatmaps comparing β-eliminated site features in OspF-treated samples to phosphosites in the control. (*G*) Z-score heatmaps comparing β-eliminated site features in OspF_27-239_-treated samples to phosphosites in the control. For panels (*F* and *G*), z-scores were calculated by comparing positional frequencies flanking b-eliminated sites to positional frequencies flanking phosphosites with the same sample. Counts were summed across n = 3 biological replicates. Residue-position combinations with Benjamini–Hochberg FDR-adjusted *P*-values < 0.0001 are outlined in black. Gray boxes indicate insufficient representation. (*H*) MAPK activation loop sequences. (*I*) OspF activity on synthetic MAPK activation loop-derived peptides. (*J*) Immunoblots of dual-phosphorylated Erk1/2 (ppErk), p38 (pp38), and JNK (ppJNK) following doxycycline induction of OspF or OspF-K134A expression in FlpIn 293 T cells. (*K*) Label-free quantitative phosphoproteomics following OspF induction. Dotted lines mark |log^2^ fold change| = 1.585 and *P* = 0.05. Annotated MAPK substrates are color-coded (p38, magenta; Erk, cyan; Erk/JNK, orange; JNK, red).

While some bacterial effectors subvert host signaling via phosphatase activity, others target phosphosites using mechanisms not known to occur in host cells. One example is the OspF family of pThr lyases, which catalyze b-elimination of the pThr phosphate group to generate dehydrobutyrine (Dhb) (Fig. 4 *A*–*D*) (42). OspF enzymes are proposed to inactivate MAP kinases (MAPKs) by targeting their activation loop pThr-Xaa-pTyr motifs, downregulating the host immune response. Previous studies have characterized OspF specificity (43, 44) but were limited in the substrate positions and amino acid substitutions examined.

To comprehensively evaluate pThr lyase specificity, we used PhosPropels as substrate pools for OspF (Fig. 4*C*). After treating libraries (0.25 mg/mL) with OspF (5 mM for 24 h), we used LC–MS/MS to identify high-confidence b-elimination sites based on a −18.01 Da mass loss from Ser and Thr residues (ptmRS site localization probability >90%). OspF catalyzed b-elimination of both

pSer (to form dehydroalanine, Dha) and pThr (to form dehydrobutyrine, Dhb) residues (Fig. 4*E* and Datasets S11, S25, and S32 and *SI Appendix*, Figs. S38–S40). The frequency of Dhb sites relative to Dha sites was significantly higher than the frequency of pThr relative to pSer in untreated samples, indicating that pThr is the preferred substrate.

To define substrate preferences, we compared amino acid frequencies surrounding b-eliminated sites to the amino acid frequencies surrounding remaining pSer/pThr sites in each sample (Fig. 4*F* and *SI Appendix*, Supplementary Note 1). OspF disfavored Pro in the +1 position, while acidic residues and Phe were most preferred. At the +2 position, aromatic residues including Phe, Tyr, and pTyr were the most preferred amino acids. N-terminal to the pSer/pThr site, acidic residues and Met at the −1 position were preferred, while positively charged amino acids, Ser, and Pro were disfavored. Similar results were obtained when GluC and chymotrypsin PhosPropels were used, with no apparent protease-specific differences (*SI Appendix*, Figs. S39 and S40). An OspF homolog, SpvC, from *Salmonella enterica* had similar activity and specificity (*SI Appendix*, Fig. S41 and Dataset S12). In contrast, the pThr lyase HopAI from *Pseudomonas syringae* had little detectable activity on PhosPropels (*SI Appendix*, Fig. S41 and Dataset S12), in line with previous reports on its low activity (43). A sequence alignment for OspF, SpvC, and HopAI that highlights key residues is shown in *SI Appendix*, Fig. S42.

The sequences preferences identified using PhosPropels mirror the activation loop sequence motif of MAPKs (45, 46), which are the physiological substrates of OspF (42). Although MAPK targeting is proposed to depend on interactions with a canonical docking peptide at the OspF N terminus (Fig. 4*D*) (47), our experiments used short peptides, minimizing docking interactions. To test whether the catalytic domain alone is sufficient for MAPK activation loop recognition, we analyzed the specificity of an OspF variant with its docking peptide deleted (OspF_27-239_). We found that this variant retained the substrate preferences of full-length OspF (Fig. 4*G* and *SI Appendix*, Figs. S43 and S44 and Datasets S13, S26, and S33), supporting the conclusion that the catalytic domain harbors an intrinsic preference for MAPK activation loop sequences.

Among the three MAPK families in humans, the intervening residue between pThr and pTyr in the activation loop motif differs. In Erk1/2, this residue is Glu, while in the p38 MAPKs, it is Gly and in the JNK family, it is Pro (Fig. 4*h*) (48). Our data suggest that Erk and p38 kinases are good substrates for OspF, while JNK is not due to the disfavored pThr-Pro motif. To test this, we measured OspF-catalyzed phosphate release from synthetic phosphopeptides derived from p38-family kinases (MAPK11 and MAPK12), Erk1/2, and a pSer variant of the Erk1/2 activation loop. We also tested a variant of the MAPK11 sequence in which the intervening Gly residue was substituted with Pro (Fig. 4*I*). Consistent with our phosphoproteome-derived peptide library results, the MAPK11, MAPK12, and Erk1/2 peptides were processed efficiently, while little activity was detected for the Erk1/2 pThr-to-pSer peptide or the MAPK11 Gly-to-Pro peptide under the same conditions. These predictions are consistent with previous work showing that Erk1/2 and p38 are cellular OspF substrates (42, 47).

To test whether these preferences are reflected in cells, we generated HEK293T lines expressing doxycycline-inducible OspF or a catalytically inactive K134A mutant and compared MAPK activation loop phosphorylation in OspF-induced versus uninduced cells by immunoblotting. Following stimulation with EGF (Erk1/2, p38) or anisomycin (JNK), induction of wild-type OspF abolished phosphorylation of Erk1/2 and p38, whereas induction of the K134A mutant had no effect and JNK phosphorylation was unchanged (Fig. 4*J*; OspF expression and loading controls shown in *SI Appendix*, Fig. S45).

We next examined the broader impact of OspF expression on the cellular phosphoproteome using label-free or TMT-based quantitative phosphoproteomics (Fig. 4*K* and Datasets S14 and S41). In our label-free dataset, we measured 19,286 phosphopeptides, of which 163 were significantly downregulated and 15 upregulated upon OspF induction (|log_2_ fold-change| ≥ 1.585, *P* ≤ 0.05). Using the PhosphoSite Plus database and relevant literature (49, 50), we queried whether downregulated phosphopeptides were downstream of Erk, p38, or JNK MAPKs. While the majority of significantly changed phosphosites had no kinase annotation, 6 were annotated substrates of p38, 7 of Erk1/2, 2 of both Erk and JNK, and 1 of JNK (Fig. 4*K*). The sole JNK-exclusive annotated substrate corresponded to keratin, a common contaminant protein in mass spectrometry datasets. Similar trends were observed in another quantitative phosphoproteomics experiment performed under EGF stimulation (*SI Appendix*, Fig. S46 and Dataset S42). Together, our biochemical and cellular data suggest that OspF exhibits intrinsic sequences preferences that shape its impact on the host cell phosphoproteome.

### PhosPropels Enable Characterization and Engineering of OspF Specificity.

**Fig. 5.**
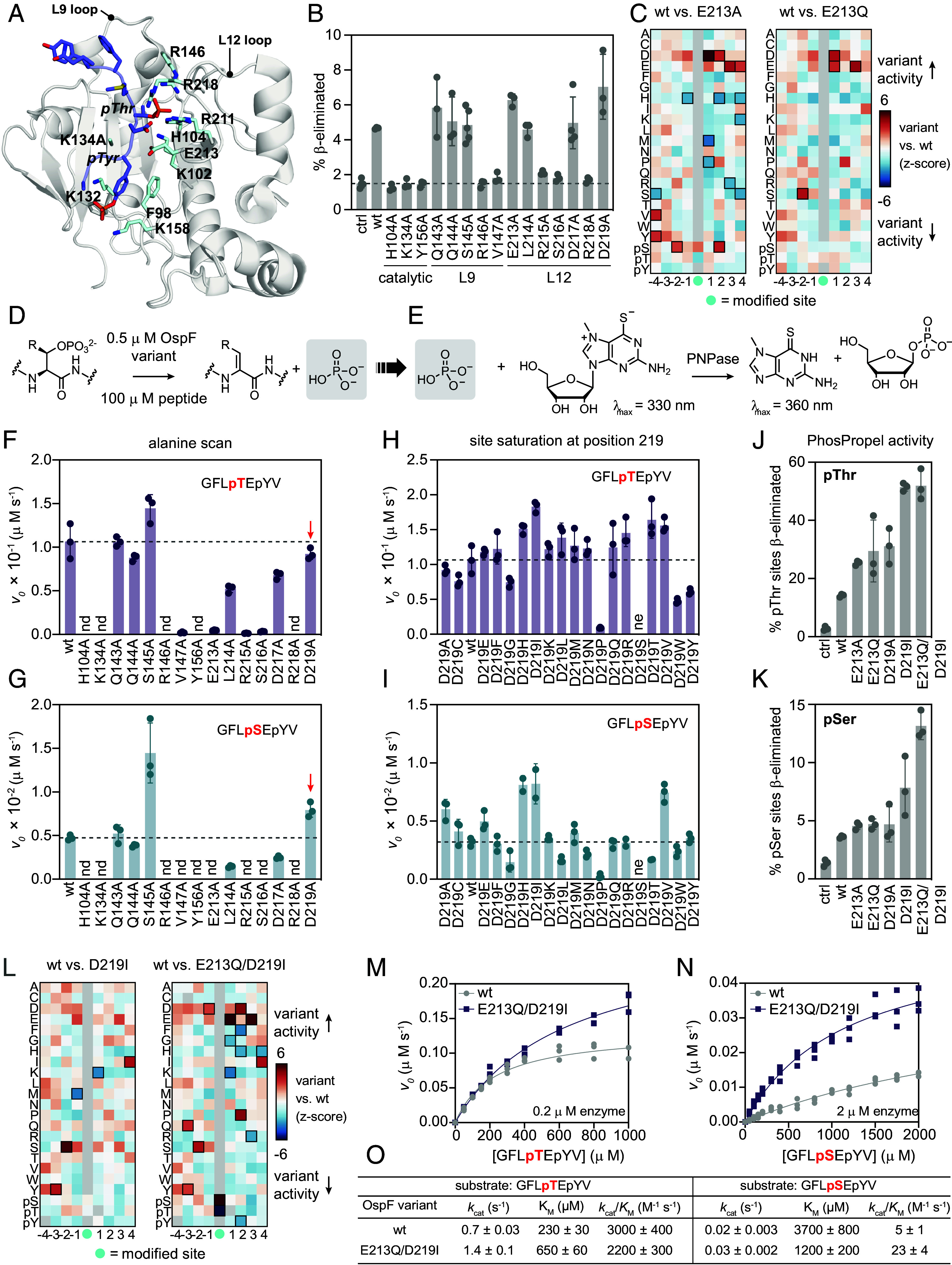
Dissecting the molecular basis of OspF sequence selectivity using PhosPropels. (*A*) Crystal structure of OspF homolog SpvC from *Salmonella enterica* bound to the peptide QYFM-pThr-E-pTyr-VA (PDB ID: 2Q8Y). Residues are numbered according to OspF numbering. (*B*) β-Elimination activity of OspF variants on PhosPropels. The dotted line indicates no-enzyme control. (*C*) Z-score heatmaps comparing β-eliminated sites generated by OspF-E213A and OspF-E213Q to wild-type. (*D*) Screen design to measure OspF activity. (*E*) Enzyme-coupled assay for phosphate release. (*F* and *G*) OspF alanine variant activity on (*F*) GFLpTEpYV and (*G*) GFLpSEpYV. (*H* and *I*) OspF-D219X activity on (*H*) GFLpTEpYV. and (*I*) GFLpSEpYV. (*J* and *K*) Activity of select OspF variants on (*J*) pSer and (*K*) pThr sites in PhosPropels. (*L*) Z-score heatmaps comparing β-eliminated sites generated by OspF-D219I and OspF-E213Q/D219I to wild-type OspF. (*M*–*O*) Steady-state kinetic analysis of OspF activity on (*M* and *O*) GFLpTEpYV and (*N* and *O*) GFLpSEpYV. For panels (*C* and *L*), z-scores were calculated by comparing positional frequencies flanking b-eliminated sites in OspF variant-treated samples and wild-type OspF-treated samples. Counts were summed across n = 3 biological replicates. Residue-position combinations with Benjamini–Hochberg FDR-adjusted *P*-values < 0.0001 are outlined in black. Gray boxes indicate insufficient representation.

To understand the molecular basis of the observed OspF selectivity profile, we examined a crystal structure of the pThr lyase SpvC from *Salmonella enterica* bound to the peptide QYFM-pThr-E-pTyr-VA (PDB ID: 2Q8Y) (Fig. 5*A*) (47). The peptide adopts an extended conformation, with the pThr phosphate group is coordinated by the residues corresponding to K102, H104, R146, R211, and R218 in OspF. The phosphate group of the pTyr residue at the +2 position interacts with K158 and K132, while the aromatic ring participates in a p-stacking interaction with F98, rationalizing OspF’s preference for +2 aromatic residues. The peptide −1 and −2 side chains are proximal to a basic pocket (R146, R211, and R218), potentially explaining the preference for acidic residues in these positions. Similarly, the +1 Glu side chain is coordinated by R215 and R218, explaining the acidic residue preference at this position.

To probe the contribution of OspF active site residues to substrate selectivity, we performed alanine scanning mutagenesis, targeting 15 OspF residues that are predicted to be in proximity to the substrate peptide (47). We then evaluated the activity and substrate selectivity of each variant using phosphoproteome-derived peptide libraries (Fig. 5*B* and *SI Appendix*, Figs. S47 and S48 and Dataset S15). Eight variants (H104A, K134A, R146A, R218A, V147A, Y156A, and S216A) had no activity above background (<3% of phosphosites b-eliminated), including three substitutions of residues that are likely directly involved in catalysis (K134A, H104A, and Y156A) (47, 51, 52).

Three additional inactive variants (R215A, S216A, and V147A) substitute residues that do not directly contact the substrate, but that reside in loops that undergo conformational changes upon substrate binding (Fig. 5 *A* and *B*) (47). R215 and S216 reside in loop L12, which undergoes a conformational change upon substrate binding to position R218 to coordinate the pThr phosphate group. S216 hydrogen bonds with R211 to stabilize the “in” conformation of the loop, while R215 hydrogen bonds with N30 in both apo and bound structures. Similarly, V147 is in loop L9, which undergoes a conformational change upon substrate binding to position R146 for coordination of the pThr phosphate. Loss of activity upon alanine substitution of these residues supports a critical role for L9 and L12 loop dynamics in OspF catalysis.

Other positions in the L9 and L12 loops were more tolerant of alanine substitution. Seven variants retained activity similar to wild-type, including Q143A, Q144A, and S145A in the L9 loop, and E213A, L214A, D217A, and D219A in the L12 loop (Fig. 5*B*). Among these residues, only E213 directly contacts the bound substrate, making hydrogen bonds with the backbone amide NH group of pTyr and the backbone carbonyl oxygen of pThr. The E213 side chain is in proximity to the +1

Glu side chain of the substrate peptide, which contains the pThr-Glu-pTyr motif characteristic of activated Erk MAPKs (45, 46, 48).

To evaluate effects on specificity, we compared amino acid frequencies at positions flanking the b-eliminated sites between each active alanine variant and wild-type OspF (Fig. 5*C* and *SI Appendix*, Fig. S47). E213A exhibited the most substantial changes in substrate specificity (Fig. 5*C*), with a stronger preference for acidic flanking residues and a modest increase in activity toward pSer substrates. Other variants retained specificity similar to wild-type OspF, with few significant changes.

We hypothesized that substituting the E213 side chain shifts specificity by decreasing negative charge in the active site to better accommodate acidic substrates. To test this, we made the more conservative E213Q variant and analyzed its activity (Fig. 5*D*). Like E213A, E213Q has a stronger preference for acidic residues in positions flanking the b-elimination site and higher overall activity. However, the shift toward increased reactivity with pSer sites was no longer apparent, suggesting that this effect is more related to the size of the side chain in position 213 rather than its charge.

To more directly probe OspF’s selectivity for pThr over pSer, we tested the activity of the alanine variants on two synthetic peptides: the Erk2 activation loop-derived peptide GFLpTEpYV from the (Fig. 5*F*) and its pSer counterpart GFLpSEpYV (Fig. 5*G*). All mutants that were inactive on phosphoproteome-derived peptide libraries were also inactive on both of these peptides, and library-active variants had some level of activity on both peptides. While most variants had similar activity to wild-type OspF on both peptides, we found that the S145 variant had somewhat higher activity on both the pThr and pSer substrates. Notably, the D219A variant exhibited higher activity than on the pSer substrate but not the pThr substrate, suggesting that residue 219 modulates pThr vs. pSer selectivity.

To analyze the role of position 219 further, we performed saturation mutagenesis at position 219 and measured activity toward the same peptide pair (Fig. 5 *H* and *I*). All variants except D219P retained some activity on one or both substrates. D219G severely impaired activity on the pSer substrate but not the pThr substrate, while D219H and D219I increased activity on both pSer and pThr substrates, with a more substantial increase observed for pSer. Other D219 variants had neutral or negative effects on activity. D219 is conserved in OspF, SpvC, and VirA, while it is substituted with Ala in HopA1, a low-activity homolog. These results indicate that residue 219 is a key determinant of OspF pThr selectivity.

We next analyzed selected position 219 variants in the context of PhosPropels. D219I increased total b-elimination by 2.5 ± 0.2-fold compared to wild-type, an effect that could be attributed to increased activity on both pThr and pSer sites (Fig. 5 *I* and *K* and Dataset S16). Analysis of flanking residue preferences revealed few significant changes compared to wild-type OspF (Fig. 5*L*), indicating that the D219I variant increases b-elimination based on its overall higher activity and increased tolerance of pSer substrates.

Dha and Dhb provide versatile reactive handles that can be used for cyclization, functionalization, and posttranslational mutagenesis. An efficient, broad specificity pThr/pSer lyase to install Dha/Dhb sites would be a valuable tool for these applications. Based on the broadened specificity profiles of the E213Q and D219I variants, we combined both substitutions and tested the activity of OspF-E213Q/D219I toward PhosPropels (Fig. 5 *J*, *K*, and *L*). The double variant b-eliminated 16 ± 1% of phosphosites in the library, a 3.5-fold increase compared to wild-type OspF (4.7 ± 0.1%). This level of b-elimination was also higher than either of the single variants (12 ± 3% for D219I and 7 ± 1% for E213Q). The increase in activity could be attributed to significant increases in b-elimination on both pSer sites and on phosphosites flanked by acidic residues (Fig. 5*L*). Kinetic analysis using GFLpTEpYV and GFLpSEpYV substrates showed that OspF-E213Q/D219I has a four-fold higher catalytic efficiency for b-elimination of the pSer substrate compared to the wild-type enzyme based on both an increase in *k*_cat_ and a decrease in *K*_M_ (Fig. 5 *M*–*O*). The E213Q/D219I variant therefore combines the broadened specificity of both single mutants, providing a useful scaffold for further engineering of Dha/Dhb-generating enzymes. These results highlight how the PhosPropel approach can be deployed for protein engineering to alter enzyme specificity.

## Discussion

Enzymatic erasers of phosphorylation play central roles in cellular signaling, and recent studies have applied both synthetic phosphopeptides and endogenous phosphoproteome samples to examine phosphatase specificity (16, 17, 53). Building on this foundation, we developed a complementary and generalizable approach for profiling phosphoeraser specificity using phosphoproteome-derived peptide libraries. Our approach enables statistical inference of intrinsic phosphoeraser specificity from population-level shifts in phosphosite sequence context between enzyme-treated samples and matched controls. An advantage of our method is its reliance on biological samples as a regenerable source of phosphopeptides that can be readily obtained using well-established enrichment protocols. These libraries reflect the diversity of biologically relevant phosphosites and do not require prior knowledge of which motifs a particular phosphoeraser might target.

The scale and complexity of phosphoproteome-derived peptide libraries enable robust statistical profiling of enzyme specificity. By comparing enzyme-treated samples to an appropriate statistical background, we systematically identified sequence features that influence substrate recognition based on their depletion or enrichment among the remaining phosphosites. This approach revealed both expected and underappreciated determinants of phosphoeraser specificity, including features that are rarely represented in synthetic phosphopeptide libraries, such as adjacent phosphosites and variable sequence lengths surrounding the phosphosite, as well as measurable low-level activities that are less readily captured in smaller-scale assays. The size and diversity of the libraries enabled us to apply targeted filters, such as restriction of the analysis to pSer, pThr, or pTyr sites or exclusion of phosphosites with Pro at the +1 position, while maintaining adequate statistical power. This analytical flexibility allows specific hypotheses to be tested without requiring changes to experimental design or construction of additional libraries.

Our approach yielded biological insights into phosphoerasers across a range of species of origin, protein folds, and enzymatic mechanisms. Using the same phosphoproteome-derived peptide libraries, we were able to characterize two classes of phosphoeraser enzymes with distinct molecular functions: phosphatases, which catalyze hydrolysis of the phosphate group to regenerate the unmodified side chain, and phospholyases, which catalyze b-elimination of the phosphate group to introduce an alkene. The throughput of our approach enabled a detailed dissection of the specificity determinants OspF, a pThr lyase. By profiling the activity and specificity of 15 alanine substitution variants of the enzyme, we identified residues that shape its substrate selectivity and catalytic efficiency. Targeted mutagenesis at key positions then allowed us to shift OspF’s activity toward pSer sites. These results demonstrate the power of phosphoproteome-derived peptide libraries to accelerate biological discovery by revealing principles of phosphoeraser specificity at proteome scale.

While our studies focused on phosphopeptide libraries derived from human cells, the same strategy can be extended readily to any organism or tissue from which phosphopeptides can be enriched. We showed that the composition of the library could be tuned by pervanadate pretreatment, a strategy that could be extended to any pharmacological or genetic modifier of phosphorylation signaling. Libraries could also be further tailored by additional enrichment steps (e.g., pTyr enrichment) or chemical modification (e.g., base treatment to generate dehydrated sites). More broadly, our experimental approach and statistical framework can be adapted to profile enzymes that erase or modify any posttranslational modification that can be enriched for analysis by LC–MS/MS, including acetylation, methylation, and glycosylation, among many others.

While the PhosPropel approach provides a generalizable framework for profiling intrinsic phosphoeraser specificity, several limitations should be considered. Because the assay is performed using peptide libraries, the resulting specificity profiles reflect sequence-context preferences in the absence of higher-order protein structure, subcellular localization, and protein–protein interactions, all of which can influence substrate selection in vivo. Statistical sensitivity depends on residue representation and sampling depth within the input library. Most natural amino acids are well represented in PhosPropels; however, residues with low natural abundance (such as Cys and Trp) may not be sampled sufficiently to enable robust inference, and residues targeted by the digest protease may be depleted at internal positions within the phosphopeptides. Although the latter limitation is partially mitigated by missed protease cleavages, performing experiments using PhosPropels generated with multiple orthogonal proteases is recommended. Because pSer, pThr, and pTyr sites are not equally represented in PhosPropels, the method is not designed to quantify absolute catalytic preference among phosphosite classes. Rather, it enables statistical characterization of sequence-context preferences within each class. The assay does not directly report catalytic efficiency under physiological conditions or distinguish between kinetic and binding contributions to specificity. Accordingly, PhosPropel provides information about intrinsic sequence specificity that can be integrated with complementary structural, biochemical, and cellular approaches to understand phosphoeraser function in signaling networks.

## Materials and Methods

### Phosphopeptide Enrichment.

Phosphopeptides for profiling assays and quantitative proteomics were generated from the human proteome by protease digestion of protein extracts followed by TiO_2_ enrichment.

### Expression and Purification of Recombinant Enzymes.

Recombinant enzymes were expressed in *E. coli* as His_6_-tagged proteins and purified by Ni-NTA chromatography.

### PhosPropel Profiling Assays.

Phosphopeptides were incubated with recombinant enzymes as described in the *SI Appendix*.

### Mass Spectrometry.

Samples were analyzed by LC–MS/MS, and peptides were identified and/or quantified as detailed in the *SI Appendix*.

### Statistical Analysis.

Residue-position enrichment and depletion were quantified using position-specific z-scores comparing enzyme-treated and matched untreated controls. Details of the statistical framework are provided in *SI Appendix*, Supplementary Note 1.

### Biochemical Assays.

Phosphate release assays were performed using the EnzChek Phosphate Assay Kit (Thermo Fisher Scientific) according to the manufacturer’s instructions.

Detailed methods are described in the *SI Appendix*.

## Supplementary Material

Appendix 01 (PDF)

## Data Availability

Proteomics Data, Microscopy images, plasmid maps, Alphafold models, analysis code data have been deposited in ProteomeXchange, Dryad, Zenodo, GitHub (ProteomeXchange: PXD067199 (54), PXD067202 (55), PXD067205 (56), PXD067206 (57), PXD067209 (58), PXD067267 (59), PXD074748 (60), PXD074909 (61), PXD074953 (62), PXD074951 (63), PXD074779 (64), PXD074774 (65), PXD074791 (66); Dryad DOI: 10.5061/dryad.95x69p8z0 (67); Zenodo DOI: 10.5281/zenodo.16785439 (68); GitHub URL: https://github.com/aweeks8/phospropel) (69).
