# Supplementary material for: Phosphoproteome-derived peptide libraries for deep specificity profiling of phosphatases and phospholyases
Source: Proc Natl Acad Sci U S A. 2026 Jun 12;123(24):e2523183123. doi: 10.1073/pnas.2523183123 (PMC13273362; doi:10.1073/pnas.2523183123)
Supplement: Supplementary file 1 — Appendix 01 (PDF) [file pnas.2523183123.sapp.pdf]

PNAS

**Supporting Information for**
# Phosphoproteome-derived peptide libraries for deep specificity profiling of phosphatases and phospholyases

Katarzyna Radziwon[†], Laura A. Campbell[†], Lauren E. Mazurkiewicz, Sopo Jalalishvili, Izabelle Eppinger, Aanika Parikh, and Amy M. Weeks*

Correspondence: Amy M. Weeks
Email: amweeks@wisc.edu

**This PDF file includes:**

SI Text
Materials and Methods
Figures S1 to S51
Tables S1 to S3
Supplementary Appendix
Supplementary Note 1
SI References

**Other supporting materials for this manuscript include the following:**

Datasets S1 to S42
Deposited to the Dryad repository under DOI: 10.5061/dryad.95x69p8z0

Proteomics raw data
Deposited to theProteomeXchange repository under accession numbers listed in Table S3

Analysis code
Github repository: https://github.com/aweeks8/phospropel
Archival, citable version: DOI: 10.5281/zenodo.16785439

Interactive notebook
Google Colab:
https://colab.research.google.com/drive/1z0EJLxZw_3K2Fey708cgrAeFc4LOzC47?usp=sharing.

## SI Text

### Additional analysis of tryptic libraries

To test whether the use of trypsin as the digest protease limits the interrogation of phosphatase/phospholyase recognition of Lys/Arg in positions proximal to phosphosites, we quantified positional amino acid frequencies within the libraries. These data are shown in **Fig. S8-S14**. Although trypsin cleaves C-terminal to Lys and Arg, incomplete digestion under standard proteomics conditions results in frequent missed cleavages (10-30% per the manufacturer, Promega), resulting in frequent retention of Lys/Arg within peptides. These missed cleavage sites occur most commonly when Lys/Arg is flanked by Pro, especially in the P1' or P2' position, or by acidic residues including Asp, Glu, and pSer, or in the context of tandem basic residues (**Fig. S51**). We therefore examined the representation of Lys and Arg at each position surrounding the phosphosite to determine whether sufficient sampling depth is maintained for downstream specificity analysis. Across positions, Lys and Arg are generally well-represented in tryptic libraries. For Lys, we observe on average 278 phosphosites with Lys represented in each flanking position, although there is some frequency variation from site to site. For Arg, we observed an average of 378 peptides with Arg at each position with similar site variation (**Fig. S13**). Additional discussion of library composition and assay sensitivity is included in **Supplementary Note 1**.

### Comparison with alternative proteases

To compare with trypsin, we generated libraries using GluC, LysC, and chymotrypsin. The raw number of observable sites with Lys/Arg in each position was lower in these alternative libraries, consistent with reduced peptide identification relative to the tryptic library (**Fig. S13**). Tryptic peptides are more readily detected by LC–MS/MS for two main reasons: cleavage after Lys and Arg generates C-terminal basic residues that promote efficient ionization and produce strong b- and y-ion series during fragmentation, and the higher cleavage frequency produces peptides within the optimal length range (7–20 amino acids) for MS analysis. GluC and chymotrypsin do not produce peptides with C-terminal basic residues, and although LysC does, trypsin uniquely generates Arg-terminated peptides and a more favorable peptide length distribution, enhancing detectability.

Missed cleavages occurred frequently in GluC libraries at positions that were flanked by additional basic residues or Pro. In LysC libraries, missed cleavages were less frequent and occurred commonly at tandem Lys residues or positions flanked by acidic residues or Pro (**Fig. S51**).

To experimentally evaluate the impact of protease choice, we repeated specificity profiling experiments using libraries produced with GluC, LysC, and/or chymotrypsin. Experiments were performed at the longest time point evaluated for each phosphatase/phospholyase to maximize dynamic range and substrate depletion. Across enzymes, the qualitative specificity patterns that we observed were consistent with those observed in the tryptic library. In some cases, residue-position combinations that were statistically significant in the tryptic dataset did not reach the same significance threshold in alternative libraries, likely reflecting reduced sampling depth due to a smaller number of detectable peptides rather than altered phosphatase/phospholyase specificity. Importantly, the sequence-context preferences within each phosphosite class are well-correlated across protease

conditions. Data for specificity profiling with PhosPropels generated from alternative proteases and correlations between z-scores calculated from these data are shown in **Fig. S19-S20** (λ phosphatase), **Fig. S22-S24** (PTP1B), **Fig. S27-S29** (PP2Ac), **Fig. S32, S34-S35** (WipA), **Fig. S33, S36-S37** (WipB), **Fig. S39-S40** ($OspF_{1-239}$), and **Fig. S43-S44** ($OspF_{27-239}$).

To assess the consistency of z scores across libraries, we compared the z scores to one another using scatterplots in which we introduced thresholds at z = ±4 (α = 0.0001, dotted lines) to help distinguish between consistent signals (significant same-sign z scores), library-specific detection (significant z scores in only one dataset), and signal discordance between libraries (significant z scores of opposite sign in different libraries). Across the enzymes assessed in this manuscript, z scores were generally well-correlated, with sign discordance observed only rarely (3 residue-position combinations across all experiments in the paper; see below for more discussion of these cases).

Although z scores were generally well-correlated, we also observed some cases in which phosphatase-dependent enrichment of residues recognized by the digest protease was only detectable in libraries generated using a different protease. For example, for WipB and PP2Ac, it was clear that phosphosites flanked by acidic residues accumulated in tryptic and LysC libraries, but not in GluC libraries. This effect could be due to depletion of Asp and Glu at internal positions, or to lower detectability of multiply negatively charged peptides in positive-mode LC-MS/MS.

Similar effects were observed for Lys/Arg-containing features, which in some cases were more detectable in GluC-derived libraries, although these trends were less systematic. This effect is likely mitigated in trypsin- and LysC-derived libraries by missed cleavages, as well as by the overall larger number of peptides detected in these datasets. For example, enrichment of Lys at the −2 and −3 positions in the λ phosphatase dataset (**Fig. S19**), illustrates this effect. These features are most strongly detected in the GluC-derived library, are not detected in the LysC-derived library, and are only weakly or partially detected in the trypsin-derived library (K at −3 detected; K at −2, z = 0.96). The reduced detectability in LysC and tryptic libraries is consistent with depletion of internal Lys residues due to protease cleavage, an effect expected to be more pronounced for LysC given its lower missed cleavage rate relative to trypsin.

We also note that LysC- and GluC-derived libraries contained a higher proportion of pTyr sites, which appeared to increase sensitivity for detecting pTyr-associated features. This increase in pTyr proportion may result from 1) improved LC retention of longer peptides; and 2) increased hydrophobicity compared to pSer/pThr, which may improve ionization efficiency in positive-mode LC-MS. As a result, pTyr phosphatase specificity features were more frequently detected as significant in these datasets, reflecting differences in peptide detection in the libraries.

**Flanking window size, length, and phosphosite position.**

We selected a -4 to +4 window based on both precedent and practical considerations. Many kinase and phosphatase specificity analyses focus on a similar local sequence context (typically ±3 to ±7 residues), as residues immediately flanking the phosphosite most strongly influence substrate recognition. Expanding the window substantially beyond

this range increases the number of residue-position combinations tested and therefore reduces statistical power at distal positions due to lower effective sampling depth.

To evaluate whether peptide length or phosphosite positioning constrains this analysis window, we examined both the peptide length distribution and the distance of each phosphosite from the N- and C-termini of the tryptic fragment (**Fig. S15–S18**). While peptides span a broad length distribution, phosphosites were on average 8 residues from the peptide N terminus (**Fig. S16**), 7-10 residues from the peptide C terminus (**Fig. S17**), and 4-5 residues from the nearer terminus (**Fig. S18**). Thus, expanding substantially beyond a ±4 window would result in inconsistent representation of distal positions across peptides, reducing effective coverage and statistical power. In contrast, the -4 to +4 range is robustly represented across the majority of sites in our libraries. We therefore chose not to focus on a wider window by default. However, we note that the *phospropel* Python package is modular and the parameters could easily be adjusted to support different windows.

## Materials and Methods

**Key chemicals and materials.** Synthetic phosphopeptides human aquaporin-2 (254-267, VELH-pSer-PQSL, cat. no. AS-61328), CK1 peptide substrate (KRRRAL-pS-VASLPGL, cat. no. AS-63797), PKC substrate 4 (KR-pThr-IRR, cat. no. AS-20291), tyrosine kinase peptide 3 (RRLIEDAE-pY-AARG, cat. no. AS-24546), and UOM9 PKC substrate (KRP-pSer-QRHG, cat. no. AS-20294) were purchased from Anaspec. Erk activation loop peptide GFL-pThr-E-pTyr-V-amide and serine variant GFL-pSer-E-pTyr-V-amide were synthesized as described previously or purchased from GenScript as the acetate salt at ≥95% purity. MAPK11 (EEM-pThr-G-pTyr-V-amide) and MAPK12 (SEM-pThr-G-pTyr-V-amide) peptides were purchased from GenScript as the acetate salt at ≥95% purity. Sequencing-grade trypsin was purchased from Promega. TiO2 beads were purchased from GL Sciences (Titansphere, 5 μm, cat. No. 5020-75000). Solid-phase extraction disks (C8 or C18, Empore) were purchased from CDS Analytical. LC-MS grade water, acetonitrile, trifluoroacetic acid (TFA), formic acid (FA), and methanol were purchased from Fisher Scientific (Optima grade). Lambda phosphatase (λPP) was purchased from New England Biolabs (cat. No. P0753). Recombinant human PP2Ac (L309 deletion mutant) was purchased from Cayman Chemical (item no. 10011237). EnzChek phosphate assay kit was purchased from Thermo Fisher Scientific. Okadaic acid was purchased from Cayman Chemical (item no. 10011490).

**Molecular biology and plasmid construction.** *E. coli* codon-optimized genes encoding *Legionella pneumophila* WipA (RefSeq: WP_010948417.1) and WipB$_{1\text{-}364}$ (RefSeq: WP_010946379.1), *Shigella flexneri* OspF (RefSeq: WP_010921598.1), *Salmonella typhimurium* SpvC (RefSeq: WP_001122242.1), and *Pseudomonas syringae* HopAI (RefSeq: WP_003365117.1) were purchased from Integrated DNA Technologies or Twist Bioscience. A human codon-optimized gene encoding OspF was purchased from Twist Bioscience. *E. coli* XL10 was using as the cloning host. Oligonucleotides were purchased from Integrated DNA Technologies. All plasmid sequences were confirmed via Sanger sequencing performed by Quintara Biosciences or Functional Biosciences. Plasmid maps have been deposited in the Dryad repository (DOI: 10.5061/dryad.95x69p8z0).

*pBH4-His-Tev-OspF.* The *E. coli* codon-optimized gene encoding OspF was inserted into the pBH4 vector between the BamHI and NotI restriction sites using Gibson assembly,

fusing OspF with an N-terminal His tag followed by a TEV protease cleavage site. OspF mutants were generated using site-directed mutagenesis with following reaction mixture composition: forward and reverse oligonucleotides (0.5 μM each; **Table S1**), pBH4-His-Tev-OspF template (200 ng), dNTPs (0.2 mM each), $MgSO_4$ (1.5 mM), KOD Hot Start DNA polymerase buffer (1×), and KOD Hot Start DNA polymerase (0.02 U/μL). The reaction mixtures were placed in a thermocycler for the following temperature cycle: 95 °C for 2 min; 25 cycles of 95 °C for 20 s, 55 °C for 10 s, 70 °C for 6 min; a final extension at 72 °C for 10 min. Reactions were then cooled and digested with DpnI (0.8 U/μl) overnight at 37 °C. *E. coli* XL10 cells were transformed with the digested products and plated on LB-agar containing carbenicillin (50 μg/mL).

*pBH4-His-Tev-SpvC.* The *E. coli* codon-optimized gene encoding SpvC was inserted into the pBH4 vector between the BamHI and NotI restriction sites using Gibson assembly, fusing SpvC with an N-terminal His tag followed by a TEV protease cleavage site.

*pBH4-His-Tev-HopAI.* The *E. coli* codon-optimized gene encoding HopAI was inserted into the pBH4 vector between the BamHI and NotI restriction sites using Gibson assembly, fusing HopAI with an N-terminal His tag followed by a TEV protease cleavage site.

*pET28a-WipA-His.* The *E. coli* codon-optimized gene encoding WipA was inserted into the pET28a vector between the NcoI and NotI restriction sites using Gibson assembly to generate a construct with a C-terminal His tag.

*pBH4-His-Tev-WipB$_{1\text{-}364}$.* The *E. coli* codon-optimized gene encoding WipB$_{1\text{-}364}$ was inserted into the pBH4 vector between the BamHI and NotI restriction sites using Gibson assembly to fuse WipB with an N-terminal His tag followed by a TEV protease cleavage site.

*pcDNA5/FRT/TO-V5-OspF.* The human codon-optimized gene encoding V5-OspF was inserted into the pcDNA5/FRT/TO vector (Invitrogen) between the KpnI and NotI restriction sites using Gibson assembly.

**Cell culture.** HEK293T cells (ATCC #CRL-3216) and K562 cells (ATCC #CCL-243) were grown in DMEM with 10% (v/v) fetal bovine serum and 100 U/mL penicillin, 100 μg/mL streptomycin at 37°C under a 5% $CO_2$ atmosphere. Jurkat E6.1 (ATCC #TIB-152) cells were grown in RPMI-1640 media with 10% fetal bovine serum and 100 U/mL penicillin, 100 μg/mL streptomycin at 37ºC under 5% $CO_2$ atmosphere. Flp-In TRex 293T cells (Thermo Fisher Scientific) were grown in DMEM with 10% tetracycline-free fetal bovine serum, 100 U/mL penicillin, 100 μg/mL streptomycin, and other antibiotics as appropriate. Cells were tested every six months for mycoplasma contamination using the LookOut Mycoplasma PCR Detection Kit (Sigma-Aldrich) according to the manufacturer's instructions.

*Construction of a stable cell line for inducible OspF expression*. Flp-In TRex 293T cells were transfected with pcDNA5/FRT/TO-V5-OspF according to the manufacturer's instructions to generate a stable cell line (Flp-In TRex 293T-V5-OspF) with V5-OspF under a doxycycline-inducible promoter.

*Immunofluorescence.* Flp-In TRex 293T-V5-OspF cells ($5 \times 10^4$ cells) were seeded on glass-bottom 24-well imaging dishes coated with poly-D-lysine and were grown for 24

hours at 37°C under a 5% $CO_2$ atmosphere prior to induction. Media was then replaced with fresh media containing 1 μg/mL doxycycline to induce OspF expression for 18 h. Media was removed, and cells were fixed with 4% paraformaldehyde in PBS at 4°C for 45 min. Cells were then washed three times with PBS and permeabilized with cold methanol (pre-chilled at -20°C) for 5 min. Cells were washed three times with PBS and mouse anti-V5 antibody (Invitrogen #46-0705) in PBS + 3% BSA was added at a 1:2,000 dilution. Cells were incubated with the antibody for 1 h at 4°C. Cells were than washed three times with PBS and stained with DAPI (1:1,000 dilution) and goat anti-mouse-AlexaFluor 647 (1:1,000, Invitrogen #A-21236) in PBS + 3% BSA for 1 h at 4°C. Cells were washed three times with PBS prior to imaging on a Nikon Ti2-E inverted epifluorescence microscope. Raw images have been deposited in the Dryad repository (DOI: 10.5061/dryad.95x69p8z0).

**Generation of human proteome-derived peptide libraries (PhosPropels).** Human proteome-derived peptide libraries were produced following a modified version of the protocol of Jersie-Christensen et al.[1] as described briefly below.

*Pervanadate treatment.* Pervanadate stock solution (50 mM) was made fresh prior to each use by mixing equal volumes of 100 mM hydrogen peroxide and 100 mM sodium orthovanadate. For treatment of HEK293T cells with pervanadate, media was aspirated from a 150 mm dish of cells at 90% confluency and cells were washed once with DPBS (20 mL). Serum-free DMEM (20 mL) was then added to the cells and pervanadate was added to a final concentration of 100 μM. Cells were then incubated at 37ºC under 5% $CO_2$ atmosphere for 1 h. Pervanadate treatment caused some cells to detach; these cells were transferred to a 50 mL centrifuge tube and harvested by centrifugation at 400 × g for 5 min. The remaining cells were treated with trypsin/EDTA (0.25%, 5 mL, Gibco) for 3 min, transferred to the same centrifuge tube, and harvested by centrifugation at 400 × g for 5 min. For Jurkat and K562 cells, cells from a 150 mm dish were collected by centrifugation at 400 × g for 5 min, washed once with DPBS, and resuspended in the appropriate serum-free media with 100 μM pervanadate. Cells were replated in a 150 mm dish and incubated at 37ºC under 5% $CO_2$ atmosphere for 1 h. Cells were then harvested by centrifugation at 400 × g for 5 min and serum-free media was aspirated.

*Cell lysis.* One 150 mm dish of cells at 90% confluency (HEK293T) or at a concentration of $10^6$ cells per mL (Jurkat and K562, 40 mL) was used for each replicate. The cell pellet was resuspended in 1 mL hot lysis buffer (100 mM Tris-HCl, pH 8.0, 6 M guanidine hydrochloride, 5 mM TCEP, 10 mM chloroacetamide) that was preheated to 95°C and the suspension was heated at 95°C for 10 min. Cells were then subjected to 10 cycles of probe ultrasonication (20% amplitude; 5 s on / 5 s off) to complete lysis. Insoluble material was removed by centrifugation at 20,000 × g for 10 min at 4°C.

*Trypsin digestion.* The cell lysate was diluted with 4 volumes (4 mL) of 25 mM Tris-HCl, pH 8.0 and incubated for 12-16 h at 37°C with 20 µg sequencing grade modified trypsin (Promega). The digested lysates were then acidified by adding TFA to a final concentration of 1% (v/v) to reduce the pH to <3. Insoluble material was removed by centrifugation at 5,000 × g for 5 min at 4°C. The digested peptides were desalted on a Sep-Pak C18 Plus Short Cartridge (360 mg sorbent per cartridge, Waters). The cartridge was conditioned with 100% acetonitrile (3 mL) and equilibrated with 2 × 3 mL 0.1% TFA in water. Digested peptides were then loaded onto the cartridge. The cartridge was washed with 2 × 3 mL 0.1% TFA in water. Peptides were eluted with 3 mL 40% acetonitrile/60% water/0.1% TFA followed by 3 mL 60% acetonitrile/40% water/0.1% TFA.

The volume of the eluted peptides was reduced by twofold using a vacuum centrifuge. Peptide concentration was estimated by measuring absorbance at 280 nm (1 mg/mL = 1 absorbance unit, 1 cm path length) on a Nanodrop spectrophotometer.

*Phosphopeptide enrichment*. The desalted peptides were then transferred to a conical tube and its volume was doubled with the enrichment buffer (80% acetonitrile, 12% TFA). $TiO_2$ bead slurry was prepared by suspending Titansphere $TiO_2$ beads (GL Sciences) in bead buffer (20 mg/mL 2,5-dihydroxybenzoic acid (DHB), 80% acetonitrile, 6% TFA). The slurry was then added to the tryptic peptides in 1:2 sample:bead ratio (w/w). The sample was incubated on a rotator for 15 min and then centrifuged to collect the beads. The beads were resuspended in 50 uL of buffer A (10% acetonitrile, 6% TFA), transferred to a 1.5 mL microcentrifuge tube and centrifuged at 500 × g for 2 min. The beads were then resuspended in fresh buffer A and transferred to a homemade C8 single-layered StageTip[2]. The StageTip was centrifuged at 1,000 × g at room temperature for 2 min to remove buffer A. The StageTip was then washed with 2 × 50 μL buffer B (40% acetonitrile, 6% TFA) and 2 × 50 μL buffer C (60% acetonitrile, 6% TFA), with each wash step being carried out by centrifugation at 1,000 × g for 2 min. Phosphopeptides were eluted with 20 μL elution buffer 1 (5% ammonium hydroxide) followed by 20 μL elution buffer 2 (10% ammonium hydroxide). The eluate was concentrated in a vacuum centrifuge to near-dryness. Phosphopeptides were desalted on SOLA HRP SPE cartridges (ThermoFisher Scientific) according to the following protocol: The column was conditioned with 500 μL acetonitrile and equilibrated with 2 × 1 mL 0.1% TFA. The sample (acidified to pH < 3 with 1% TFA and diluted to 500 μL with 0.1% TFA) was loaded onto the column and column was washed with 2 × 1 mL 0.1% TFA. The desalted phosphopeptides were eluted from the column with 2 × 150 μL 80% acetonitrile with no TFA added. The sample was concentrated to near-dryness in a vacuum centrifuge, resuspended in LC-MS grade water at a concentration of 0.5-1.0 mg/mL, and stored at -20°C.

**Expression and purification of WipA and WipB.** Sequences of expressed proteins are listed in **Table S2**. Chemically competent *E. coli* C43(DE3) cells were transformed with the appropriate plasmid for overexpression. Starter cultures (10 mL LB supplemented with 50 μg/mL kanamycin for WipA or 50 μg/mL carbenicillin for WipB) were inoculated with single colonies and grown overnight at 37°C with shaking at 200 rpm. Starter cultures were used to inoculate expression cultures (1 L LB supplemented with the appropriate antibiotic) in baffled flasks. Cutures were grown at 37°C with shaking at 180 rpm until they reached $OD_{600\ nm}$ of 0.4-0.6. Expression was then induced by addition of IPTG to a final concentration of 1 mM. Expression was allowed to proceed overnight at 16°C with shaking at 180 rpm. Cells were harvested by centrifugation at 4,000 × g for 20 min at 4°C and resuspended in lysis buffer (25 mM Tris-HCl, pH 7.5, 300 mM NaCl, 10 mM imidazole, 1 mM TCEP, 5% glycerol, with one Complete EDTA-Free Protease Inhibitor Tablet (Roche) per 25 mL). Cells were lysed by three passes through an Emulsiflex microfluidizer at 15,000 psi. The lysate was cleared by centrifugation at 10,000 × g for 30 min at 4°C. Clarified lysate was then added to 1 mL of Ni-NTA resin pre-equilibrated with lysis buffer. His tagged proteins were allowed to bind to the resin for 1 h on a nutator at 4°C. The resin was collected by centrifugation at 1,000 × g for 2 min at 4°C, transferred to a 15 mL column, and washed with 20 mL lysis buffer followed by 20 mL wash buffer (25 mM Tris-HCl, pH 7.5, 1 M NaCl, 10 mM imidazole, 1 mM TCEP, 5% glycerol). Protein was then eluted with 2-3 × 5 mL elution buffer (25 mM Tris-HCl, pH 7.5, 150 mM NaCl, 600 mM imidazole, 1 mM TCEP, 5% glycerol). For WipB, TEV protease (1:50 molar ratio) was added prior to dialysis again dialysis buffer (25 mM Tris-HCl, pH 7.5, 150 mM NaCl, 1 mM

TCEP, 5% glycerol) overnight. WipA was dialyzed against the same buffer in the absence of TEV protease. After dialysis, WipB was passed over a Ni-NTA column to removed uncleaved protein and protease. Purified proteins were aliquoted, flash frozen in liquid nitrogen, and stored at -80°C until futher use. Purity was assessed by SDS-PAGE and ESI-TOF MS analysis (**Figure S49**).

**Expression and purification of OspF, SpvC, HopAI, and OspF variants.** Sequences of expressed proteins are listed in **Table S2.** Chemically competent *E. coli* BL21(DE3) cells were transformed with the appropriate plasmid for overexpression. Starter cultures (10 mL Luria-Bertani (LB) media, 50 µg/mL carbenicillin) were inoculated with single colonies and grown overnight at 37°C with shaking at 200 rpm. Starter cultures were used to inoculate expression cultures (1 L LB, 50 µg/ml carbenicillin) in baffled flasks. Cultures were shaken at 180 rpm at 37°C until they reached $OD_{600}$ ~0.6. Expression was then induced with 1 mM IPTG (isopropyl-β-D-thiogalactopyranoside). The expression was carried out overnight at 16°C with shaking at 180 rpm. Cell pellets were harvested by centrifugation and resuspended in 40 mL lysis buffer (25 mM Tris pH 8.0, 200 mM NaCl, 5 mM imidazole, pH 8.0, 300 µM TCEP). Lysis was achieved by three passes through an Emulsiflex microfluidizer at 15000 psi. The lysate was centrifuged at 10,000 × g for 30 min at 4°C. Batch binding with HisPur Ni-NTA resin (1 mL, ThermoFisher Scientific) was allowed for 1 h at 4°C on a nutator. Afterwards, the resin was collected by centrifugation at 1,000 × g for 2 min at 4°C, transferred to a 15 mL column, and washed with 20 mL lysis buffer and 20 mL wash buffer (25 mM Tris pH 8.0, 300 mM NaCl, 20 mM imidazole, pH 8.0, 300 µM TCEP). Protein was eluted with 10 mL elution buffer (25 mM Tris pH 8.0, 150 mM NaCl, 250 mM imidazole, pH 8.0, 300 µM TCEP) and dialyzed overnight against 2 L dialysis buffer (25 mM Tris pH 8.0, 150 mM NaCl, 300 µM TCEP). Dialyzed protein was concentrated in an Amicon Ultra-4 centrifugal filter (10,000 NMWL) (Millipore) prior to purification by size exclusion chromatography on Superdex 75 Increase 16/60 column (Cytiva) using size exclusion buffer (25 mM Tris pH 8.0, 150 mM NaCl, 300 µM TCEP). Fractions containing pure recombinant protein were pooled, concentrated, and flash frozen in liquid nitrogen. Following the purification, the protein purity was analyzed by SDS-PAGE and ESI-TOF MS (**Figure S50**). The protein concentration was determined by absorbance at 280 nm, with extinction coefficients determined by ProtParam.

**Treatment of PhosPropels with phosphoeraser enzymes.** Specific conditions for reactions with each enzyme are described below. After quenching, reactions were desalted using double-layer C18 StageTips made in house according to the protocol of Rappsilber et al.[50]. Buffers and samples were passed through the StageTip by centrifugation at 1,000 × g for 1 min. The tip was conditioned with 50 µL of acetonitrile and equilibrated with 2 × 50 µL 0.1% TFA. The sample was then loaded. The tip was then washed with 2 × 50 µL 0.1% TFA, 1 × 50 µL 5% methanol, 0.1% TFA, and 1 × 50 µL 2% acetonitrile, 0.1% FA. Desalted peptides were eluted with 2 × 30 µL 80% acetonitrile, 0.1% FA. Samples were concentrated to near-dryness in a vacuum centrifuge and then resuspended in 13 µL 2% acetonitrile, 0.1% FA. The concentration was estimated by measuring the sample absorbance at 280 nm on a Nanodrop spectrophotomer (1 mg/mL = 1 absorbance unit with a 1 cm path length).

*λ phosphatase (λPP).* λPP was purchased from New England Biolabs (cat. No. P0753). PhosPropels (0.1-0.2 mg/mL) were treated with λPP (30 units) in 50 mM HEPES, pH 7.5, 100 mM NaCl, 2 mM DTT, 1 mM $MnCl_2$ at 30°C in a total volume of 50 µL. The reaction was quenched after 5 min, 30 min, or 180 min by addition of 10 µL of 1%TFA.

*PTP1B1-321*. PTP1B1-321 was purified as described previously[3]. PhosPropels (0.1-0.2 mg/mL) were treated with PTP1B1-321 (20 nM) in 10 mM Tris-HCl, pH 7.5, 25 mM NaCl, 1 mM EDTA, 1 mM DTT at 37°C in a total volume of 50 μL. The reaction was quenched after 5 min, 30 min, or 60 min by addition of 10 μL of 1%TFA.

*PP2Ac*. Recombinant human PP2Ac (L309 deletion mutant) was purchased from Cayman Chemical (item no. 10011237). PhosPropels (0.1-0.2 mg/mL) were treated with PP2Ac (4 mU/mL) in 40 mM Tris-HCl, pH 7.5, 34 mM $MgCl_2$, 4 mM EDTA, 2 mM DTT, 0.05 mg/mL BSA at 30°C in a total volume of 50 μL. The reaction was quenched after 5 min, 30 min, or 180 min by addition of 10 μL of 1%TFA. For inhibition experiments, okadaic acid was included at concentrations between 1 nM and 350 nM.

*WipA*. PhosPropels (0.1-0.2 mg/mL) were treated with WipA (100 nM) in 25 mM Tris-HCl, pH 7.5, 150 mM NaCl, 1 mM DTT, 1 mM $MnCl_2$ at 37°C in a total volume of 50 μL. The reaction was quenched after 5 min, 30 min, or 120 min by addition of 10 μL of 1%TFA.

*WipB*. PhosPropels (0.1-0.2 mg/mL) were treated with WipB (100 nM) in 25 mM Tris-HCl, pH 7.5, 150 mM NaCl, 1 mM DTT, 1 mM $MnCl_2$ at 37°C. The reaction was quenched after 5 min, 30 min, or 120 min by addition of 10 μL of 1%TFA.

**LC-MS/MS data collection for PhosPropel samples.** LC-MS/MS data were collected using an UltiMate 3000 RSLCnano liquid chromatography system in line with an Orbitrap Exploris 480 hybrid quadrupole-Orbitrap mass spectrometer (ThermoFisher Scientific). Peptides (0.5-1 μg dissolved in 2% acetonitrile, 0.1% FA, 5 μL total injection volume) were injected onto an Acclaim PepMap RSLC column (75 µm × 50 cm, 2 µm particle size, 100 Å pore size, ThermoFisher Scientific) over 15 min in 97% mobile phase A (0.1% FA) and 3% mobile phase B (0.1% FA, 80% acetonitrile) at 0.3 μL/min. The column was eluted using a linear gradient from 3% mobile phase B to 50% mobile phase B over 120 min at 0.3 μL/min. The eluate was electrosprayed through a nanospray emitter tip by applying 2000 V through the ion source's DirectJunction adapter. Full MS scans were performed over a range of 350-1,200 m/z at a resolution of 60,000 at 200 m/z. The AGC target was set to 300% and the maximum injection time was set to 'auto'. The top 20 most abundant precursors with a charge state of 2-6 were selected for MS/MS analysis using an isolation window of 1.4 m/z and a precursor intensity threshold of $5 \times 10^3$. A 20 s dynamic exclusion window with a precursor mass tolerance of ± 10 ppm was applied. MS/MS scans used HCD fragmentation with a normalized collision energy of 30%, a resolution of 15,000 and a fixed first mass of 110 m/z. The AGC target was set to 'standard' and the maximum injection time was set to 22 ms.

**LC-MS/MS data analysis for PhosPropels.** Thermo RAW files were searched against the human SwissProt database (downloaded 01/24/2020)[4] using the SEQUEST[5] algorithm in Proteome Discoverer 2.4 (ThermoFisher Scientific). The precursor mass tolerance was set to 10 ppm and the fragment mass tolerance was set to 0.02 Da. Search parameters included the following modifications: carbamidomethylation at Cys (+57.021 Da, static), oxidation at Met (+15.995 Da, dynamic), acetylation at protein N termini (+42.011 Da, dynamic), Met loss at protein N termini (-131.040 Da, dynamic), Met loss+acetylation at protein N termini (-89.030 Da, dynamic), phosphorylation at Ser, Thr, and Tyr (+79.966 Da, dynamic), and dehydration at Ser and Thr (-18.011 Da, dynamic). Up to two missed cleavages were allowed. PSM validation was performed with

the Percolator[6] node of Proteome Discoverer 2.4 at a false discovery rate of 1%. Localization of modification sites was scored using the IMP-ptmRS[7] node with PhosphoRS set to false. Raw data, peak lists, and results have been deposited in the ProteomeXchange repository under the accession numbers listed in **Table S3**. Results are also provided in Microsoft Excel format as **Supplementary Datasets S1-S16** as described in **Table S3.**

**Analysis of PhosPropel data for phosphoeraser specificity profiling.** Z-scores for each amino acid in each position were calculated using custom Python scripts available in the Zenodo repository (DOI: 10.5281/zenodo.16785439). Z-scores compared the position-specific frequencies of each amino acid in each position surrounding the phosphosite to frequencies in an appropriate experimentally measured background set. For each position and amino acid, z-scores were calculated as described below. Each phosphorylation site was treated independently as the central phosphosite. Z-scores were assumed to follow a standard normal distribution under the null hypothesis, which is appropriate given the large number of observations contributing to each frequency estimate. Z-scores are plotted directly in the heatmaps shown for each specificity profile, colored according to their magnitude and direction. Two-tailed p-values were computed from the z-scores using the standard normal distribution. Multiple hypothesis testing correction was performed using the Benjamini-Hochberg method to control the false discovery rate at a = 0.0001. Position-amino acid combinations that had statistically significant frequency differences between the sample and the control are outlined in black in the heatmaps. The statistical framework for this analysis is described in detail in **Supplementary Note 1**.

*PhosPropel positional composition.* To generate the z-score heatmaps shown in **Fig. 1D**, phosphosites identified with ptmRS localization scores >90 were aligned and the positional frequencies of each amino acid in each position were calculated. The global (position-independent) frequency of each amino acid (including pSer, pThr, and pTyr) observed in each sample was then calculated. Z-scores were then calculated according to the equation

$$z = \frac{f_{positional} - f_{global}}{\sigma}$$

where $f_{positional}$ is the frequency of each amino acid in each position surrounding the phosphosite, $f_{global}$ is the global frequency, and $\sigma$ is the population standard deviation,

$$\sigma = \sqrt{\frac{f_{global}(1-f_{global})}{n}}.$$

*PhosPropel phosphatase specificity profiles.* To generate the z-score heatmaps shown for phosphatase specificity profiles, phosphosites identified with ptmRS localization scores >90 were aligned and the positional frequencies of each amino acid in each position were calculated. Z-scores were then calculated by comparing positional frequencies at each time point to positional frequencies at the 0 min time point according to the equation

$$z = \frac{f_{phosphatase} - f_{control}}{\sigma}$$

where $f_{phosphatase}$ is the frequency of each amino acid in each position surrounding the phosphosite following phosphatase treatment for the specified time, $f_{control}$ is the frequency of each amino acid in each position at the 0 min time point, and $\sigma$ is the population standard deviation,

$$\sigma = \sqrt{\frac{f_{phosphatase}(1-f_{phosphatase})}{n_{phosphatase}} + \frac{f_{control}(1-f_{control})}{n_{control}}}.$$

*PhosPropel phospholyase specificity profiles.* To generate the z-score heatmaps shown for phospholyase specificity profiles, β-elimination sites identified with ptmRS localization scores >90 were aligned and the positional frequencies of each amino acid in each position surrounding the β-elimination site were calculated. Separately, pSer and pThr sites with ptmRS localization scores >90 were aligned and the positional frequencies of each amino acid in each position surrounding the phosphosite were calculated. Only pSer and pThr sites were used because β-elimination can only occur at pSer and pThr sites. Z-scores were then calculated by comparing positional frequencies for β-elimination sites versus phosphosites according to the equation

$$z = \frac{f_{\beta-elimination} - f_{phosphosite}}{\sigma}$$

where $f_{\beta\text{-elimination}}$ is the frequency of each amino acid in each position surrounding β-elimination sites, $f_{phosphosite}$ is the frequency of each amino acid surrounding phosphosites, and $\sigma$ is the population standard deviation,

$$\sigma = \sqrt{\frac{f_{\beta-elimination}(1-f_{\beta-elimination})}{n_{\beta-elimination}} + \frac{f_{phosphosite}(1-f_{phosphosite})}{n_{phosphosite}}}.$$

*Comparison between OspF variant and wild-type OspF specificity profiles.* To generate the z-score heatmaps shown for comparison of specificity profiles between OspF variants and wild-type OspF, β-elimination sites identified with ptmRS localization scores >90 in the OspF variant-treated samples were aligned and the positional frequencies of each amino acid in each position surrounding the β-elimination site were calculated. Separately, β-elimination sites identified with ptmRS localization scores >90 in the wild-type OspF-treated samples were aligned and the positional frequencies of each amino acid in each position surrounding the β-elimination site were calculated. Z-scores were then calculated by comparing positional frequencies for β-elimination sites in the two samples according to the equation

$$z = \frac{f_{\mathrm{variant}} - f_{wild-type}}{\sigma}$$

where $f_{variant}$ is the frequency of each amino acid in each position surrounding β-elimination sites in the wild-type OspF-treated sample, $f_{wild\text{-}type}$ is the frequency of each amino acid in each position surrounding β-elimination sites in the wild-type OspF-treated sample, and $\sigma$ is the population standard deviation,

$$\sigma = \sqrt{\frac{f_{\text{variant}}(1-f_{\text{variant}})}{n_{\text{variant}}} + \frac{f_{wild-type}(1-f_{wild-type})}{n_{wild-type}}}.$$

**Scoring of Phospropel phosphosites with kinase PSSMs.** Phosphopeptides identified in each Phospropel replicate were scored using position-specific scoring matrices (PSSMs) for Ser/Thr kinases (for pSer/pThr sites) or for Tyr kinases (for pTyr sites) using custom code that is available in the Zenodo repository under DOI: 10.5281/zenodo.16785439. Each site was scored against all kinases by summing the PSSM values reported in ref. [8,9] for each position-amino acid combination in a window four residues upstream and four residues downstream of the site. Kinases with a score ≥2 standard deviations above the mean score for a given sequence were considered as candidate writers of the phosphosite and are included in the dataset. For each replicate, the number of times a given kinase passed this threshold (i.e., the number of potential substrates per replicate) was computed. The average number of substrates per sample was then calculated. The $\log_2$(average number of substrates per replicate) was plotted on the human kinome tree as node size using Coral[10]. For comparison of the average number of potential kinase substrates in untreated and pervanadate-treated libraries, *P* values were calculated using a two-tailed, unpaired *t* test in Prism 10 (GraphPad). *P* values were corrected for multiple comparisons using the Holm-Sidak method available in the Prism 10 software.

**Generation of Alphafold3 models of substrate-bound WipA and WipB.** Structural models of WipA and WipB were generated using Alphafold3[11] accessed at https://alphafoldserver.com/. For WipA, the model included two molecules of WipA, two molecules of the peptide EDAEpYAARG (a validated WipA substrate), and two $Mn^{2+}$ ions. For WipB, the model included two molecules of full-length WipB, two molecules of the peptide RRALpSVASL, and two $Mn^{2+}$ ions. Models were ranked based on their predicted LDDT (pLDDT) confidence scores, and the highest-confidence structure was used for analysis. Models were rendered in PyMOL and have been deposited in the Dryad repository (DOI: 10.5061/dryad.95x69p8z0).

**Preparation of TMT phosphoproteomics samples.**
HEK293T OspF Flp-In cells were grown in 150 mm culture plates in the presence or absence of 1 μg/mL doxycycline for 24 hours. Four biological replicates were performed for each expression condition. Cells were washed with PBS and treated with trypsin/EDTA (0.25%, 5 mL, Gibco) for 3 min, and harvested by centrifugation at 400 × g for 5 min. Proteomic lysis buffer (1 mL per 150 mm culture dish, 100 mM Tris-HCl, pH 8, 6 M guanidine-HCl, 7.5 mM TCEP, 5 mM chloroacetamide) was heated to 95°C then added to cell pellets. Cells in lysis buffer were heated for an additional 10 minutes and then sonicated (10 cycles at 20% amplitude; 5 s on / 5 s off). Lysates were diluted with 25 mM Tris, pH 8.0 such that guanidine-HCl was below 1 M then digested using 20 μg sequencing-grade trypsin (Promega) at 37°C overnight.

Peptide samples were desalted using the Sep-Pak Plus C18 cartridge (Waters) desalting procedure described in **Generation of human proteome-derived peptide libraries (PhosPropels)** above. Dried, desalted peptides were resuspended in 100 mM HEPES, pH 8 and further pH adjusted using 1M HEPES until an approximate pH of 8 was reached. Peptide concentration was assessed using the Pierce Quantitative Colorimetric Peptide Assay Kit (Thermo Fisher Scientific). 400 μg of peptide from each sample was labeled

with 800 μg TMT10plex isobaric labeling reagent (Thermo Fisher Scientific) resuspended in acetonitrile. After incubation at room temperature for 1hr, reactions were quenched using 0.27% hydroxylamine for 15 minutes. Samples were then pooled and dried in a vacuum centrifuge. The dried pooled sample was again desalted using the standard SepPak desalting procedure. The pooled TMT labeled sample was then enriched for phosphopeptides as described in **Generation of human proteome-derived peptide libraries (PhosPropels)** above. Dried, TMT-labeled phosphopeptide samples were desalted using standard SOLA C18 HRP SPE cartridge (Thermo Fisher Scientific) procedure.

**LC-MS/MS data collection for TMT phosphoproteomics samples.** TMT phosphoproteomics samples were analyzed on a Thermo Scientific Ultimate 3000 RSLCnano coupled to a Thermo Scientific Orbitrap Lumos Tribrid. Sample was resuspended 0.1% FA / 5% acetonitrile, and 1 μg of sample was loaded onto a Thermo Fisher Scientific EASY-spray column (500 mm length × 75 μm inner diameter, 2 μm particle size, spherical fully porous ultrapure silica). Peptides were eluted using mobile phase A (0.1% FA) and mobile phase B (80% acetonitrile / 0.1% FA) with the gradient profile: 0 min: 2% B, 3 min: 5% B, 140 min: 25% B, 206min: 62.5% B, 216 min: 95% B. The Lumos MS was operated in positive mode using a 1.8 kV spray voltage and 300°C ion transfer tube temperature. MS1 spectra were collected at 60,000 resolution for a m/z range of 400 – 1,400. MS2 spectra were collected in a 1 s total cycle time using a 0.7 m/z isolation width, 36% normalized HCD collisional energy, 30,000 resolution and a mass range of 110 – 2,000 m/z. A maximum injection time of 50 ms and normalized AGC target of 250% was used for both MS1 and MS2 spectra collection. Dynamic exclusion was set to 30s.

**TMT phosphoproteomics data analysis.** LC-MS/MS data was processed using the Sequest HT[5] algorithm in Proteome Discoverer 2.4 (ThermoFisher Scientific). Data were searched using the human SwissProt FASTA database (downloaded 1/24/2020)[4] with a precursor mass tolerance of 10 ppm and a fragment mass tolerance of 0.6 Da. Static modifications of TMT6plex (+229.163 Da, peptide N-terminal and K) and carbamidomethyl (+57.021 Da, C), as well as dynamic modifications acetyl (+42.011 Da, protein N-terminal), Met-loss (-131.040 Da, M), Met-loss+Acetyl (-89.030 Da, M), oxidation (+15.995 Da, M), and phosphorylation (+79.966 Da, S,T,Y). High confidence peptides were identified with a 0.01 target FDR and medium confidence peptides were identified with a 0.05 target FDR. Quantification of peptide reporter ions was performed in Proteome Discoverer 2.4 (ThermoFisher Scientific) and was normalized based on total peptide amount per channel and p-values were calculated using an ANOVA test based on individual peptides.

**Preparation of LFQ phosphoproteomics samples.**

HEK293T OspF Flp-In cells were grown in 150 mm culture plates in the presence or absence of 1 μg/mL doxycycline for 24 hours. Four biological replicates were performed for each expression condition. Cells were washed with PBS and treated with trypsin/EDTA (0.25%, 5 mL, Gibco) for 3 min, and harvested by centrifugation at 400 × g for 5 min. Proteomic lysis buffer (1 mL per 150 mm culture dish, 100 mM Tris-HCl, pH 8, 6 M guanidine-HCl, 7.5 mM TCEP, 5 mM chloroacetamide) was heated to 95°C then added to cell pellets. Cells in lysis buffer were heated for an additional 10 minutes and then sonicated (10 cycles at 20% amplitude; 5 s on / 5 s off). Lysates were diluted with 25 mM Tris, pH 8.0 such that guanidine-HCl was below 1 M then digested using 20 μg sequencing-grade trypsin (Promega) at 37°C overnight. Following desalting using a Sep-

Pak Plus C18 cartridge, samples were enriched for phosphopeptides as in **Generation of human proteome-derived peptide libraries (PhosPropels)** above.

**LC-MS/MS data collection for LFQ phosphoproteomics samples.** The non-stimulated LFQ samples were analyzed on an UltiMate 3000 RSLCnano system (Thermo Fisher Scientific) coupled to an Exploris 480 mass spectrometer (Thermo Fisher Scientific). 750 ng of sample was loaded onto an Acclaim PepMap C18 100 column (75 µm × 500 mm, 2 µm particle size, 100 Å pore size) and eluted in a 120-minute linear gradient spanning from 3% to 50% solvent B (solvent A = 0.1% FA, solvent B = 80% ACN / 0.1% FA). A spray voltage of 2 kV and an ion source temperature of 325°C was used to collect Exploris data in positive ion mode. A MS1 resolution of 60,000 was used to collect spectra of the mass range 350-1,200 m/z. The MS1 AGC target was set to 300% with "auto" maximum injection time. MS2 spectra were collected at a resolution of 15,000 for the top 20 peaks with a charge state of 2-6. The HCD fragmentation normalized collision energy was set to 30%. Peptides were selected for fragmentation with an isolation width of 1.4 m/z and intensity threshold of $5 \times 10^3$. Dynamic exclusion window was set to 20 s. The MS2 AGC target was set to "standard mode" and a maximum injection time of 22 ms was selected.

EGF-stimulated LFQ samples were analyzed on a Thermo Scientific Ultimate 3000 RSLCnano coupled to a Thermo Scientific Orbitrap Lumos Tribrid mass spectrometer. 1.2 ug of each sample was loaded onto a Thermo Fisher Scientific EASY-spray column (500 mm length x 75 um inner diameter, 2um particle size, spherical fully porous ultrapure silica) and eluted with the following gradient: (min:%B) 0:2, 2:5, 95:37.5, 102:95. The Lumos mass spectrometer was operated in positive mode using a 1.8 kV spray voltage and 300°C ion transfer tube temperature. MS1 spectra were collected at a resolution of 120,000 for a m/z range of 350 – 1,600. The MS1 AGC target was set to 250% with a maximum injection time of 50 ms. MS2 spectra were collected in the linear ion trap using the "Turbo" scan rate in a 1s total cycle time. An isolation width of 0.7 m/z was used with a normalized collision energy of 30% for HCD fragmentation. The MS2 AGC target was set to 300% with a maximum injection time of 25 ms.

**LFQ phosphoproteomics data analysis.** LC-MS/MS data was processed using the Sequest HT[5] algorithm in Proteome Discoverer 2.4 (ThermoFisher Scientific). Data were searched using the human SwissProt FASTA database (downloaded 1/24/2020)[4] with a precursor mass tolerance of 10 ppm and a fragment mass tolerance of 0.6 Da. Carbamidomethyl (+57.021 Da, C) was included as a static modification and acetyl (+42.011 Da, protein N-terminal), Met-loss (-131.040 Da, M), Met-loss+Acetyl (-89.030 Da, M), oxidation (+15.995 Da, M), and phosphorylation (+79.966 Da, S,T,Y) were included as dynamic modifications. High confidence peptides were identified with a 0.01 target FDR and medium confidence peptides were identified with a 0.05 target FDR. Peptides were quantified based on precursor abundance intensity, and quantification values were normalized based on total peptide abundance per sample. An ANOVA test based on individual peptides was used to calculate p-values.

**Phosphate release assays.** Kinetic analysis of phosphate release catalyzed by phosphoerasers was performed using the EnzChek Phosphate Assay Kit (ThermoFisher Scientific) following manufacturer's protocol, with changes in buffer composition according to the requirements of the enzyme under study (described in detail below). Reactions were performed in 100 µL total volume. Assays were conducted in UV-Star UV-Transparent Microplates (Greiner) and absorbance at 360 nm was monitored for 30 min with a Tecan Infinite M200 Pro microplate reader.

*Single substrate concentration rate measurements.* Assays were performed using peptides (AQP2, CK1, PKC, TKP, and UOM9, Erk, Erk (pThr→pSer), MAPK11, or MAPK12) at a final concentration of 100 μM. $PTP_{1\text{-}321}$ was assayed at 20 nM in 1× reaction buffer provided in the EnzChek kit. PP2Ac was assayed at 4 mU/mL in 1× reaction buffer provided in the EnzChek kit. WipA was assayed at 200 nM in 1× reaction buffer supplemented with 1 mM $MnCl_2$. WipB was assayed at 300 nM in 1× reaction buffer supplemented with 1 mM $MnCl_2$. OspF was assayed at 200 nM using a custom reaction buffer (300 mM Tris-HCl, pH 7.5, 1 mM $MgCl_2$).

*OspF variant activity screen.* Peptides GFLpTEpYV and GFLpSEpYV were used at 100 μM final concentration. For alanine scan, OspF was assayed at 0.5 μM for the pThr susbtrate and 2 μM for the pSer substrate. OspF-D219X variants were assayed at a final concentration of 0.5 μM in 300 mM Tris-HCl, pH 7.5, 1 mM $MgCl_2$ for both substrates.

*Michaelis-Menten kinetics of OspF and OspF-D213Q/E219I.* To determine steady-state kinetic parameters of OspF and OspF-E213Q/D219I, substrate concentration was varied between 0-1000 μM for GFLpTEpYV and between 0-2000 μM for GFLpSEpYV. Phosphopeptides were added to a reaction mixture containing 300 mM Tris-HCl, pH 7.5, 1 mM $MgCl_2$, 0.2 mM 2-amino-6-mercapto-7-methylpurine riboside (MESG), and 0.1 U purine nucleoside phosphorylase (PNP). The reactions were started by addition of OspF or OspF-E213Q/D219I (0.2 μM for pThr-containing substrate, 2 μM for pSer-containing substrate) and immediately placed in the plate reader. Initial rates for each substrate concentration were determined by linear fitting. Replicate initial rates were plotted versus substrate concentration and fit to the Michaelis-Menten equation to determine $k_{cat}$ and $K_M$ using non-linear regression in GraphPad Prism 10.

**Western blot detection of kinase activation loops in OspF inducible cell line.** HEK293T OspF Flp-In cells were grown in 150 mm culture plates in the presence or absence of 1 μg/mL doxycycline for 24 hours. For kinase pathway activation, the doxycycline media was removed after 24 hrs and serum free DMEM was supplemented with either 20 ng/mL Epidermal Growth Factor (EGF) (PeproTech) or 100 µM anisomycin (Sigma-Aldrich). After 1 h of incubation with EGF or anisomycin, the media was removed and the cells were incubated for 1 h with only DMEM. Cells were washed with PBS and detached with 0.25% trypsin-EDTA. Detached cells were harvested by centrifugation and washed twice with PBS to remove trypsin-EDTA. Pellets were stored at -80°C until time of western blot. Pellets were resuspended in RIPA lysis buffer with protease and phosphatase inhibitors (50 mM Tris pH 8, 150 mM NaCl, 0.1% SDS, 0.5% sodium deoxycholate, 1% Triton X-100, 1× HALT protease/phosphatase cocktail (Thermo Fisher Scientific), 1 mM PMSF). Resuspended pellets were lysed on ice for 5 minutes and the protein extract was clarified via centrifugation at 10,000 × g at 4°C for 10 min. The concentration of the clarified lysate was determined with a BCA assay (Thermo Fisher Scientific). 20 µg of protein was loaded per well for non-stimulated lysate samples, 25 µg of protein was loaded for EGF and anisomycin samples. Detection of kinase phosphomodiforms and OspF expression were performed with the following primary antibodies and dilutions: 1:2,000 anti-phospho-ERK1/2(Thr202/Tyr204) (Cell Signaling Technology #9106), 1:1,000 anti-phospho-p38 MAPK(Thr180/Tyr182) (Cell Signaling Technology #9215), 1:2,000 anti-phospho-SAPK/JNK(Thr183/Tyr185) (Cell Signaling Technology #9255), 1:1,000-1:5,000 anti-V5 antibody (Invitrogen #46-0705), or 1:20,000 anti-ERK1/2 (Sigma-Aldrich #M5670). Loading control primary antibodies included at

1:1,000 dilution: anti-β-tubulin (rabbit) (Cell Signaling Technology #2146), anti-β-tubulin (mouse) (Cell Signaling Technology #86298) or α-actinin (rabbit) (Cell Signaling Technology #3134). Detection was performed using LiCor secondary antibodies at 1:20,000 dilution: anti-mouse IRDye 680RD (#925-68070), anti-rabbit 680RD (#925-68071), anti-mouse 800CW (#925-32210), or anti-rabbit 800CW (#925-32211). Blots were imaged with an Amersham Typhoon biomolecular imager using the automatic settings for near-infrared detection.

**Table S1. Sequences of primers used in site-directed mutagenesis.**

| Variant | Forward primer sequence | Reverse primer sequence |
|---|---|---|
| H104A | GTTGGGGACAAATTTGCAATTAGCATCGCT CGC | GCGAGCGATGCTAATTGCAAATTTGTCCCCAAC |
| K134A | CCTATTGATAAGTGGGCAATTACGGACATG AAT | ATTCATGTCCGTAATTGCCCACTTATCAATAGG |
| Q143A | ATGAATCGCGTCTCCGCACAATCTCGCGTT GGG | CCCAACGCGAGATTGTGCGGAGACGCGATTCA T |
| Q144A | AATCGCGTCTCCCAAGCATCTCGCGTTGG GATT | AATCCCAACGCGAGATGCTTGGGAGACGCGATT |
| S145A | CGCGTCTCCCAACAAGCACGCGTTGGGAT TGGT | ACCAATCCCAACGCGTGCTTGTTGGGAGACGC G |
| S145X | TGAATCGCGTCTCCCAACAANNKCGCGTT GGGA | TCCCAACGCGMNNTTGTTGGGAGACGCGATTC A |
| R146A | GTCTCCCAACAATCTGCAGTTGGGATTGGT GCT | AGCACCAATCCCAACTGCAGATTGTTGGGAGAC |
| V147A | TCCCAACAATCTCGCGCAGGGATTGGTGC TCAG | CTGAGCACCAATCCCTGCGCGAGATTGTTGGGA |
| Y156A | GCTCAGTTTACTTTGGCAGTAAAGTCCGAC CAA | TTGGTCGGACTTTACTGCCAAAGTAAACTGAGC |
| E213A | GTTTCATATCGTAATGCATTACGCTCAGAT CGT | ACGATCTGAGCGTAATGCATTACGATATGAAAC |
| E213Q | AAGTACGTTTCATATCGTAATCAATTACGC TCAGATCGTGATG | CATCACGATCTGAGCGTAATTGATTACGATATGA AACGTACTT |
| L214A | TCATATCGTAATGAAGCACGCTCAGATCGT GAT | ATCACGATCTGAGCGTGCTTCATTACGATATGA |
| R215A | TATCGTAATGAATTAGCATCAGATCGTGAT GGC | GCCATCACGATCTGATGCTAATTCATTACGATA |
| S216A | CGTAATGAATTACGCGCAGATCGTGATGG CTCC | GGAGCCATCACGATCTGCGCGTAATTCATTACG |
| D217A | AATGAATTACGCTCAGCACGTGATGGCTC CGAA | TTCGGAGCCATCACGTGCTGAGCGTAATTCATT |
| R218A | GAATTACGCTCAGATGCAGATGGCTCCGA ACGT | ACGTTCGGAGCCATCTGCATCTGAGCGTAATTC |
| D219A | TTACGCTCAGATCGTGCAGGCTCCGAACG TCAG | CTGACGTTCGGAGCCTGCACGATCTGAGCGTAA |
| D219X | TTACGCTCAGATCGTNNKGGCTCCGAACG TCAG | CTGACGTTCGGAGCCMNNACGATCTGAGCGTA A |

**Table 2. Sequences of proteins expressed in the study.**

| Protein name | Protein sequence |
|---|---|
| WipA | MPKRLINKNIDIYNYPNEFEDNLGSISLGDLHGNAIKLIHFLFRHKIIKFKTEII<br>NFHEAYQQFVTIYEQYDDMVQEYLEIRTLLQLIQIKITNAQQRILDIEQKLSL<br>ATDHQKEFSQSLLQLKKPIEANLQMAEKSKAGLEEKLSGLKTRLPSCIERF<br>NKFMTQIEINDIKTLIRLLGDEVADRGSCDYFTLRILDFLYQNQIAIKIILSNHG<br>YEFIHAYEKLVVGQPFKPKGYIGDIQIKSFWGLQLLLEQSVITEEELRSLVER<br>AYKPTLKIIDYSLSEDGITLYSHAPIRFDSIRMAASQLGVTYNDSTKEALAETI<br>DQLNAQLQIYMKNNMLHLLFENNEINDPTNMTDEERNASPLIYLVWNRWN<br>ESKEVENARPGKYNGYFVTYVHGHDPFQSPLTYVYNLDTLCGKYSRVGE<br>EEQINKAFQFLTENRHTNVDKTASESLLRNISRYKVLDSDEYTLKHKIPKTS<br>LELATDILDCKIKESLIKLSLLGKPKAVSGSLSVDQNISIPNQASLGKGHHHH<br>HH |
| WipB | MGHHHHHHDYDIPTTENLYFQGSMKTQRIIHPNIDIRKFPEVNTDFSMTDIS<br>MGDLHANALLFLNILVRQGIIAISPENYAKFAEIYTLPELQADYWGTEAPVFS<br>AENKQERLEEIKKQYNALIAQIKIINTKKLIRLIGDELVDRGVIDYFILKLLQAL<br>YDQGADFEILLSNHGIEFVEACELFKENGNKLVAKRLGNIQHGNSFHALQE<br>AIAAGAISNEEVLNIYHQVYKKHLKIISYSLDPDANEIKVFSHAGIGLNHIRGL<br>ARKFKVPYSEESAVDLAKTIDAINKKFAEKASSGEIHTLYTHDMMYRGYAG<br>EHLNSTDEVVAATVWGREYGDLIRTSKKFKITFIHGHDSYDPEKVEHVTLN<br>NQLGQFQNNVGDLYLYATNG |
| OspF | MGHHHHHHDYDIPTTENLYFQGSMPIKKPCLKLNLDSLNVVKSEIPQMLSA<br>NERLKNNFNILYNQIRQYPAYYFKVASNVPTYSDICQFFSVMYQGFQIVNH<br>SGDVFIHACRENPQSKGDFVGDKFHISIAREQVPLAFQILSGLLFSEDSPID<br>KWKITDMNRVSQQSRVGIGAQFTLYVKSDQECSQYSALLLHKIRQFIMCLE<br>SNLLRSKIAPGEYPASDVRPEDWKYVSYRNELRSDRDGSERQEQMLREE<br>PFYRLMIE |
| SpvC | MGHHHHHHDYDIPTTENLYFQGSMPINRPNLNLNIPPLNIVAAYDGAEIPST<br>NKHLKNNFNSLHNQMRKMPVSHFKEALDVPDYSGMRQSGFFAMSQGFQ<br>LNNHGYDVFIHARRESPQSQGKFAGDKFHISVLRDMVPQAFQALSGLLFS<br>EDSPVDKWKVTDMEKVVQQARVSLGAQFTLYIKPDQENSQYSASFLHKT<br>RQFIECLESRLSENGVISGQCPESDVHPENWKYLSYRNELRSGRDGGEM<br>QRQALREEPFYRLMTE |
| HopAI | MGHHHHHHDYDIPTTENLYFQGSMNPIQSRFSSVQELRRSNVDIPALKAN<br>GQLEVDGKRYEIRAADDGTISVLRPEQQSKAKSFFKGASQLIGGSSQRAQI<br>AQALNEKVASARTVLHQSAMTGGRLDTLERGESSSATTAIKPTAKQAAQS<br>TFNSFHEWAKQAEAMRNPSRMDIYKIYKQDAPHSHPMSDEQQEEFLHTL<br>KALNGKNGIEVRTQDHDSVRNKKDRNLDKYIAESPDAKRFFYRIIPKHERR<br>EDKNQGRLTIGVQPQYATQLTRAMATLIGKESAITHGKVIGPACHGQMTDS<br>AVLYINGDVAKAEKLGEKLKQMSGIPLDAFVEHTPLSMQSLSKGLSYAESIL<br>GDTRGHGMSRAEVISDALRMDGMPFLARLKLSLSANGYDPDNPALRNTK |
| OspF$_{25-239}$ | MGHHHHHHDYDIPTTENLYFQGSMLSANERLKNNFNILYNQIRQYPAYYF<br>KVASNVPTYSDICQFFSVMYQGFQIVNHSGDVFIHACRENPQSKGDFVGD<br>KFHISIAREQVPLAFQILSGLLFSEDSPIDKWKITDMNRVSQQSRVGIGAQF<br>TLYVKSDQECSQYSALLLHKIRQFIMCLESNLLRSKIAPGEYPASDVRPED<br>WKYVSYRNELRSDRDGSERQEQMLREEPFYRLMIE |

**Table S3.** List of ProteomeXchange accession numbers and experimental information.

| **Experiment #** | **experiment description** | **Relevant to** | **Supplementary Dataset #** | **ProteomeXchange #** |
|---|---|---|---|---|
| Exp1 | Phosphoproteome-derived peptide libraries from untreated HEK293T cells | Fig. 1 | 1 | PXD067199 |
| Exp2 | Phosphoproteome-derived peptide libraries from pervanadate-treated HEK293T cells | Fig. 1 | 2 | PXD067199 |
| Exp3 | Phosphoproteome-derived peptide libraries from pervanadate-treated K562 cells | Fig. 1 | 4 | PXD067199 |
| Exp4 | Phosphoproteome-derived peptide libraries from pervanadate-treated Jurkat cells | Fig. 1 | 3 | PXD067199 |
| Exp5 | Timecourse of λPP treatment of Phospropel from pervanadate-treated HEK293T cells | Fig. 1 | 6 | PXD067199 |
| Exp6 | Timecourse of PTP1B$_{1\text{-}321}$ treatment of Phospropel from pervanadate-treated HEK293T cells | Fig. 2 | **7** | PXD067202 |
| Exp7 | Timecourse of PP2Ac treatment of Phospropel from pervanadate-treated HEK293T cells | Fig. 2 | 8 | PXD067202 |
| Exp8 | Timecourse of WipA treatment of Phospropel from pervanadate-treated HEK293T cells | Fig. 3 | 9 | PXD067205 |
| Exp9 | Timecourse of WipB treatment of Phospropel from pervanadate-treated HEK293T cells | Fig. 3 | 10 | PXD067205 |
| Exp10 | OspF treatment of Phospropel from pervanadate-treated HEK293T cells | Fig. 4 | 11 | PXD067206 |
| Exp11 | SpvC treatment of Phospropel from pervanadate-treated HEK293T cells | Fig. 4 | 12 | PXD040046 |
| Exp12 | HopAI treatment of Phospropel from pervanadate-treated HEK293T cells | Fig. 4 | 12 | PXD040046 |
| Exp13 | OspF$_{25\text{-}239}$ treatment of Phospropel from pervanadate-treated HEK293T cells | Fig. 4 | 13 | PXD040046 |
| Exp14 | Inducible OspF TMT experiment | Fig. 4 | 14 | PXD067267 |
| Exp15 | Treatment of phosphoproteome-derived peptide libraries with OspF Ala variants | Fig. 5 | 15 | PXD067209 |

| | | | | |
|---|---|---|---|---|
| Exp 16 | Treatment of phosphoproteome-derived peptide libraries with additional OspF variants | Fig. 5 | 16 | PXD067209 |
| Exp 17 | Phosphoproteome-derived peptide libraries generated from pervanadate-treated HEK293T cells and digested with GluC. | Fig. 1 | 17 | PXD074748 |
| Exp 18 | Phosphoproteome-derived peptide libraries generated from pervanadate-treated HEK293T cells and digested with LysC. | Fig. 1 | 18 | PXD074748 |
| Exp 19 | Phosphoproteome-derived peptide libraries generated from pervanadate-treated HEK293T cells and digested with Chymotrypsin. | Fig. 1 | 19 | PXD074748 |
| Exp 20 | Three hour reactions of lambda phosphatase treatment of phosphoproteome-derived peptide libraries made from pervanadate-treated HEK293T cells and digested with GluC. | Fig. 1 | 20 | PXD074909 |
| Exp 21 | Three hour reactions of PP2Ac treatment of phosphoproteome-derived peptide libraries made from pervanadate-treated HEK293T cells and digested with GluC. | Fig. 2 | 21 | PXD074909 |
| Exp 22 | One hour reactions of PTP1B treatment of phosphoproteome-derived peptide libraries made from pervanadate-treated HEK293T cells and digested with GluC. | Fig. 2 | 22 | PXD074953 |
| Exp 23 | Two hour reactions of WipA treatment of phosphoproteome-derived peptide libraries made from pervanadate-treated HEK293T cells and digested with GluC. | Fig. 3 | 23 | PXD074951 |
| Exp 24 | Two hour reactions of WipB treatment of phosphoproteome-derived peptide libraries made from pervanadate-treated HEK293T cells and digested with GluC. | Fig. 3 | 24 | PXD074951 |
| Exp 25 | Twenty-four hour reactions of OspF | Fig. 4 | 25 | PXD075092 |

| | | | | |
|---|---|---|---|---|
| | treatment of phosphoproteome-derived peptide libraries made from pervanadate-treated HEK293T cells and digested with GluC. | | | |
| Exp 26 | Twenty-four hour reactions of OspFdN treatment of phosphoproteome-derived peptide libraries made from pervanadate-treated HEK293T cells and digested with GluC. | Fig. 4 | 26 | PXD075092 |
| Exp 27 | Three hour reactions of lambda phosphatase treatment of phosphoproteome-derived peptide libraries made from pervanadate-treated HEK293T cells and digested with LysC. | Fig. 1 | 27 | PXD074909 |
| Exp 28 | Three hour reactions of PP2Ac treatment of phosphoproteome-derived peptide libraries made from pervanadate-treated HEK293T cells and digested with LysC. | Fig. 2 | 28 | PXD074909 |
| Exp 29 | One hour reactions of PTP1B treatment of phosphoproteome-derived peptide libraries made from pervanadate-treated HEK293T cells and digested with LysC. | Fig. 2 | 29 | PXD074953 |
| Exp 30 | Two hour reactions of WipA treatment of phosphoproteome-derived peptide libraries made from pervanadate-treated HEK293T cells and digested with LysC. | Fig. 3 | 30 | PXD074951 |
| Exp 31 | Two hour reactions of WipB treatment of phosphoproteome-derived peptide libraries made from pervanadate-treated HEK293T cells and digested with LysC. | Fig. 3 | 31 | PXD074951 |
| Exp 32 | Twenty-four hour reactions of OspF treatment of phosphoproteome-derived peptide libraries made from pervanadate-treated HEK293T cells and digested with LysC. | Fig. 4 | 32 | PXD075092 |
| Exp 33 | Twenty-four hour reactions of OspFdN treatment of phosphoproteome- | Fig. 4 | 33 | PXD075092 |

| | | | | |
|---|---|---|---|---|
| | derived peptide libraries made from pervanadate-treated HEK293T cells and digested with LysC. | | | |
| Exp 34 | Twenty-four hour reactions of OspF treatment of phosphoproteome-derived peptide libraries made from pervanadate-treated HEK293T cells and digested with Chymotrypsin. | Fig. 4 | 34 | PXD075092 |
| Exp 35 | Twenty-four hour reactions of OspFdN treatment of phosphoproteome-derived peptide libraries made from pervanadate-treated HEK293T cells and digested with Chymotrypsin. | Fig. 4 | 35 | PXD075092 |
| Exp 36 | Three hour reactions of PP2Ac treatment of phosphoproteome-derived peptide libraries made from pervanadate-treated HEK293T cells and digested with trypsin in the presence of various concentrations of okadaic acid. | Fig. 2 | 36 | PXD074779 |
| Exp 37 | One hour reactions of PTP1B treatment of phosphoproteome-derived peptide libraries made from pervanadate-treated HEK293T cells and digested with trypsin in the presence of various concentrations of okadaic acid. | Fig. 2 | 37 | PXD074779 |
| Exp 38 | Two hour reactions of WipA treatment of phosphoproteome-derived peptide libraries made from pervanadate-treated HEK293T cells and digested with trypsin in the presence of various concentrations of okadaic acid. | Fig. 3 | 38 | PXD074779 |
| Exp 39 | Two hour reactions of WipB treatment of phosphoproteome-derived peptide libraries made from pervanadate-treated HEK293T cells and digested with trypsin in the presence of various concentrations of okadaic acid. | Fig. 3 | 39 | PXD074779 |

| | | | | |
|---|---|---|---|---|
| Exp 40 | Phosphoproteome-derived peptide libraries generated from pervanadate-treated HEK293T cells, digested with trypsin, and enriched using the okadai Select™ Phosphopeptide Enrichment Kits from Thermo Scientific. | Fig. 1 | 40 | PXD074774 |
| Exp 41 | Label free quantification of phosphoproteome of HEK293T cells induced for expression of OspF vs non-induced. | Fig. 4 | 41 | PXD074791 |
| Exp 42 | Label free quantification of phosphoproteome of HEK293T cells induced for expression of OspF vs non-induced and treated with EGF. | Fig. 4 | 42 | PXD074791 |

**Figure S1. Distribution of ptmRS site localization scores in PhosPropels from untreated HEK293T cells.** Scores were computed using the IMP-ptmRS node in Proteome Discoverer 2.4 (ThermoFisher Scientific). Only sites with scores >90 were included in further analysis.

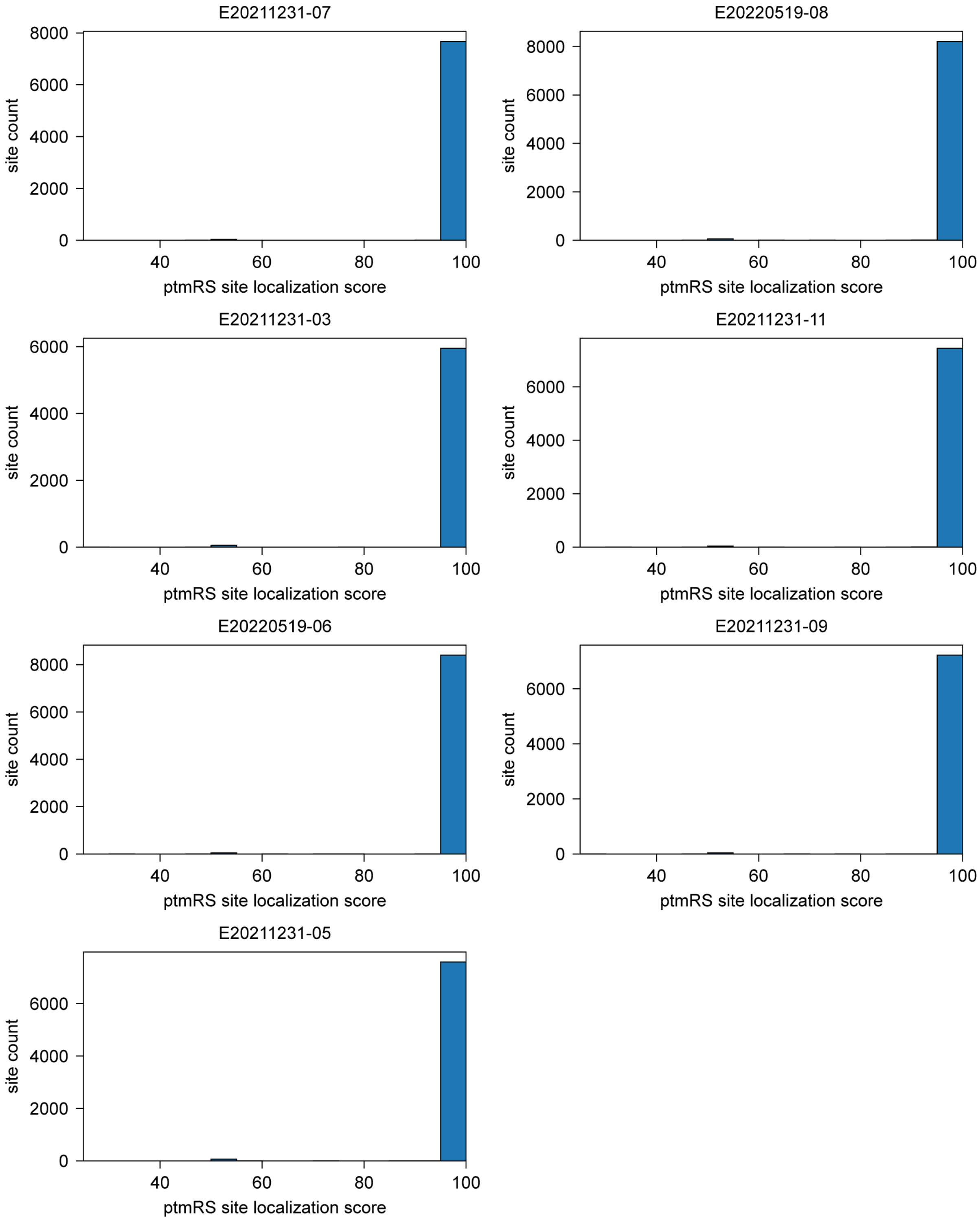

**Figure S2. Distribution of ptmRS site localization scores in PhosPropels from pervanadate-treated HEK293T cells.** Scores were computed using the IMP-ptmRS node in Proteome Discoverer 2.4 (ThermoFisher Scientific). Only sites with scores >90 were included in the analysis.

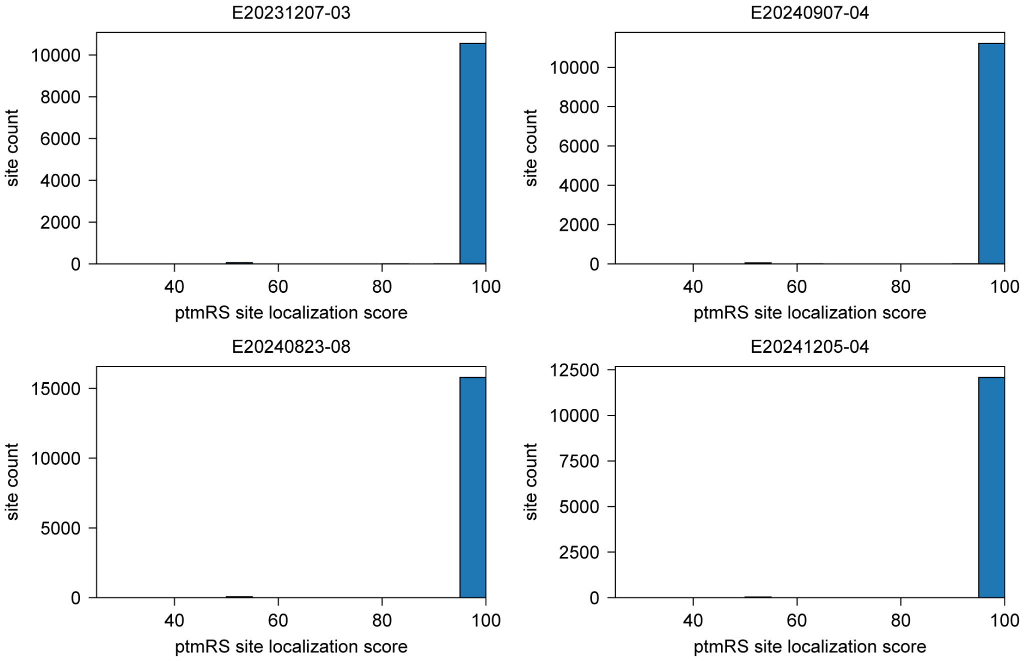

**Figure S3. Phosphoproteome-derived peptide libraries (PhosPropels) from pervanadate-treated Jurkat and K562 cells.** a) Distribution of pSer, pThr, and pTyr sites in PhosPropels from pervanadate-treated HEK293T, Jurkat, and K562 cells. b) Z-score heatmap showing the distribution of all 20 proteinogenic amino acids, pSer, pThr, and pTyr in positions flanking phosphsites in the Jurakt PhosPropel. c) Z-score heatmap showing the distribution of all 20 proteinogenic amino acids, pSer, pThr, and pTyr in positions flanking phosphsites in the Jurakt PhosPropel. d,e) Distrubution of high-scoring kinase PSSM scores among phosphopeptides in the Jurkat (d) and K562 (e) PhosPropels. Node size represents the average $\log_2$ count of sequences that score at least 2 standard deviations above the kinase-specific PSSM mean.

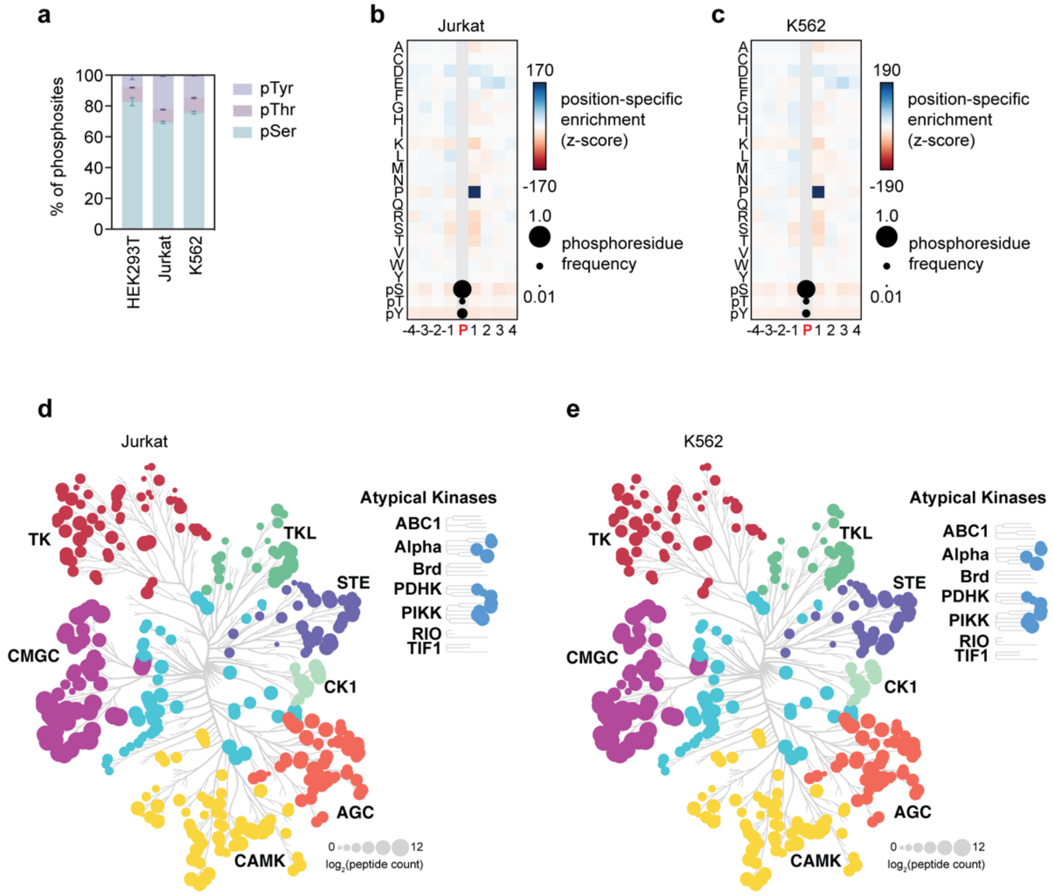

**Figure S4. Distribution of ptmRS site localization scores in PhosPropels from pervanadate-treated Jurkat cells.** Scores were computed using the IMP-ptmRS node in Proteome Discoverer 2.4 (ThermoFisher Scientific). Only sites with scores >90 were included in the analysis.

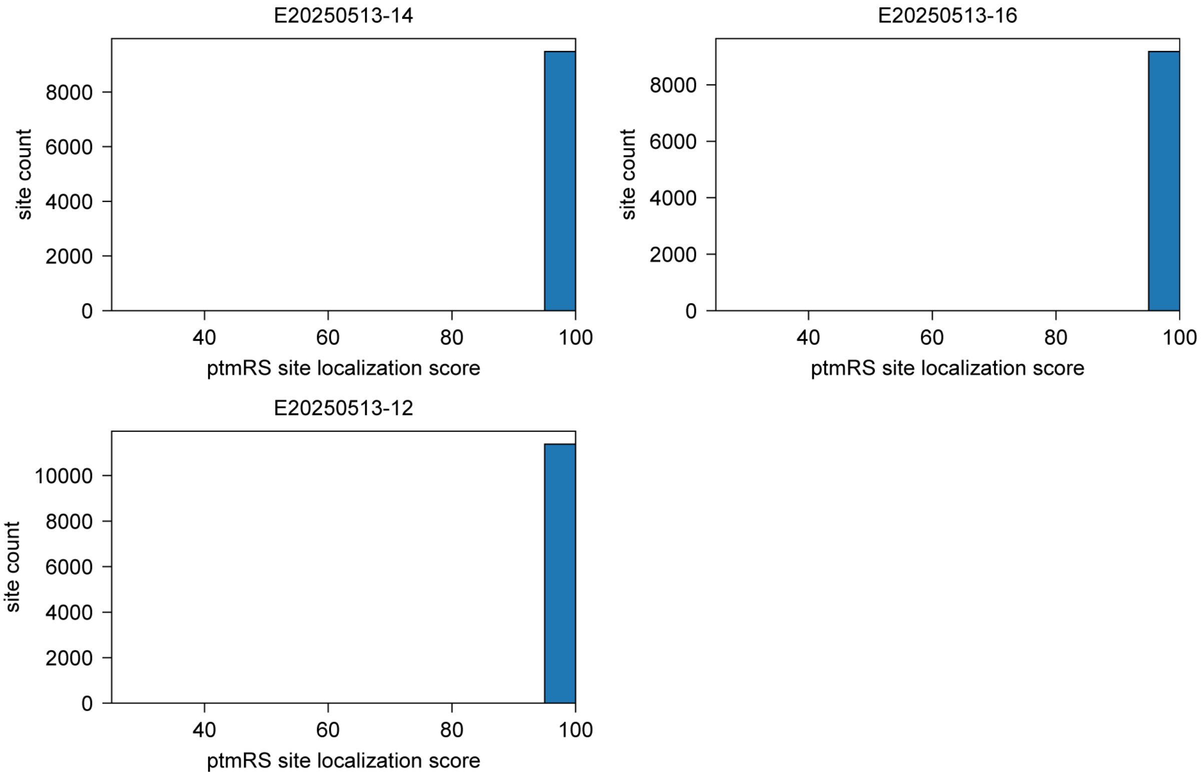

**Figure S5. Distribution of ptmRS site localization scores in PhosPropels from pervanadate-treated K562 cells.** Scores were computed using the IMP-ptmRS node in Proteome Discoverer 2.4 (ThermoFisher Scientific). Only sites with scores >90 were included in the analysis.

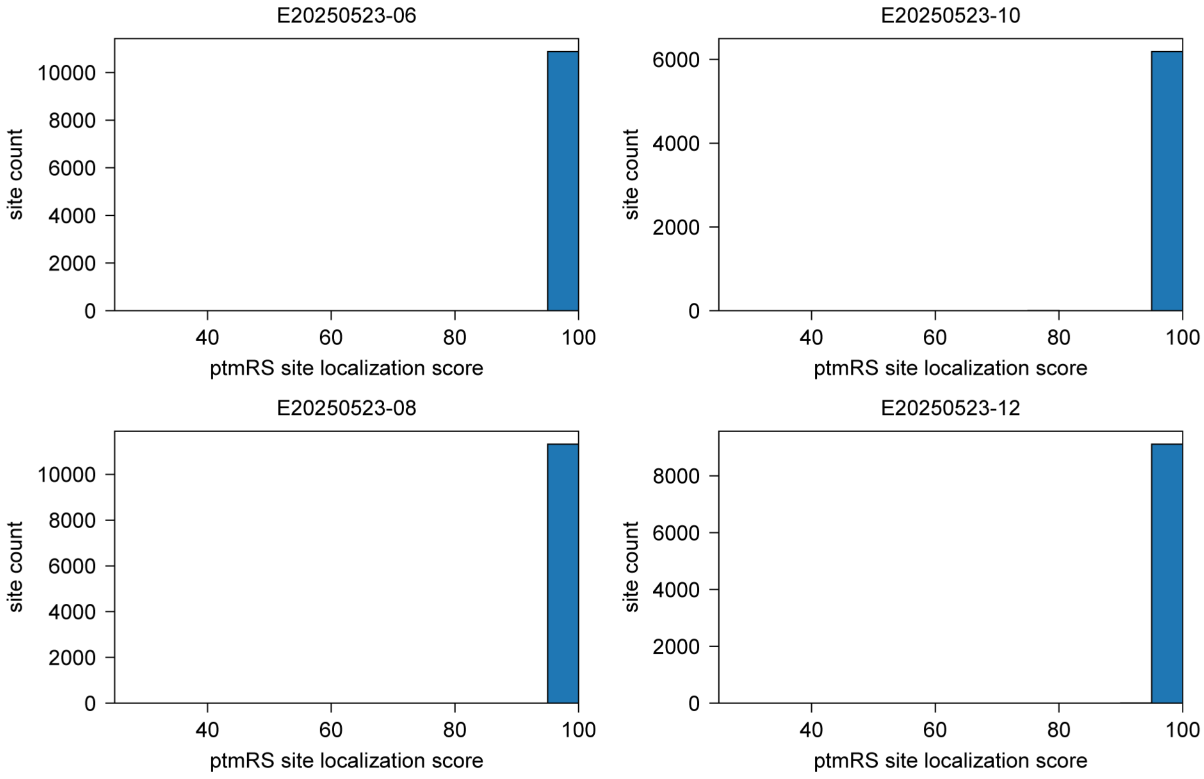

**Figure S6. Phosphoproteome-derived peptide libraries (PhosPropels) analyzed by phosphosite type.** Z-score heatmaps show the distribution of all 20 proteinogenic amino acids, pSer, pThr, and pTyr in positions flanking pSer, pThr, and pTyr sites in PhosPropels. a) Tryptic HEK293T pervanadate library (Left, pSer; middle, pThr; right, pTyr). b) Tryptic Jurkat pervanadate library (Left, pSer; middle, pThr; right, pTyr). c) Tryptic K562 pervanadate library (Left, pSer; middle, pThr; right, pTyr).

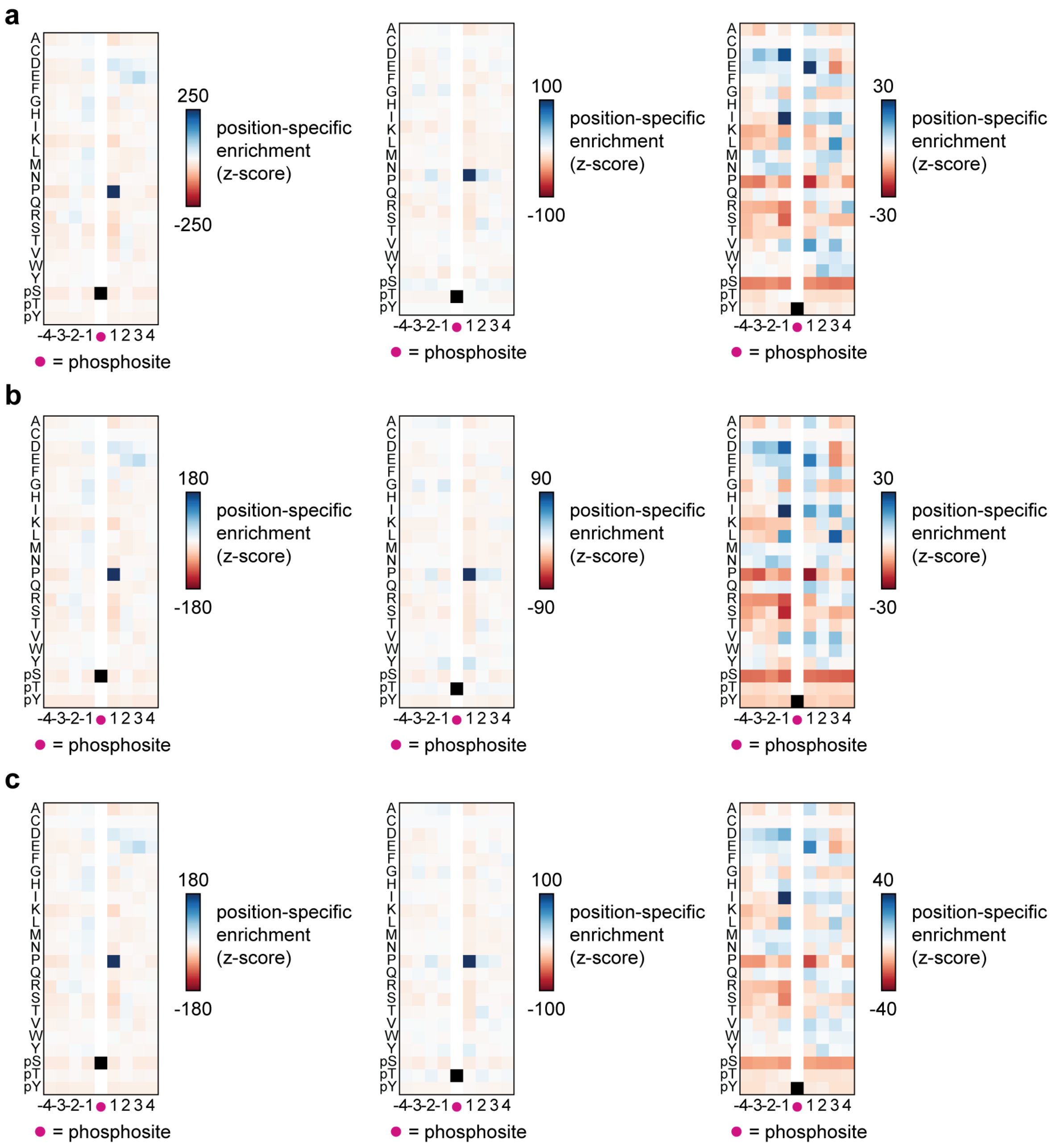

**Figure S7. Phosphoproteome-derived peptide libraries (PhosPropels) enriched from pervanadate-treated HEK293T cells with Fe-NTA.** a) Number of phosphopeptides measured across four replicate preparations of Fe-NTA library. b) Distribution of pSer, pThr, and pTyr sites in PhosPropels from Fe-NTA library. c) Z-score heatmap showing the distribution of all 20 proteinogenic amino acids, pSer, pThr, and pTyr in positions flanking phosphsites in the Fe-NTA PhosPropel.

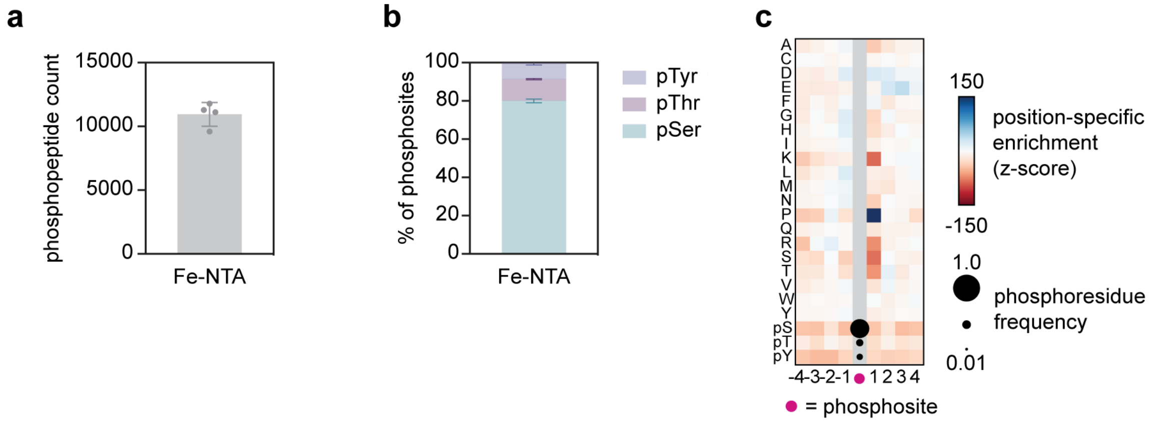

**Figure S8. Representation of proteinogenic amino acids in positions flanking a central phosphosite in tryptic PhosPropels from untreated HEK293T cells.** The purple bars show library abundance and the grey bar shows proteome abundance of each amino acid.

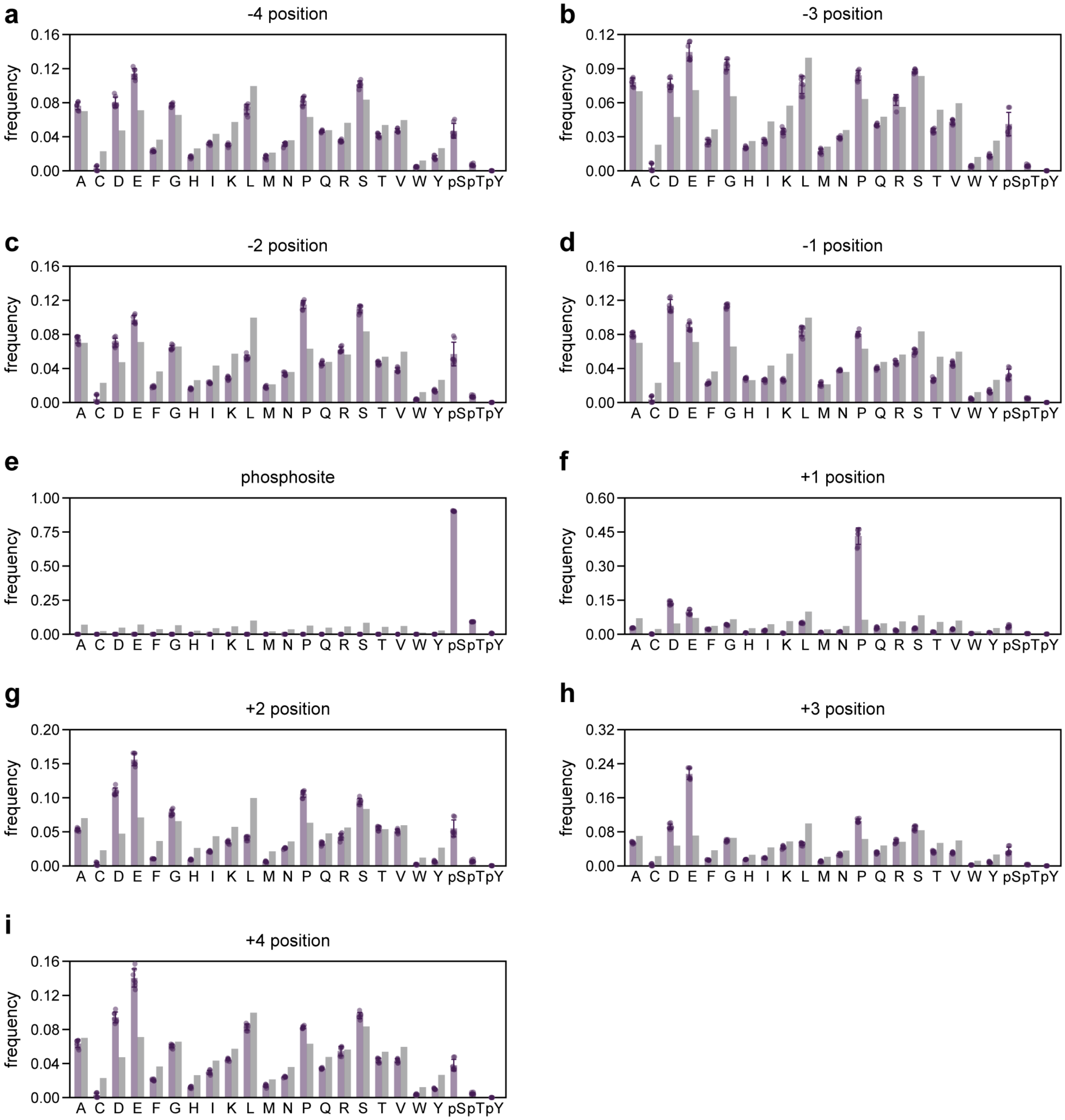

**Figure S9. Representation of proteinogenic amino acids in positions flanking a central phosphosite in tryptic PhosPropels from pervanadate-treated HEK293T cells.** The purple bars show library abundance and the grey bar shows proteome abundance of each amino acid.

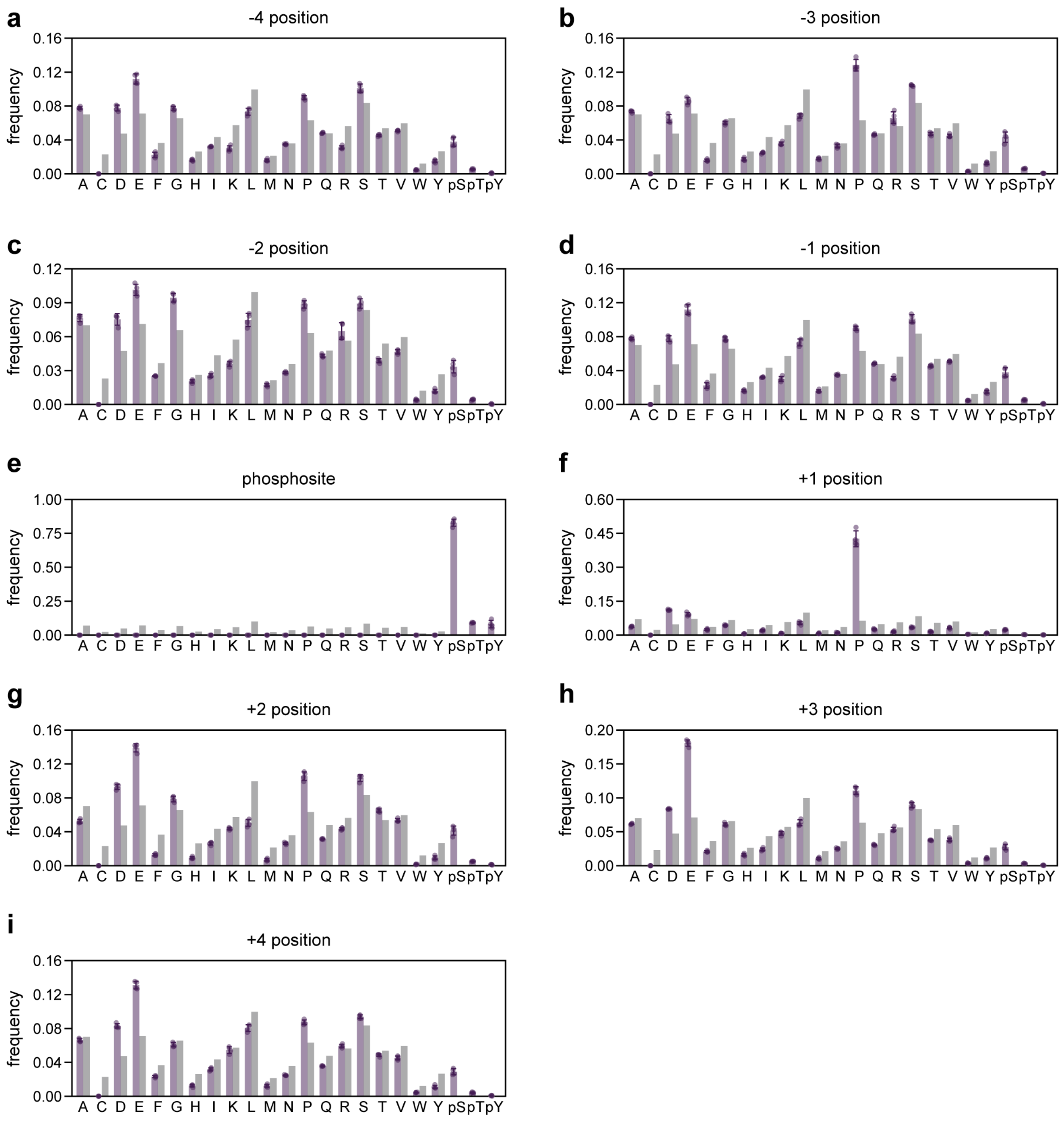

**Figure S10. Representation of proteinogenic amino acids in positions flanking a central phosphosite in GluC PhosPropels from pervanadate-treated HEK293T cells.** The purple bars show library abundance and the grey bar shows proteome abundance of each amino acid.

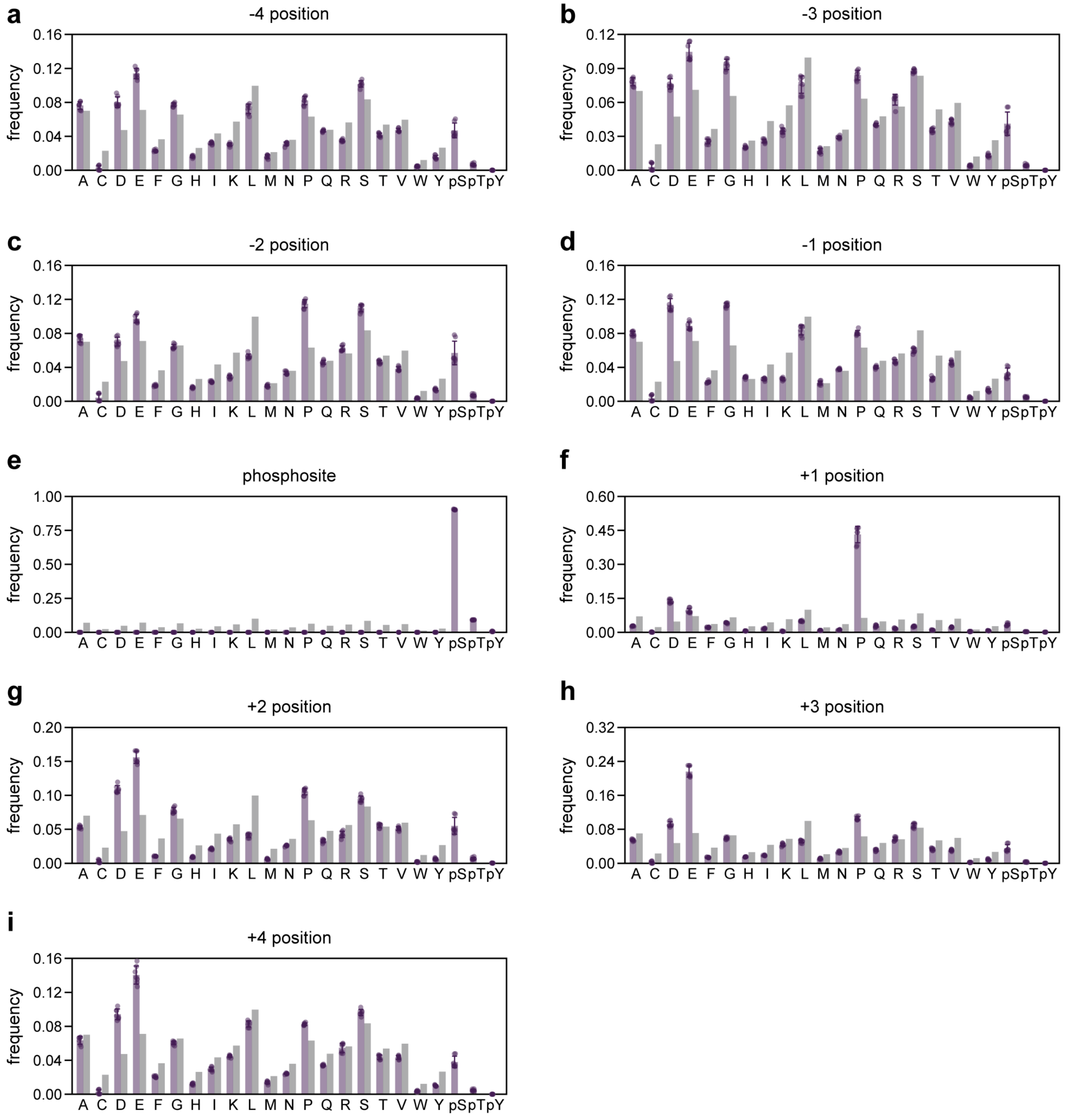

**Figure S11. Representation of proteinogenic amino acids in positions flanking a central phosphosite in LysC PhosPropels from pervanadate-treated HEK293T cells.** The purple bars show library abundance and the grey bar shows proteome abundance of each amino acid.

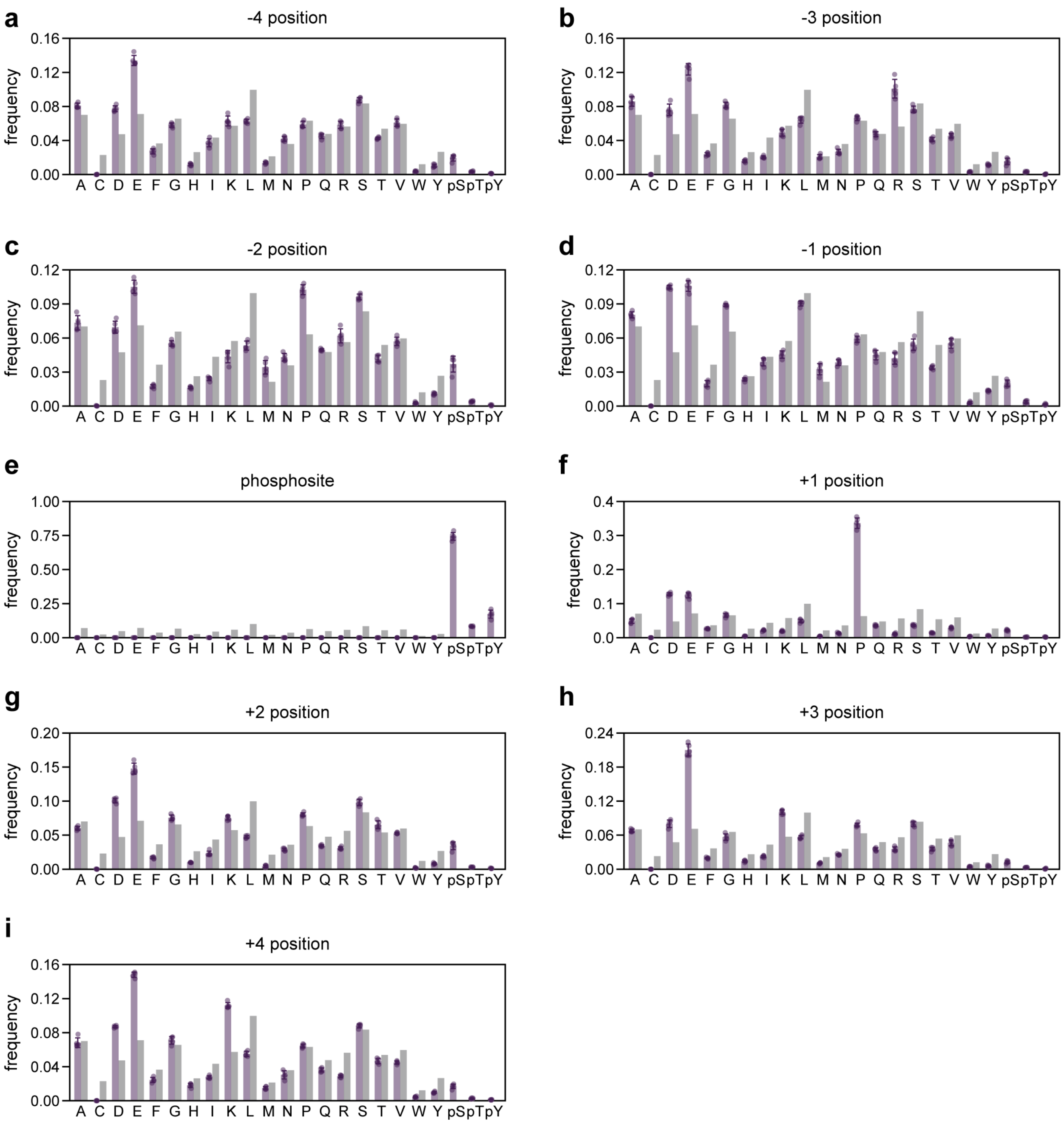

**Figure S12. Representation of proteinogenic amino acids in positions flanking a central phosphosite in tryptic PhosPropels from pervanadate-treated HEK293T cells enriched using Fe-NTA.** The purple bars show library abundance and the grey bar shows proteome abundance of each amino acid.

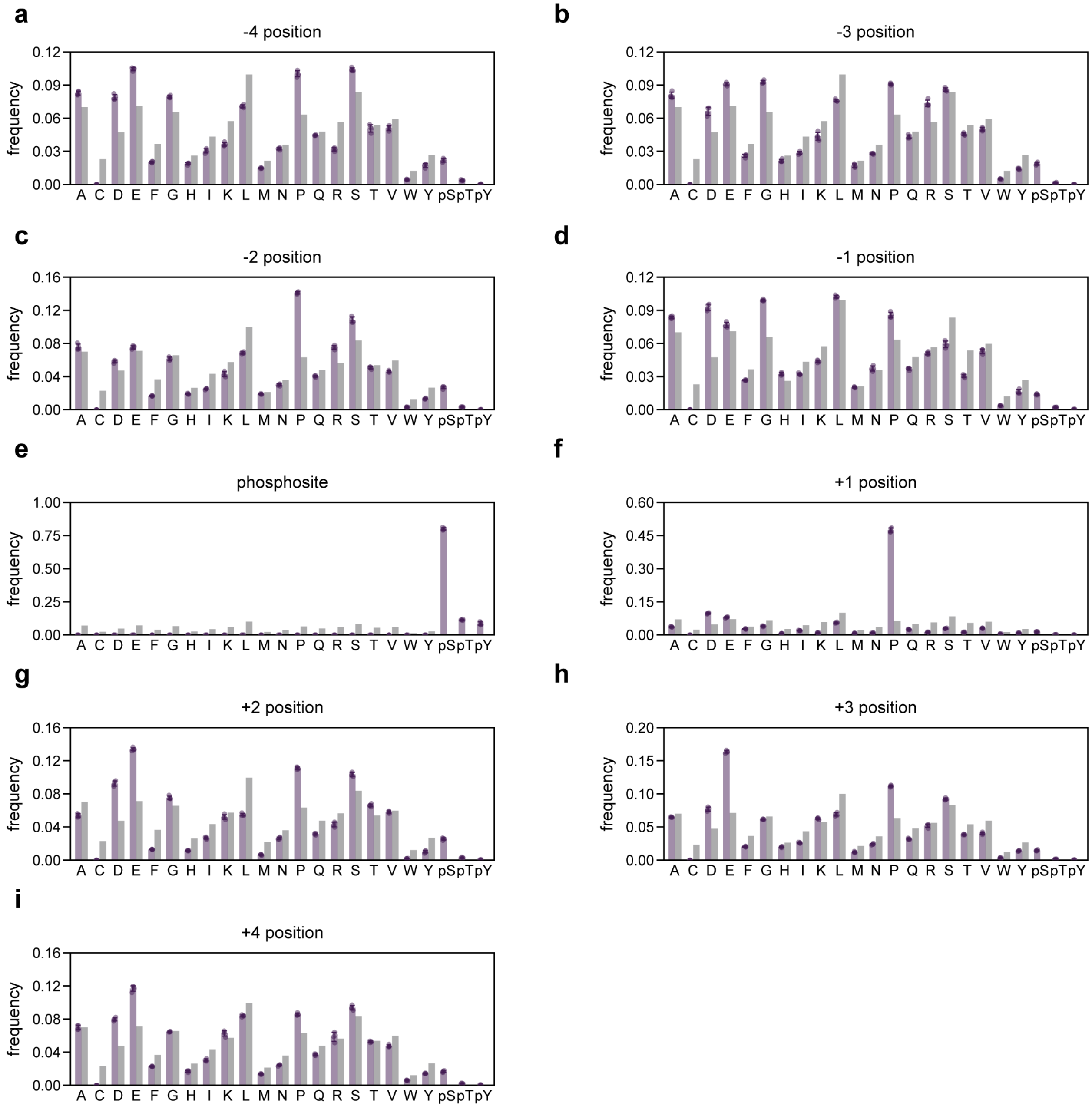

**Figure S13. Representation of Lys and Arg residues in positions flanking a central phosphosite.** Bars show mean ± s.d for the specified library.

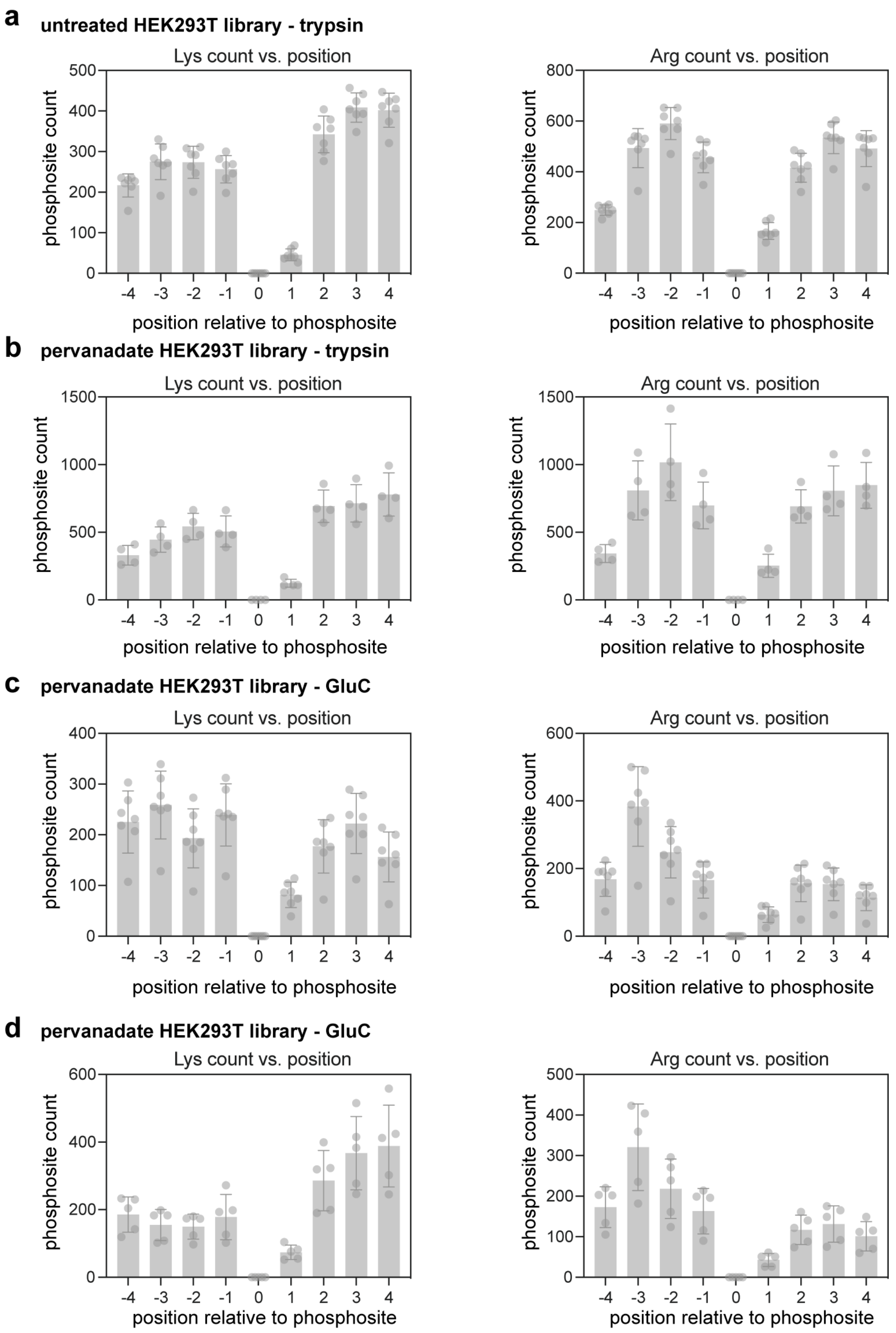

**Figure S14. Representation of pSer, pThr, and pTyr residues in positions flanking a central phosphosite.** Bars show mean ± s.d for untreated (grey) and pervanadate-treated (purple) PhosPropels from HEK293T cells.

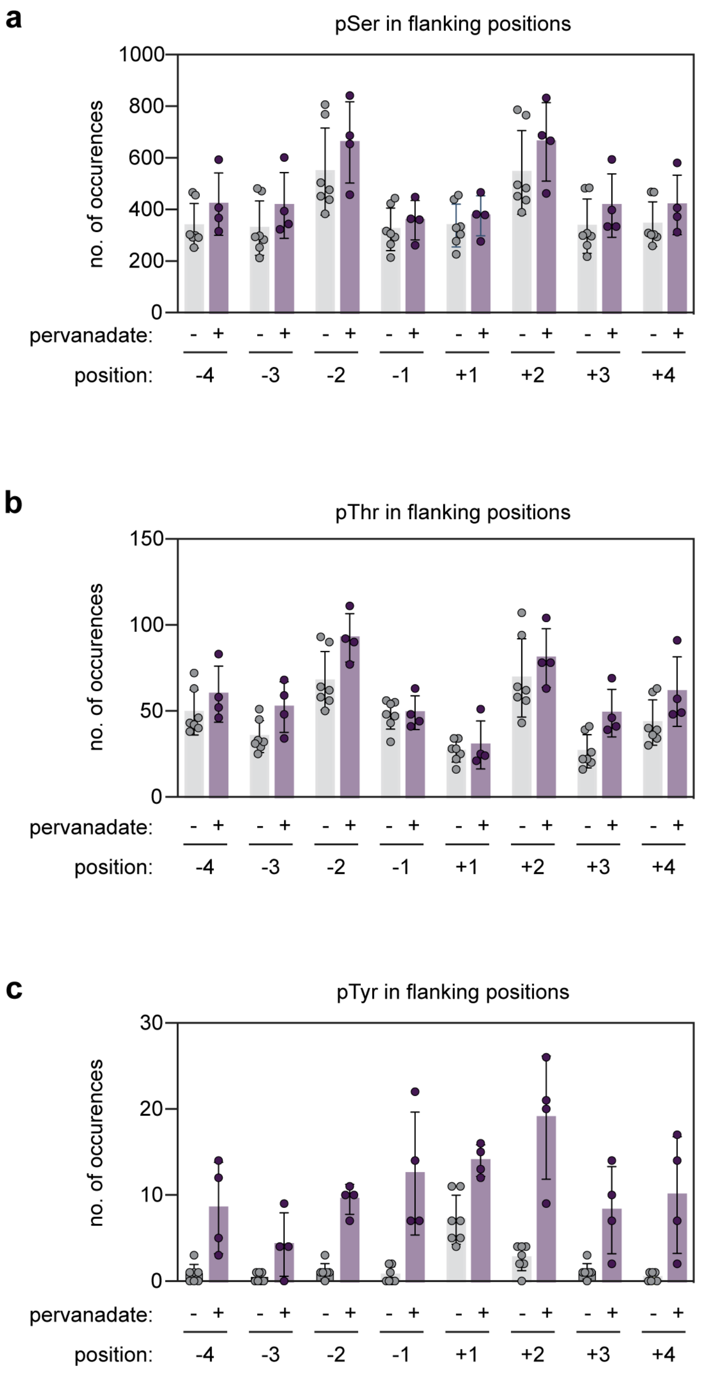

**Figure S15. Peptide length distribution in PhosPropels generated with different proteases.** Histograms were generated from replicate PhosPropel datasets. Total peptide count (N), mean (μ) and standard deviation (σ) are shown at top right.

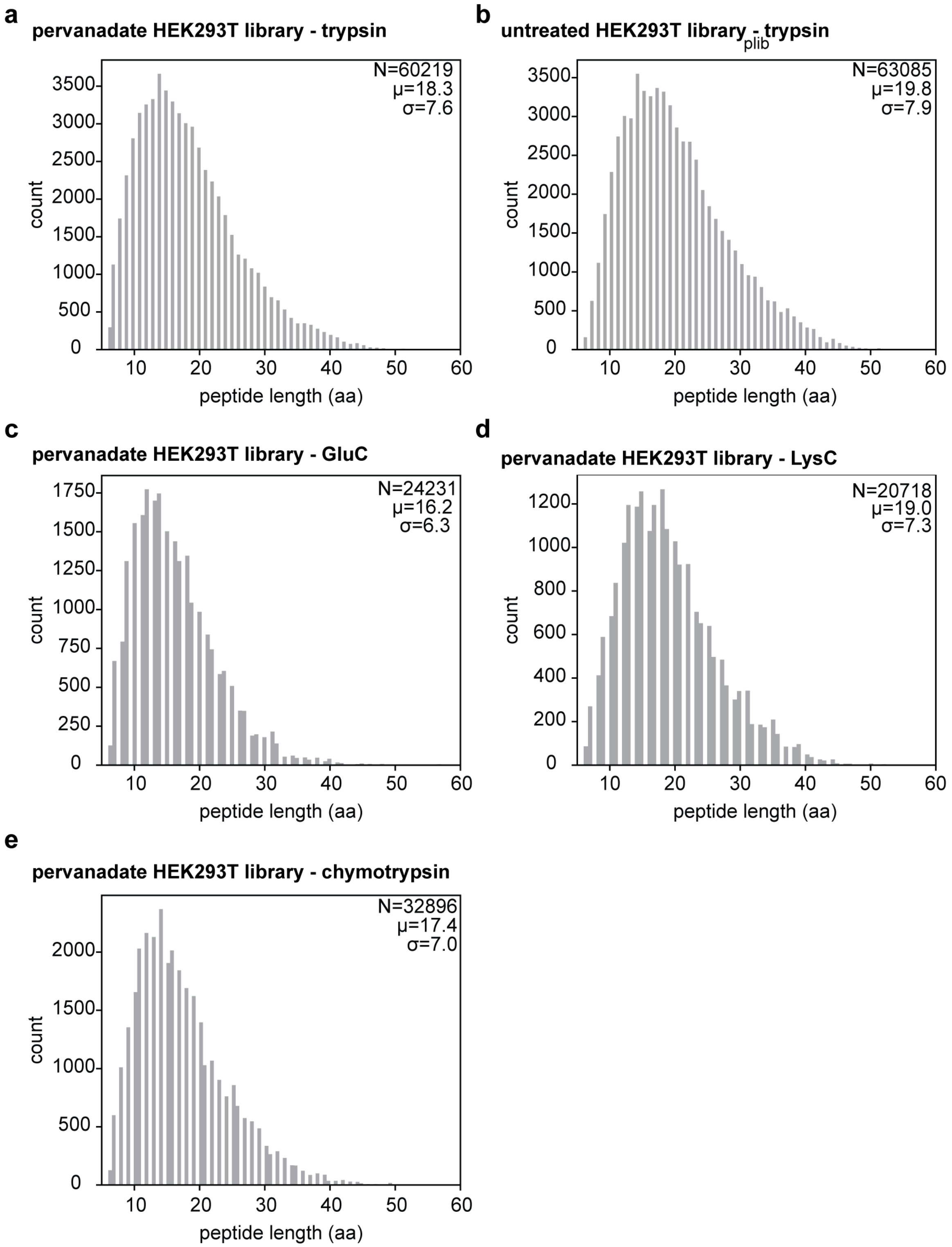

**Figure S16. Distribution of phosphosite distances from peptide N termini in PhosPropels generated with different proteases.** Histograms were generated from replicate PhosPropel datasets. Total peptide count (N), mean (μ) and standard deviation (σ) are shown at top right.

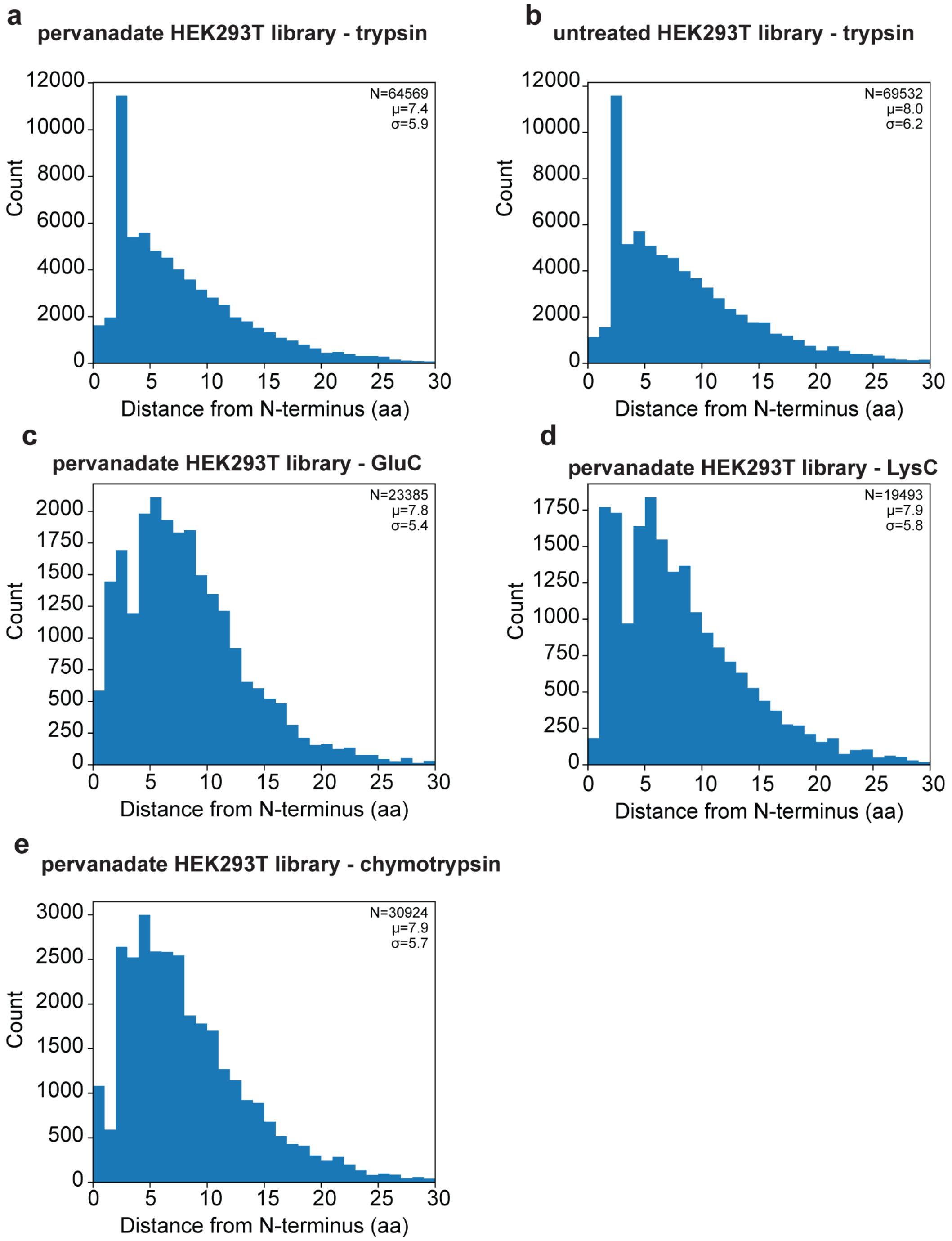

**Figure S17. Distribution of phosphosite distances from peptide C termini in PhosPropels generated with different proteases.** Histograms were generated from replicate PhosPropel datasets. Total peptide count (N), mean (μ) and standard deviation (σ) are shown at top right.

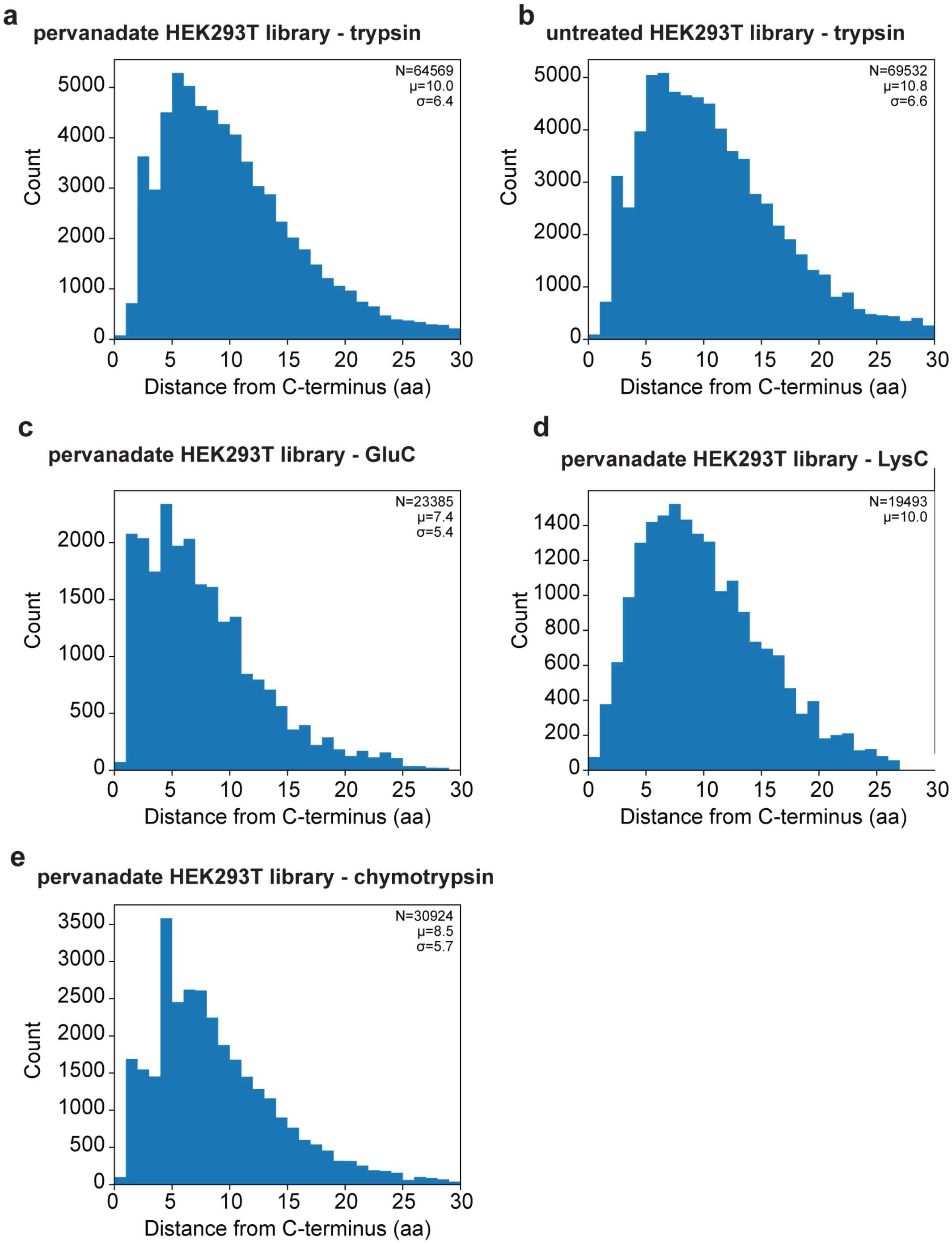

**Figure S18. Distribution of phosphosite minimum distances from peptide N or C termini in PhosPropels generated with different proteases.** Histograms were generated from replicate PhosPropel datasets. Total peptide count (N), mean (μ) and standard deviation (σ) are shown at top right.

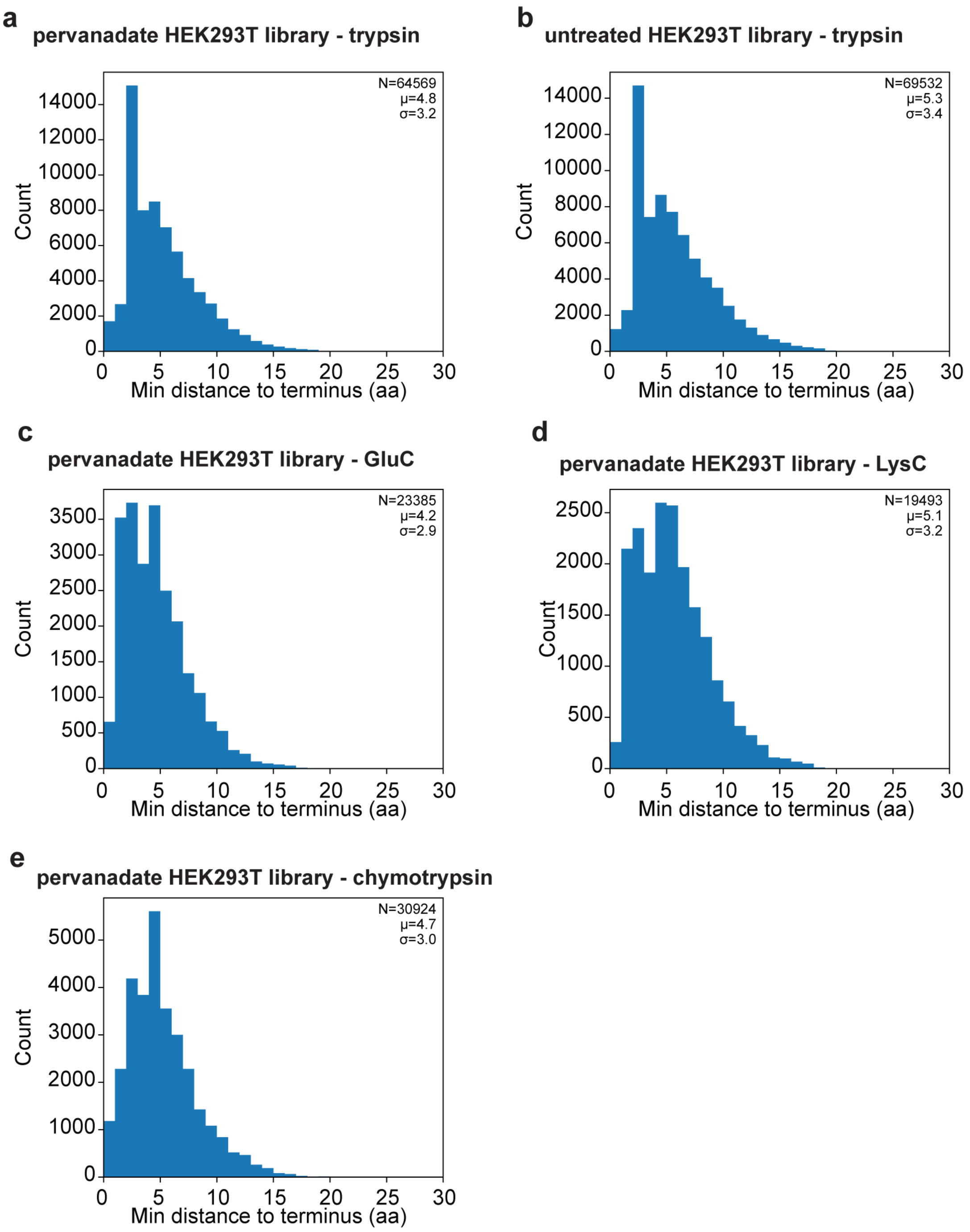

**Figure S19. λ phosphatase specificity profiling with non-tryptic PhosPropels.** a) Heatmap for specificity profiling of λ phosphatase at 180 min using GluC library generated from pervanadate-treated HEK293T cells. b) Heatmap for specificity profiling of λ phosphatase at 180 min using LysC library generated from pervanadate-treated HEK293T cells. Z-scores were calculated by comparing positional frequencies to the 0 min timepoint using counts summed across n = 3 biological replicates. Residue-position combinations with Benjamini-Hochberg FDR-adjusted p-values < 0.0001 were considered significant and are outlined in black.

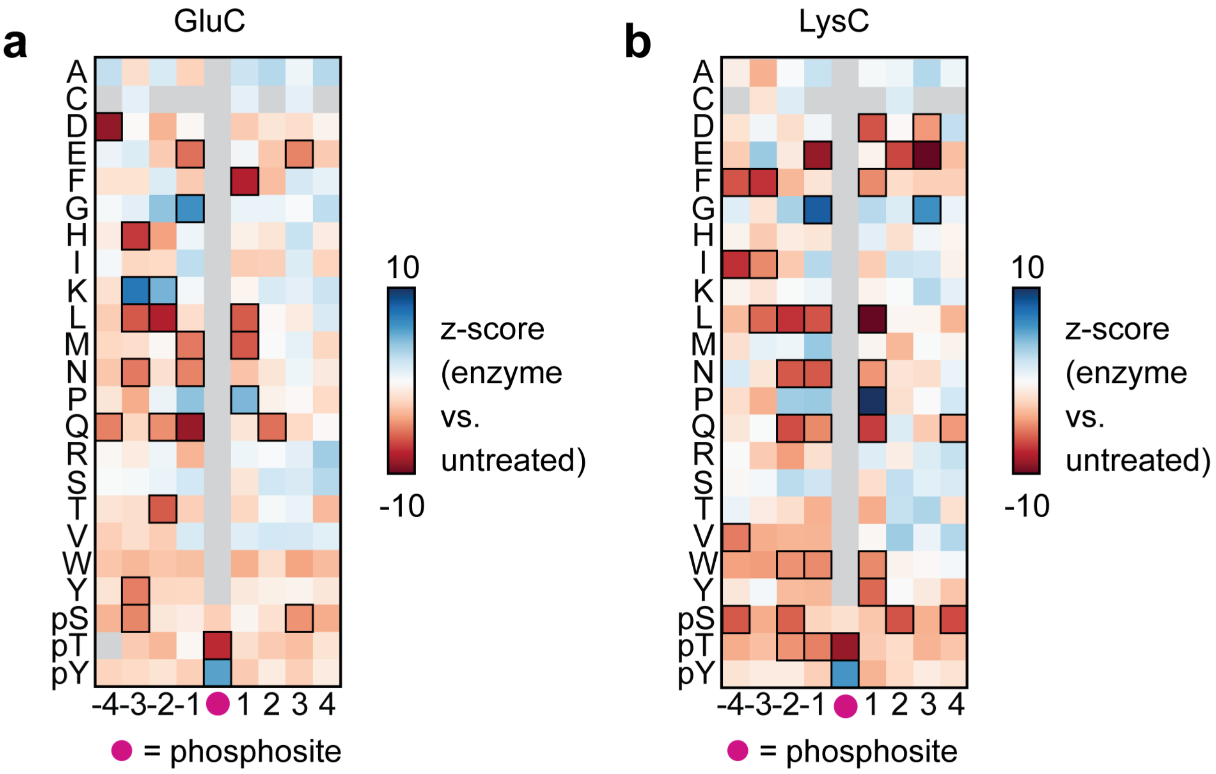

**Figure S20. Comparison of λ phosphatase specificity profiling experiments performed with PhosPropels generated with different proteases.** All experiments used PhosPropels generated from pervanadate-treated HEK293T cells. Dotted lines indicate z = ±4 (corresponding to α ≈ 0.0001). Points are colored according to residue identity (see legend). Points with |z| ≥ 4 of the same sign (upper right and lower left regions of the plot) represent residue-position combinations that were enriched or depleted consistently in both libraries. Points near the origin represent residue-position combinations with little or no enrichment or depletion in either dataset, whereas points exceeding the threshold in only one dataset indicate features enriched or depleted in one library but not the other. Residue-position combinations with large, opposing z-scores (|z| ≥ 4 in both datasets but with opposite sign) appear in the upper left and lower right regions of the plot and indicate protease-dependent differences in inferred specificity. These features are circled in red and labeled. a) Scatterplot comparing z scores obtained using trypsin PhosPropels and LysC PhosPropels. b) Scatterplot comparing z scores obtained using trypsin PhosPropels and GluC PhosPropels. c) Scatterplot comparing z scores obtained using GluC PhosPropels and LysC PhosPropels.

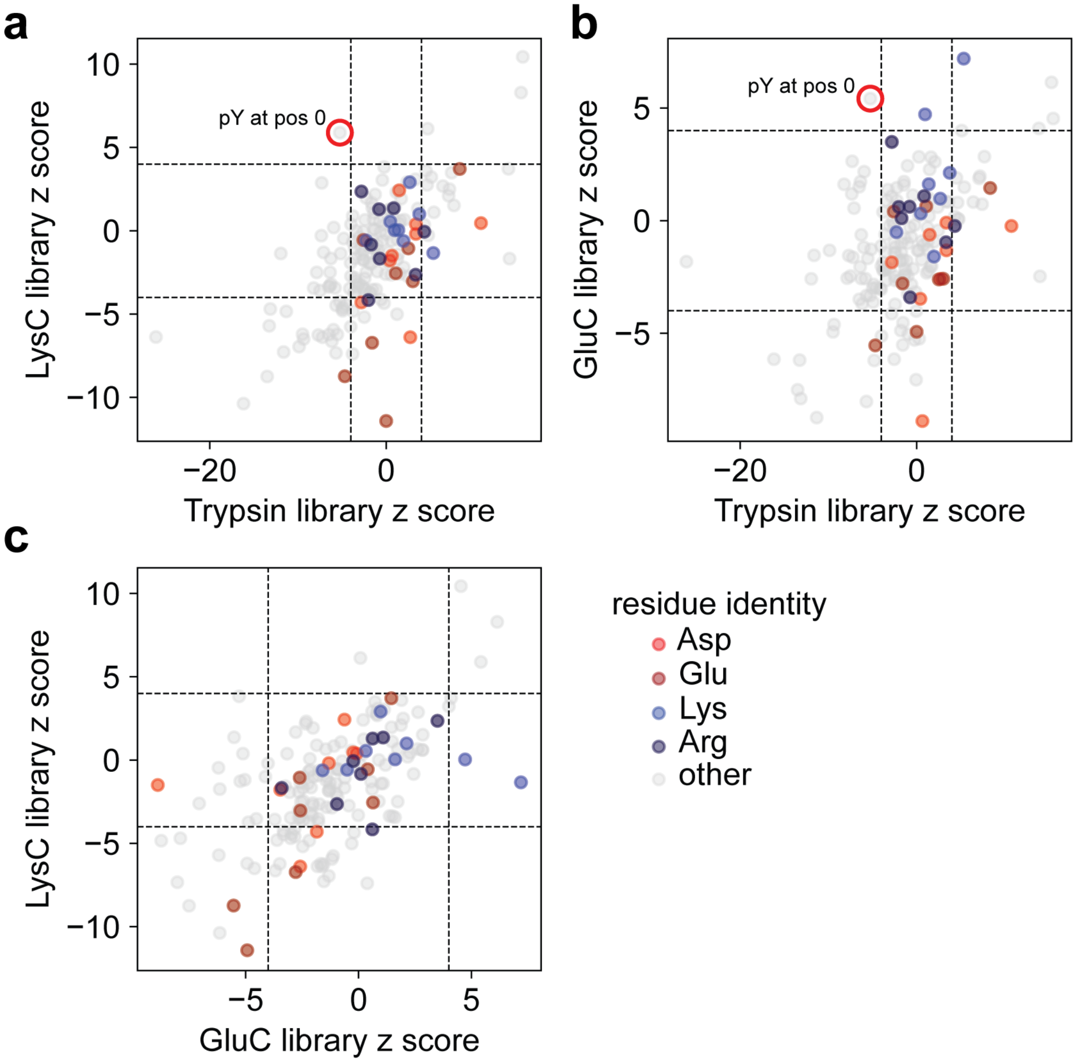

**Figure S21. PTP1B$_{1-321}$ heatmap timecourses.** a) Timecourse in which all pSer, pThr, and pTyr sites were analyzed. b) Timecourse in which data were filtered to analyze only pSer and pThr sites. No enrichment or depletion in flanking residues is detected because pSer and pThr are not substrates of PTP1B$_{1-321}$. Z-scores were calculated by comparing positional frequencies to the 0 min timepoint using counts summed across n = 3 biological replicates after filtering for the indicated phosphosite type. Residue-position combinations with Benjamini-Hochberg FDR-adjusted p-values < 0.0001 were considered significant and are outlined in black.

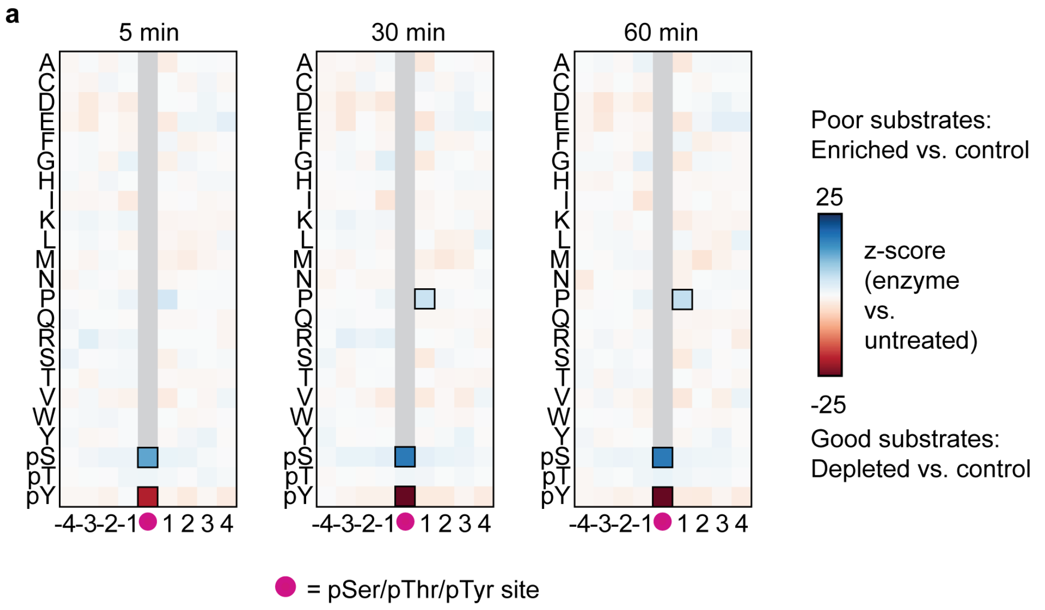


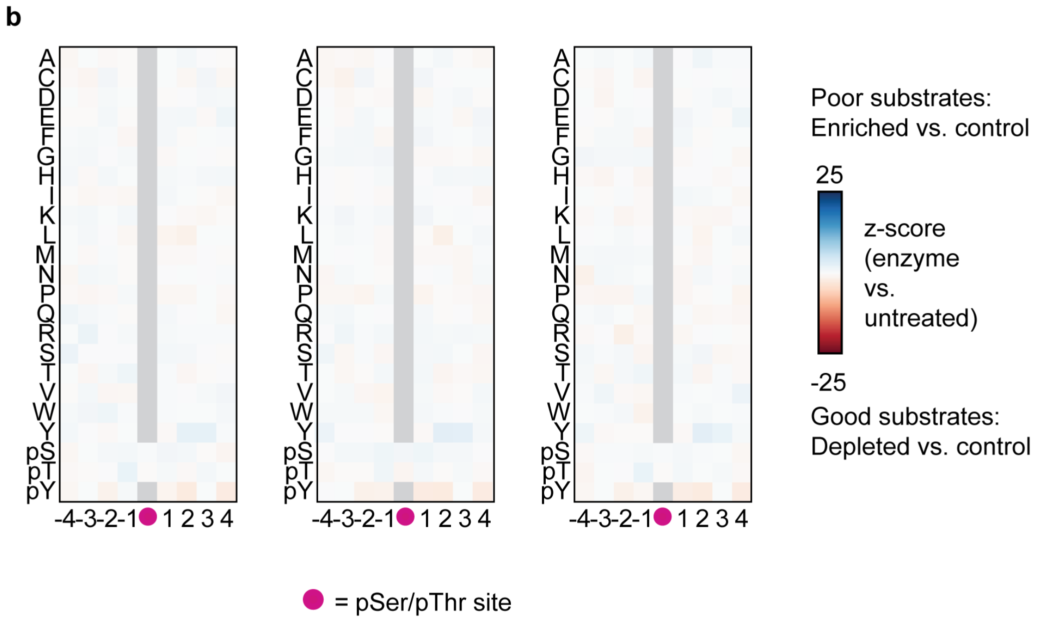

**Figure S22. PTP1B$_{1\text{-}321}$ specificity profiling with non-tryptic PhosPropels.** a) Heatmap for specificity profiling of PTP1B$_{1\text{-}321}$ at 180 min using GluC library generated from pervanadate-treated HEK293T cells. b) Heatmap for specificity profiling of PTP1B$_{1\text{-}321}$ at 180 min using LysC library generated from pervanadate-treated HEK293T cells. Z-scores were calculated by comparing positional frequencies to the 0 min timepoint using counts summed across n = 3 biological replicates. Residue-position combinations with Benjamini-Hochberg FDR-adjusted p-values < 0.0001 were considered significant and are outlined in black.

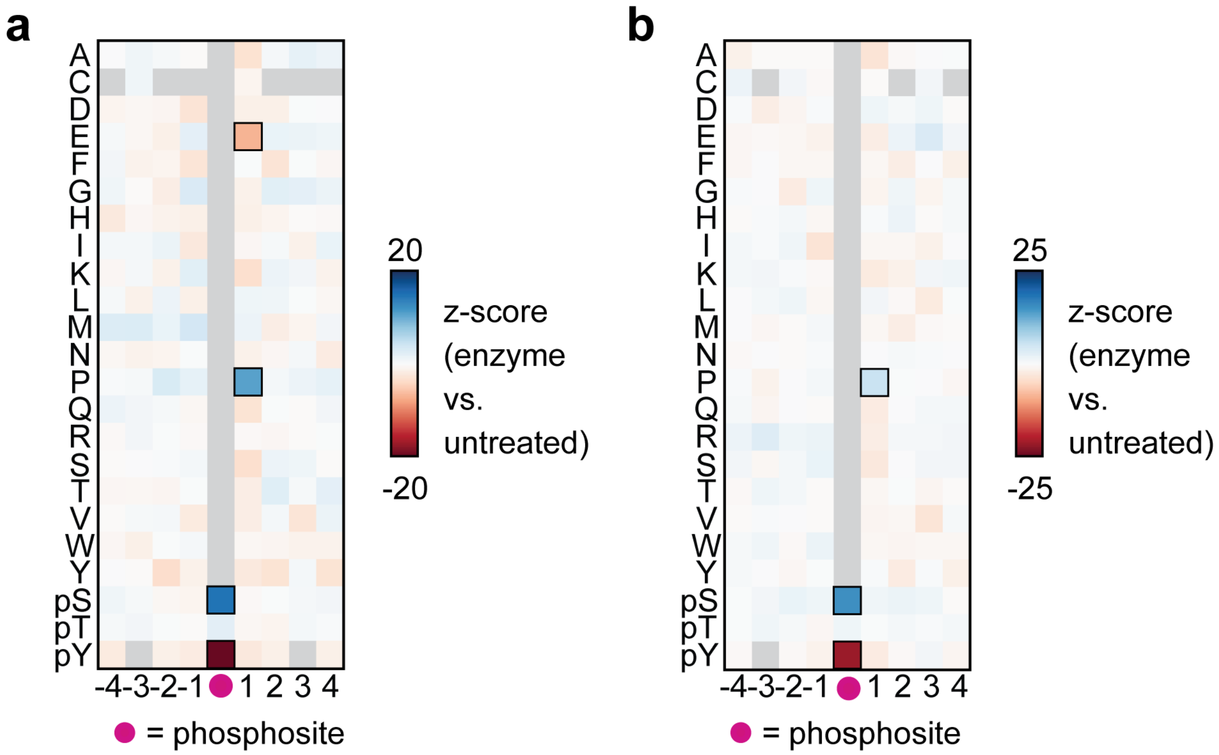

**Figure S23. Comparison of PTP1B specificity profiling experiments performed with PhosPropels generated with different proteases.** All experiments used PhosPropels generated from pervanadate-treated HEK293T cells. Dotted lines indicate z = ±4 (corresponding to α ≈ 0.0001). Points are colored according to residue identity (see legend). Points with |z| ≥ 4 of the same sign (upper right and lower left regions of the plot) represent residue-position combinations that were enriched or depleted consistently in both libraries. Points near the origin represent residue-position combinations with little or no enrichment or depletion in either dataset, whereas points exceeding the threshold in only one dataset indicate features enriched or depleted in one library but not the other. Residue-position combinations with large, opposing z-scores (|z| ≥ 4 in both datasets but with opposite sign) appear in the upper left and lower right regions of the plot and indicate protease-dependent differences in inferred specificity. These features are circled in red and labeled. a) Scatterplot comparing z scores obtained using trypsin PhosPropels and LysC PhosPropels. b) Scatterplot comparing z scores obtained using trypsin PhosPropels and GluC PhosPropels. c) Scatterplot comparing z scores obtained using GluC PhosPropels and LysC PhosPropels.

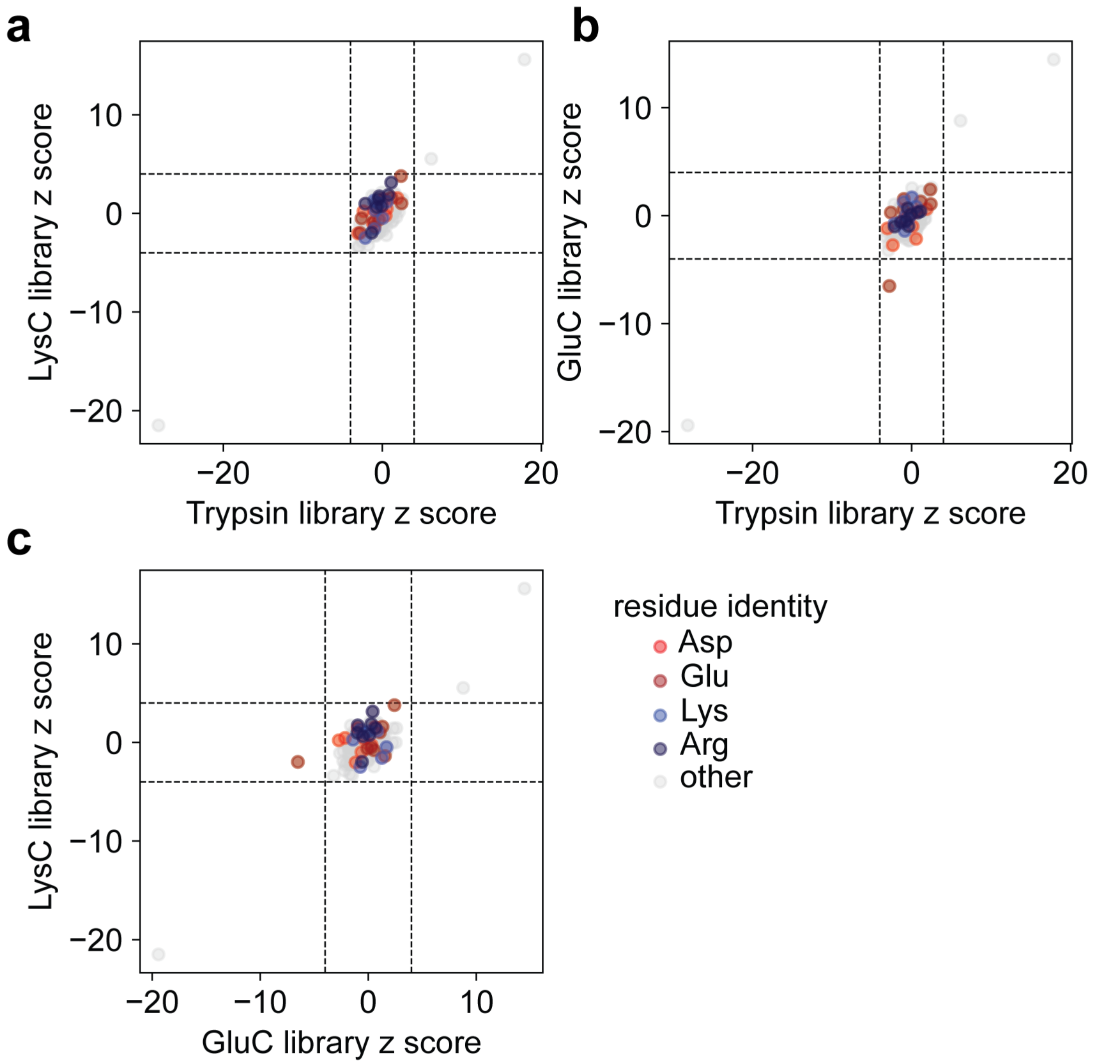

**Figure S24. Comparison of PTP1B pTyr specificity analyses performed with PhosPropels generated with different proteases.** All experiments used PhosPropels generated from pervanadate-treated HEK293T cells. Dotted lines indicate z = ±4 (corresponding to α ≈ 0.0001). Points are colored according to residue identity (see legend). Points with |z| ≥ 4 of the same sign (upper right and lower left regions of the plot) represent residue-position combinations that were enriched or depleted consistently in both libraries. Points near the origin represent residue-position combinations with little or no enrichment or depletion in either dataset, whereas points exceeding the threshold in only one dataset indicate features enriched or depleted in one library but not the other. Residue-position combinations with large, opposing z-scores (|z| ≥ 4 in both datasets but with opposite sign) appear in the upper left and lower right regions of the plot and indicate protease-dependent differences in inferred specificity. These features are circled in red and labeled. a) Scatterplot comparing z scores obtained using trypsin PhosPropels and LysC PhosPropels. b) Scatterplot comparing z scores obtained using trypsin PhosPropels and GluC PhosPropels. c) Scatterplot comparing z scores obtained using GluC PhosPropels and LysC PhosPropels.

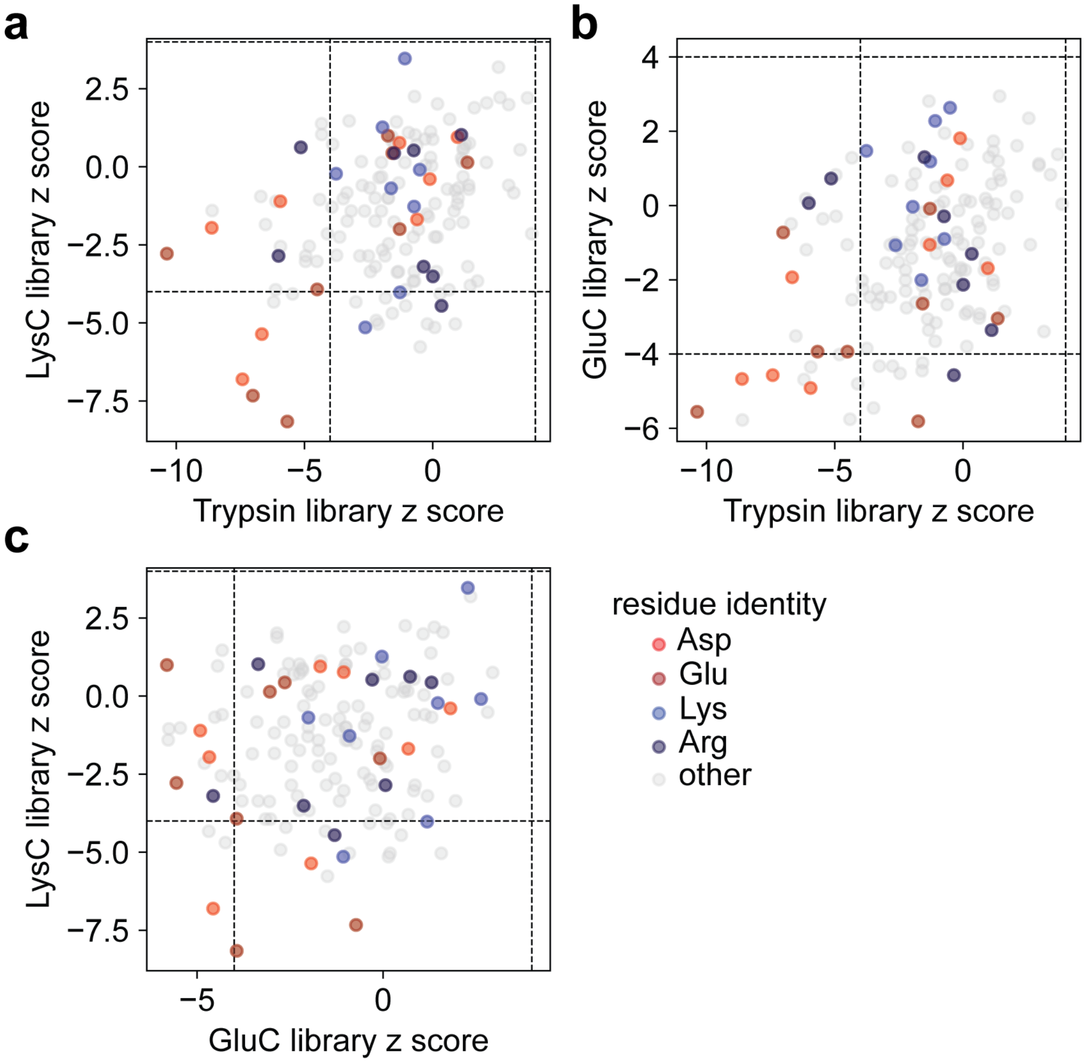

**Figure S25. PP2Ac-catalyzed phosphosite depletion in the presence of okadaic acid.** a) Treatment of PP2Ac with increasing concentrations of okadaic acid led to dose-dependent inhibition of pSer, pThr, and pTyr site depletion. Okadaic acid had no impact phosphosite depletion catalyzed by b) PTP1B, c) WipA, or d) WipB.

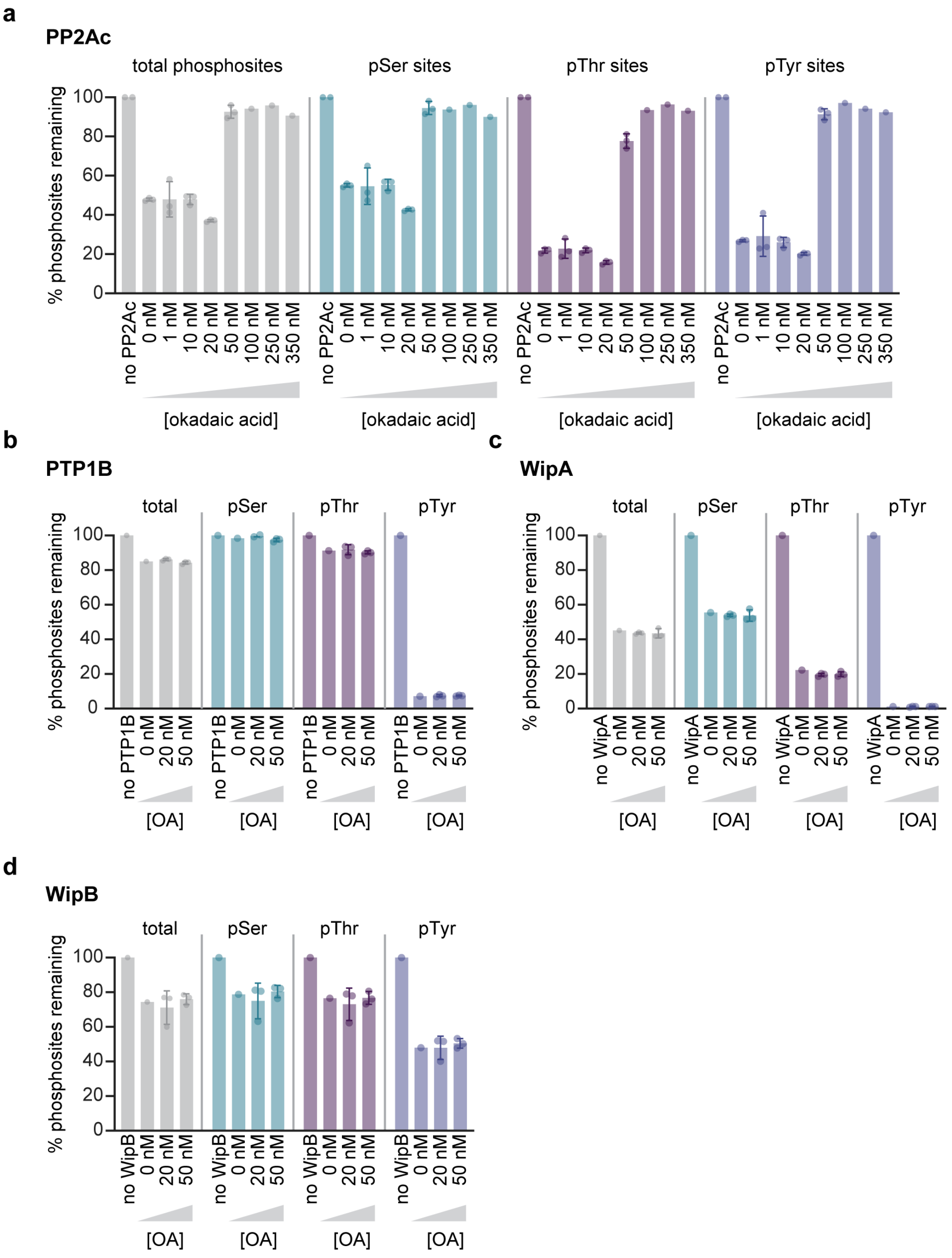

**Figure S26. PP2Ac heatmap timecourses.** a) Timecourse in which data were filtered to analyze only pSer sites. b) Timecourse in which data were filtered to analyze only pThr sites. c) Timecourse in which data were filtered to analyze only pTyr sites. Z-scores were calculated by comparing positional frequencies to the 0 min timepoint using counts summed across n = 3 biological replicates after filtering for the indicated phosphosite type. Residue-position combinations with Benjamini-Hochberg FDR-adjusted p-values < 0.0001 were considered significant and are outlined in black.

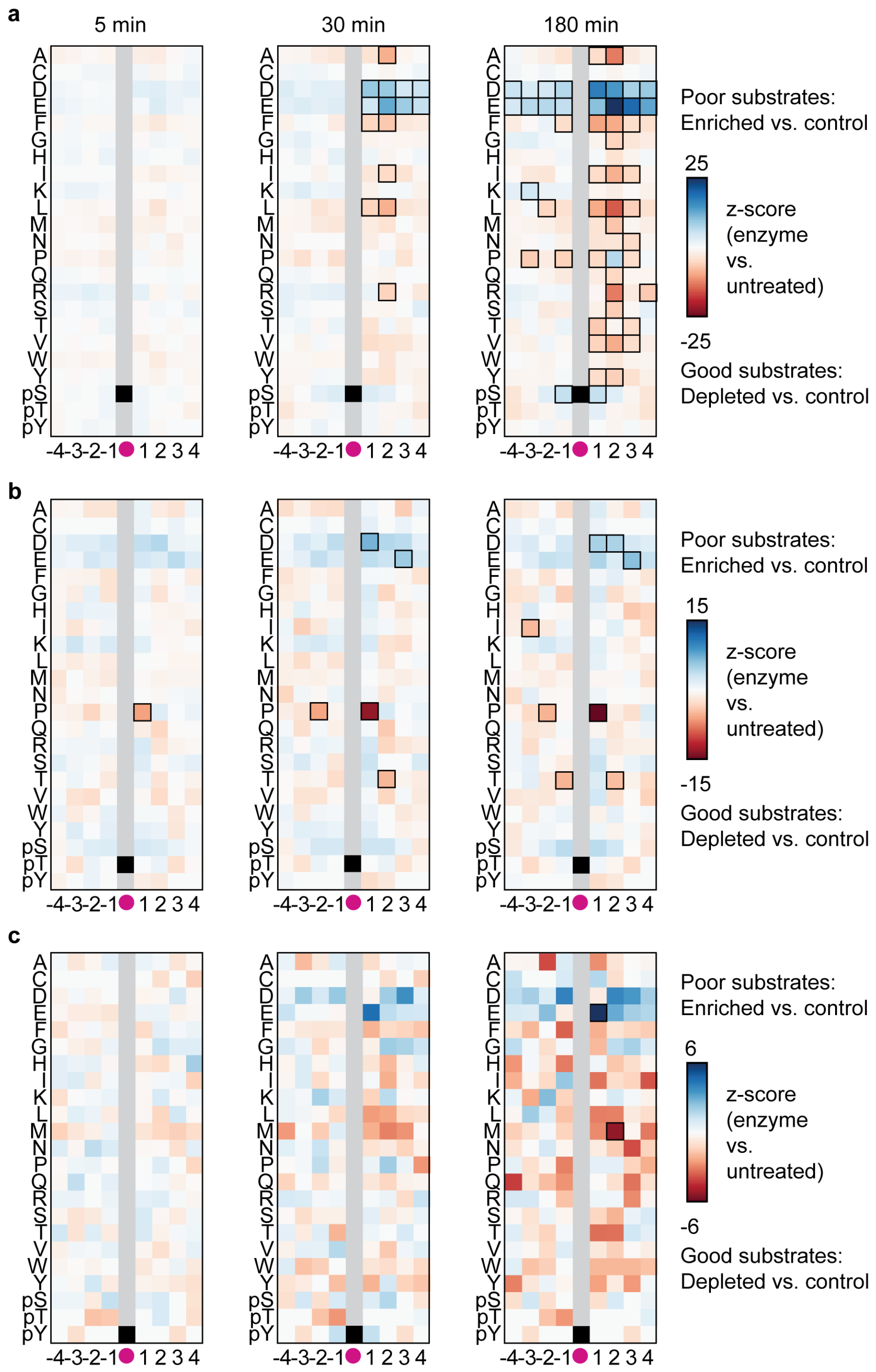

**Figure S27. PP2Ac specificity profiling with non-tryptic PhosPropels.** a) Heatmap for specificity profiling of PP2Ac at 180 min using GluC library generated from pervanadate-treated HEK293T cells. b) Heatmap for specificity profiling of PP2Ac at 180 min using LysC library generated from pervanadate-treated HEK293T cells. Z-scores were calculated by comparing positional frequencies to the 0 min timepoint using counts summed across n = 3 biological replicates. Residue-position combinations with Benjamini-Hochberg FDR-adjusted p-values < 0.0001 were considered significant and are outlined in black.

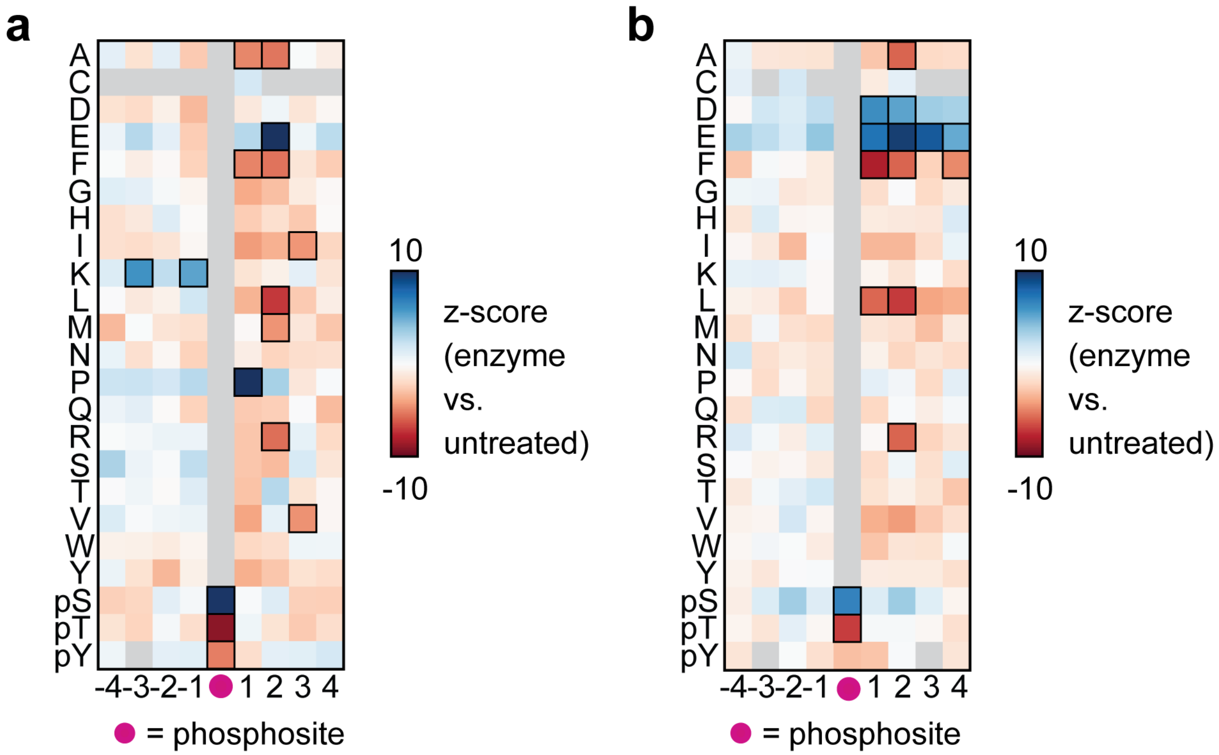

**Figure S28. Comparison of PP2Ac specificity profiling experiments performed with PhosPropels generated with different proteases.** All experiments used PhosPropels generated from pervanadate-treated HEK293T cells. Dotted lines indicate z = ±4 (corresponding to α ≈ 0.0001). Points are colored according to residue identity (see legend). Points with |z| ≥ 4 of the same sign (upper right and lower left regions of the plot) represent residue-position combinations that were enriched or depleted consistently in both libraries. Points near the origin represent residue-position combinations with little or no enrichment or depletion in either dataset, whereas points exceeding the threshold in only one dataset indicate features enriched or depleted in one library but not the other. Residue-position combinations with large, opposing z-scores (|z| ≥ 4 in both datasets but with opposite sign) appear in the upper left and lower right regions of the plot and indicate protease-dependent differences in inferred specificity. These features are circled in red and labeled. a) Scatterplot comparing z scores obtained using trypsin PhosPropels and LysC PhosPropels. b) Scatterplot comparing z scores obtained using trypsin PhosPropels and GluC PhosPropels. c) Scatterplot comparing z scores obtained using GluC PhosPropels and LysC PhosPropels.

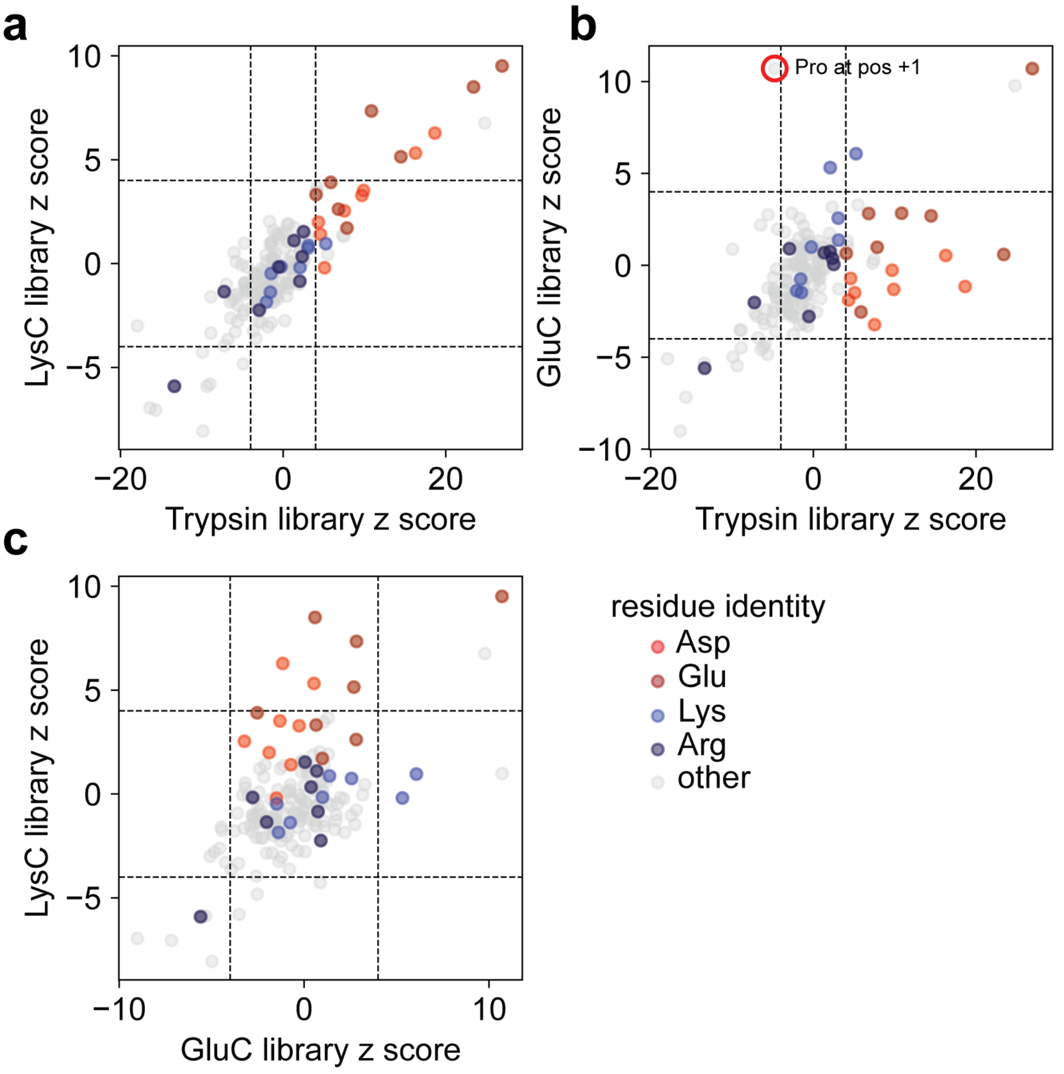

**Figure S29. Comparison of PP2Ac pSer/pThr analyses performed with PhosPropels generated with different proteases.** All experiments used PhosPropels generated from pervanadate-treated HEK293T cells. Dotted lines indicate z = ±4 (corresponding to α ≈ 0.0001). Points are colored according to residue identity (see legend). Points with |z| ≥ 4 of the same sign (upper right and lower left regions of the plot) represent residue-position combinations that were enriched or depleted consistently in both libraries. Points near the origin represent residue-position combinations with little or no enrichment or depletion in either dataset, whereas points exceeding the threshold in only one dataset indicate features enriched or depleted in one library but not the other. Residue-position combinations with large, opposing z-scores (|z| ≥ 4 in both datasets but with opposite sign) appear in the upper left and lower right regions of the plot and indicate protease-dependent differences in inferred specificity. These features are circled in red and labeled. a) Scatterplot comparing z scores obtained using trypsin PhosPropels and LysC PhosPropels. b) Scatterplot comparing z scores obtained using trypsin PhosPropels and GluC PhosPropels. c) Scatterplot comparing z scores obtained using GluC PhosPropels and LysC PhosPropels.

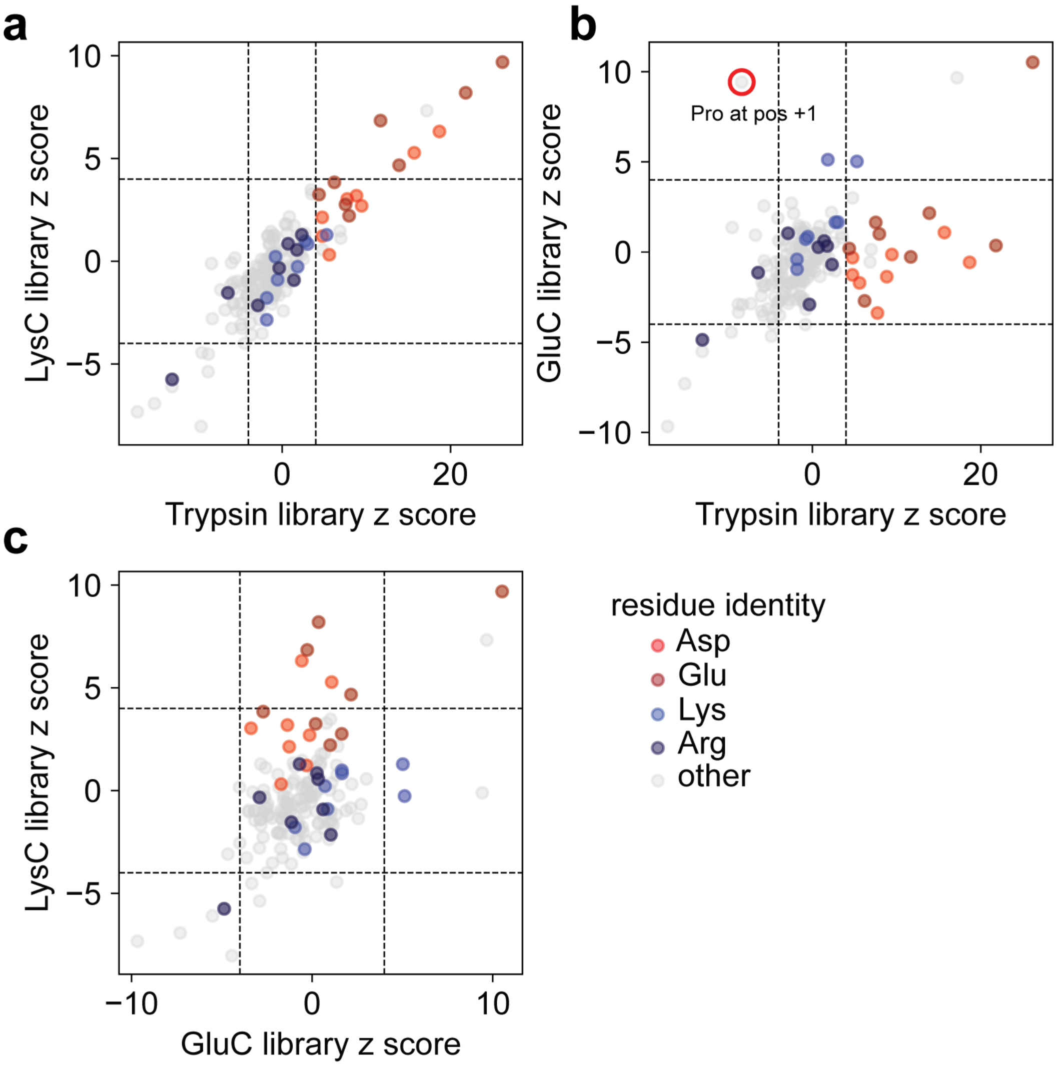

**Figure S30. WipA heatmap timecourses.** a) Timecourse in which all pSer, pThr, and pTyr sites were analyzed. b) Timecourse in which data were filtered to analyze only pSer and pThr sites. Z-scores were calculated by comparing positional frequencies to the 0 min timepoint using counts summed across n = 3 biological replicates after filtering for the indicated phosphosite type. Residue-position combinations with Benjamini-Hochberg FDR-adjusted p-values < 0.0001 were considered significant and are outlined in black.

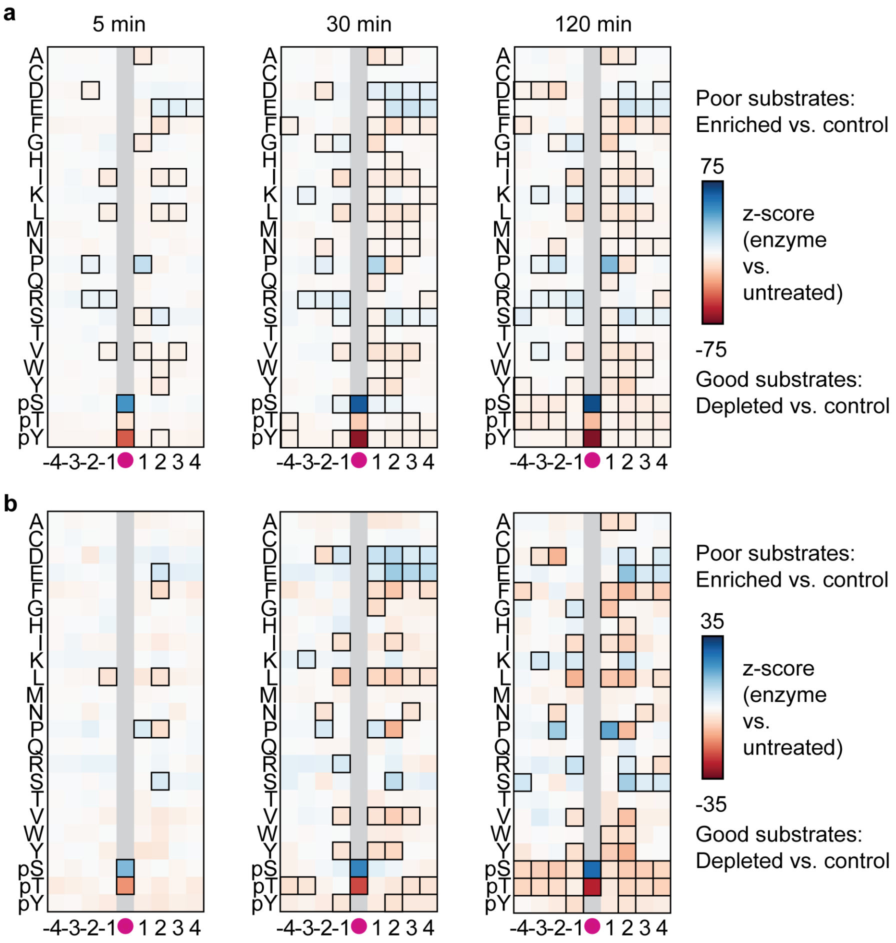

**Figure S31. WipB heatmap timecourses.** a) Timecourse in which all pSer, pThr, and pTyr sites were analyzed. b) Timecourse in which data were filtered to analyze only pSer and pThr sites. c) Timecourse in which data were filtered to analyze only pTyr sites. Z-scores were calculated by comparing positional frequencies to the 0 min timepoint using counts summed across n = 3 biological replicates after filtering for the indicated phosphosite type. Residue-position combinations with Benjamini-Hochberg FDR-adjusted p-values < 0.0001 were considered significant and are outlined in black.

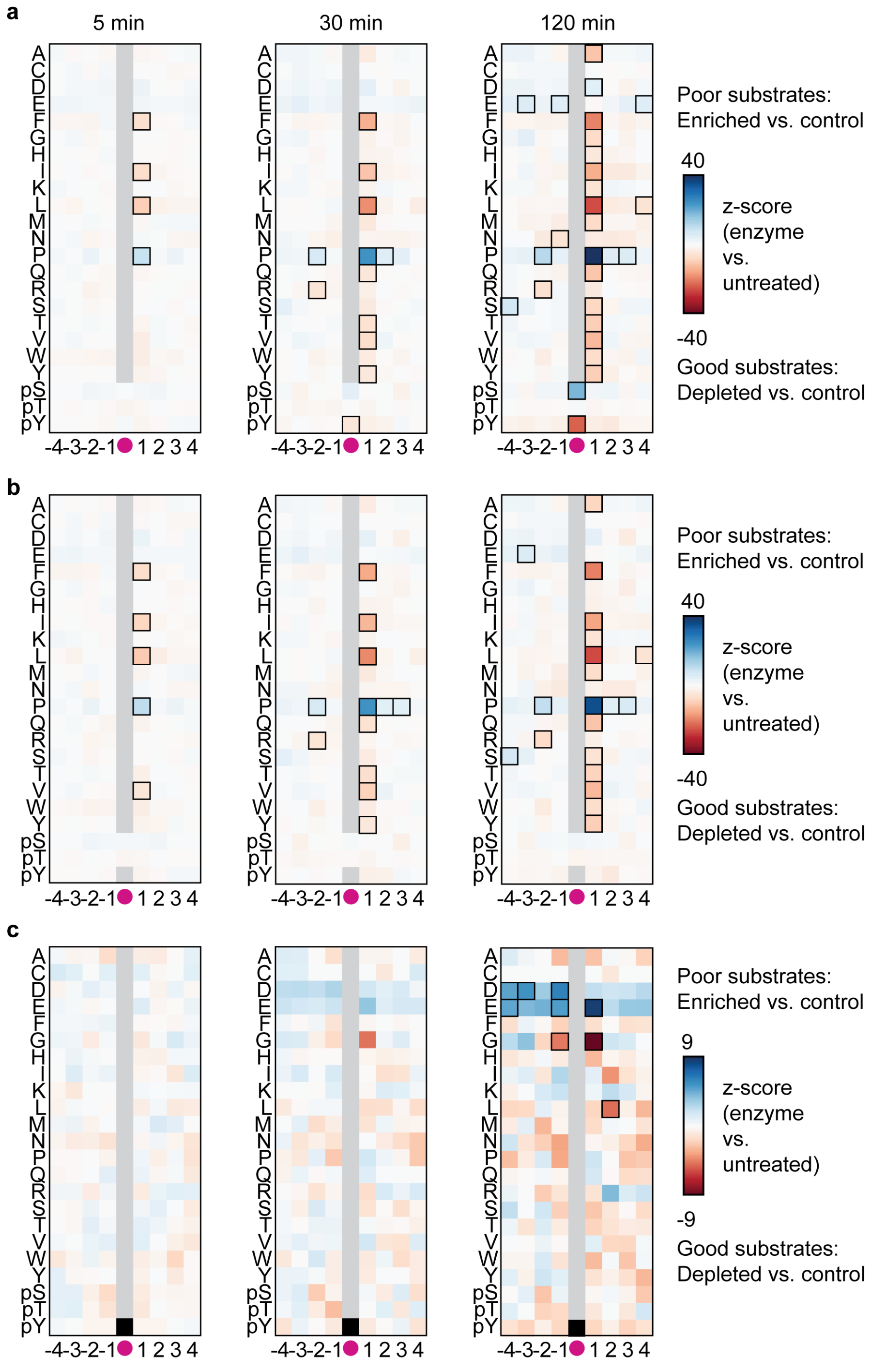

**Figure S32. WipA specificity profiling with non-tryptic PhosPropels.** a) Heatmap for specificity profiling of WipA at 120 min using GluC library generated from pervanadate-treated HEK293T cells. b) Heatmap for specificity profiling of WipA at 120 min using LysC library generated from pervanadate-treated HEK293T cells. Z-scores were calculated by comparing positional frequencies to the 0 min timepoint using counts summed across n = 3 biological replicates. Residue-position combinations with Benjamini-Hochberg FDR-adjusted p-values < 0.0001 were considered significant and are outlined in black.

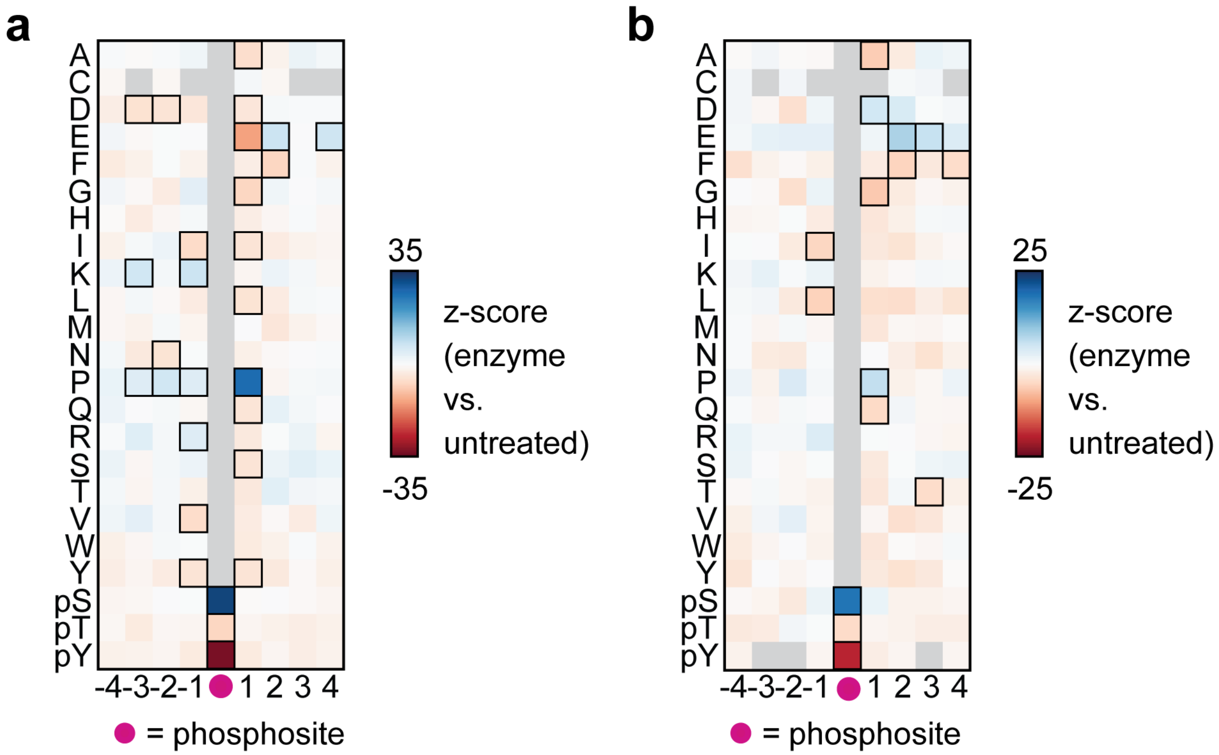

**Figure S33. WipB specificity profiling with non-tryptic PhosPropels.** a) Heatmap for specificity profiling of WipB at 120 min using GluC library generated from pervanadate-treated HEK293T cells. b) Heatmap for specificity profiling of WipB at 120 min using LysC library generated from pervanadate-treated HEK293T cells. Z-scores were calculated by comparing positional frequencies to the 0 min timepoint using counts summed across n = 3 biological replicates. Residue-position combinations with Benjamini-Hochberg FDR-adjusted p-values < 0.0001 were considered significant and are outlined in black.

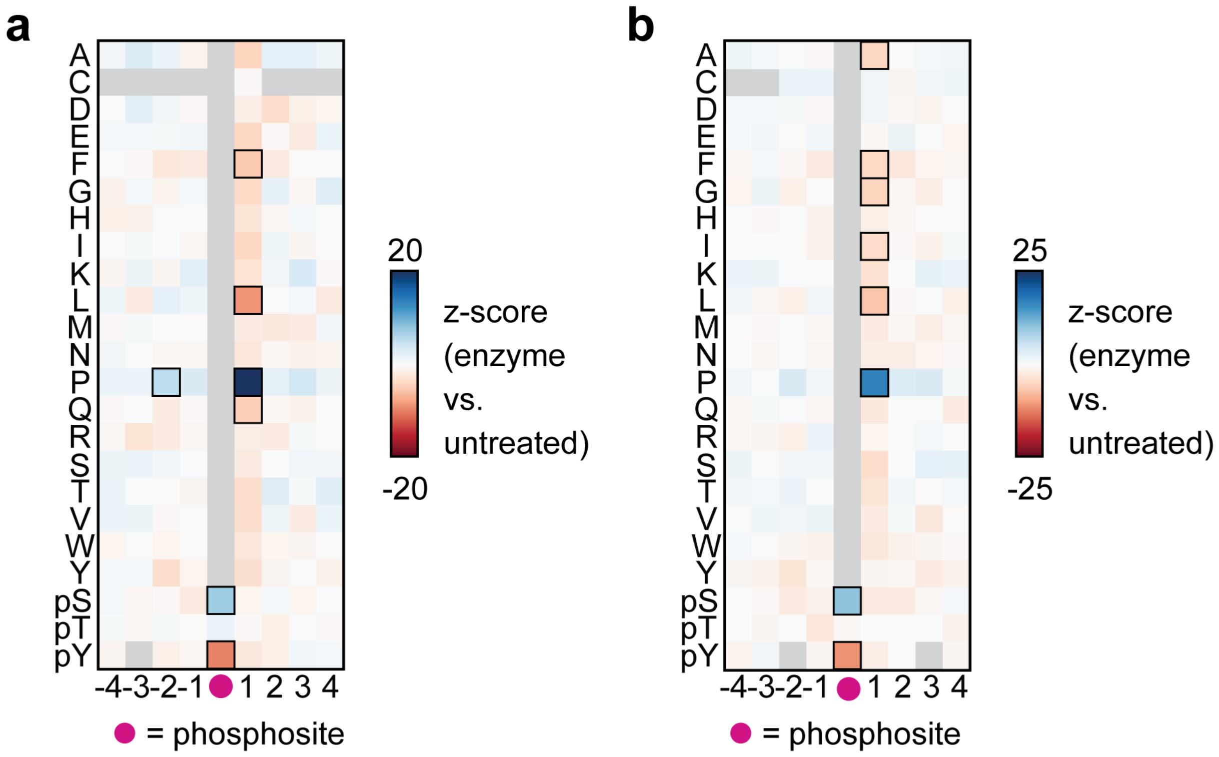

**Figure S34. Comparison of WipA specificity profiling experiments performed with PhosPropels generated with different proteases.** All experiments used PhosPropels generated from pervanadate-treated HEK293T cells. Dotted lines indicate z = ±4 (corresponding to α ≈ 0.0001). Points are colored according to residue identity (see legend). Points with |z| ≥ 4 of the same sign (upper right and lower left regions of the plot) represent residue-position combinations that were enriched or depleted consistently in both libraries. Points near the origin represent residue-position combinations with little or no enrichment or depletion in either dataset, whereas points exceeding the threshold in only one dataset indicate features enriched or depleted in one library but not the other. Residue-position combinations with large, opposing z-scores (|z| ≥ 4 in both datasets but with opposite sign) appear in the upper left and lower right regions of the plot and indicate protease-dependent differences in inferred specificity. These features are circled in red and labeled. a) Scatterplot comparing z scores obtained using trypsin PhosPropels and LysC PhosPropels. b) Scatterplot comparing z scores obtained using trypsin PhosPropels and GluC PhosPropels. c) Scatterplot comparing z scores obtained using GluC PhosPropels and LysC PhosPropels.

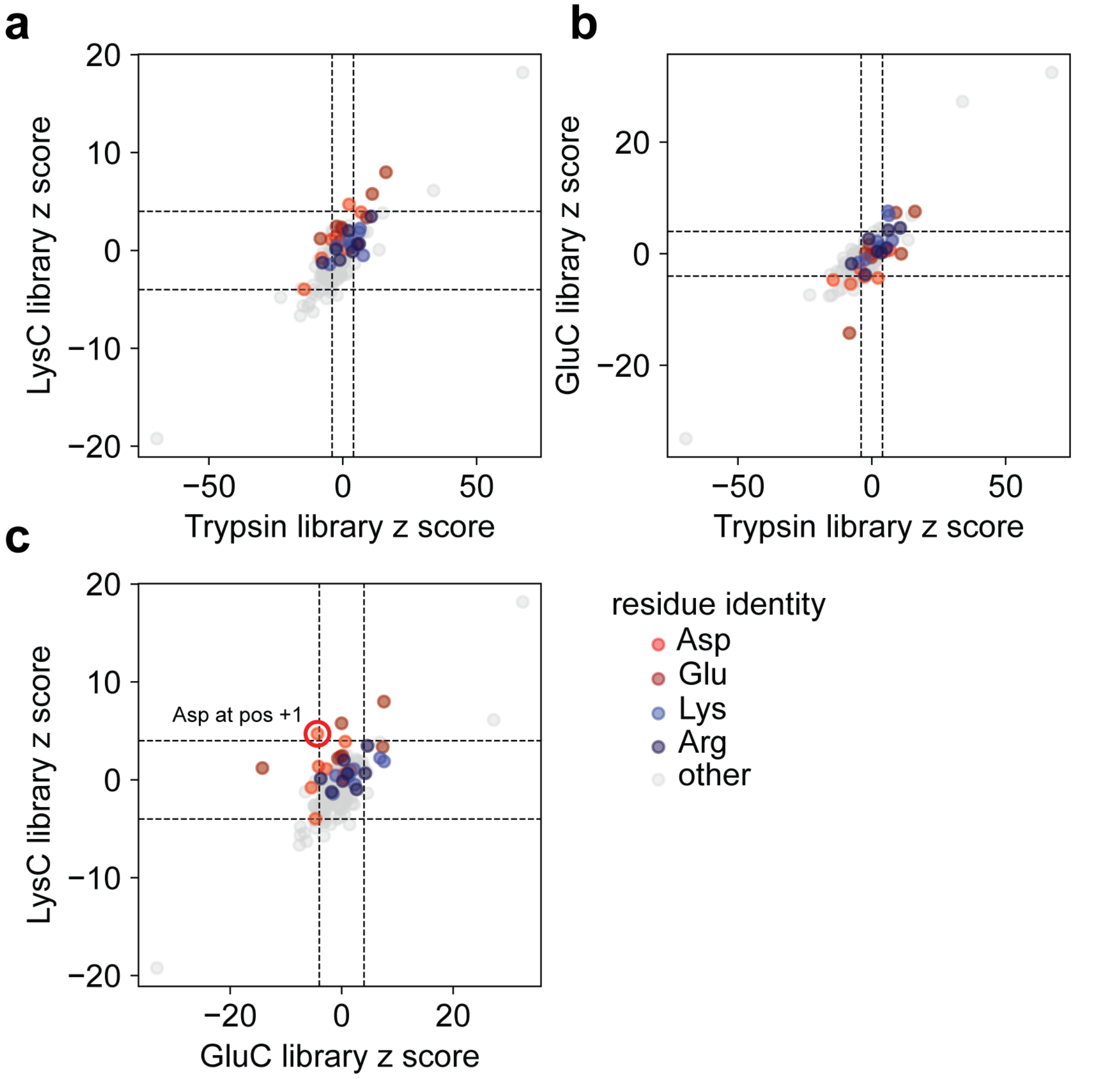

**Figure S35. Comparison of WipA pTyr specificity analyses performed with PhosPropels generated with different proteases.** All experiments used PhosPropels generated from pervanadate-treated HEK293T cells. Dotted lines indicate z = ±4 (corresponding to α ≈ 0.0001). Points are colored according to residue identity (see legend). Points with |z| ≥ 4 of the same sign (upper right and lower left regions of the plot) represent residue-position combinations that were enriched or depleted consistently in both libraries. Points near the origin represent residue-position combinations with little or no enrichment or depletion in either dataset, whereas points exceeding the threshold in only one dataset indicate features enriched or depleted in one library but not the other. Residue-position combinations with large, opposing z-scores (|z| ≥ 4 in both datasets but with opposite sign) appear in the upper left and lower right regions of the plot and indicate protease-dependent differences in inferred specificity. These features are circled in red and labeled. a) Scatterplot comparing z scores obtained using trypsin PhosPropels and LysC PhosPropels. b) Scatterplot comparing z scores obtained using trypsin PhosPropels and GluC PhosPropels. c) Scatterplot comparing z scores obtained using GluC PhosPropels and LysC PhosPropels.

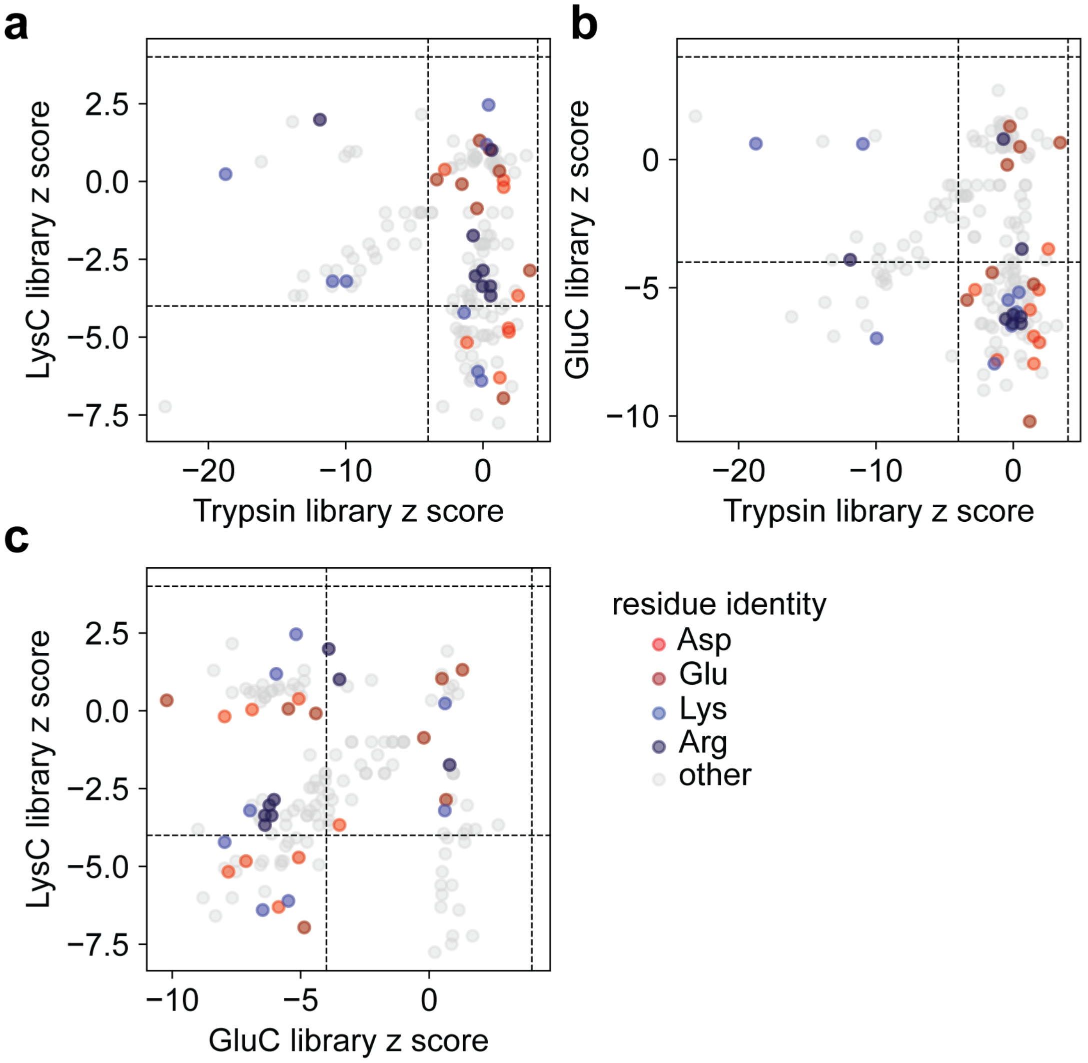

**Figure S36. Comparison of WipB specificity profiling experiments performed with PhosPropels generated with different proteases.** All experiments used PhosPropels generated from pervanadate-treated HEK293T cells. Dotted lines indicate z = ±4 (corresponding to α ≈ 0.0001). Points are colored according to residue identity (see legend). Points with |z| ≥ 4 of the same sign (upper right and lower left regions of the plot) represent residue-position combinations that were enriched or depleted consistently in both libraries. Points near the origin represent residue-position combinations with little or no enrichment or depletion in either dataset, whereas points exceeding the threshold in only one dataset indicate features enriched or depleted in one library but not the other. Residue-position combinations with large, opposing z-scores (|z| ≥ 4 in both datasets but with opposite sign) appear in the upper left and lower right regions of the plot and indicate protease-dependent differences in inferred specificity. These features are circled in red and labeled. a) Scatterplot comparing z scores obtained using trypsin PhosPropels and LysC PhosPropels. b) Scatterplot comparing z scores obtained using trypsin PhosPropels and GluC PhosPropels. c) Scatterplot comparing z scores obtained using GluC PhosPropels and LysC PhosPropels.

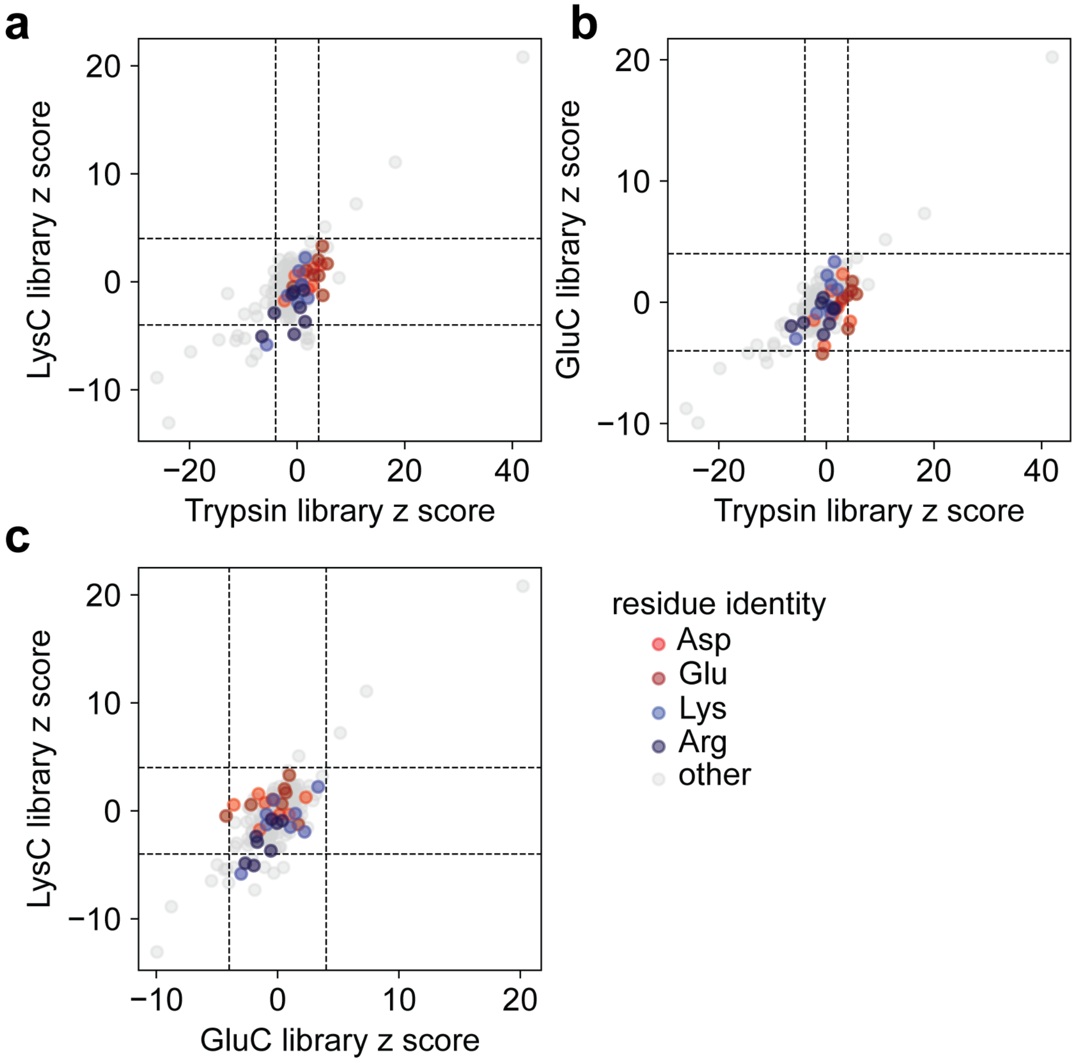

**Figure S37. Comparison of WipB specificity analyses for pSer and pThr with Pro at +1 excluded performed with PhosPropels generated with different proteases.** All experiments used PhosPropels generated from pervanadate-treated HEK293T cells. Dotted lines indicate z = ±4 (corresponding to α ≈ 0.0001). Points are colored according to residue identity (see legend). Points with |z| ≥ 4 of the same sign (upper right and lower left regions of the plot) represent residue-position combinations that were enriched or depleted consistently in both libraries. Points near the origin represent residue-position combinations with little or no enrichment or depletion in either dataset, whereas points exceeding the threshold in only one dataset indicate features enriched or depleted in one library but not the other. Residue-position combinations with large, opposing z-scores (|z| ≥ 4 in both datasets but with opposite sign) appear in the upper left and lower right regions of the plot and indicate protease-dependent differences in inferred specificity. These features are circled in red and labeled. a) Scatterplot comparing z scores obtained using trypsin PhosPropels and LysC PhosPropels. b) Scatterplot comparing z scores obtained using trypsin PhosPropels and GluC PhosPropels. c) Scatterplot comparing z scores obtained using GluC PhosPropels and LysC PhosPropels.

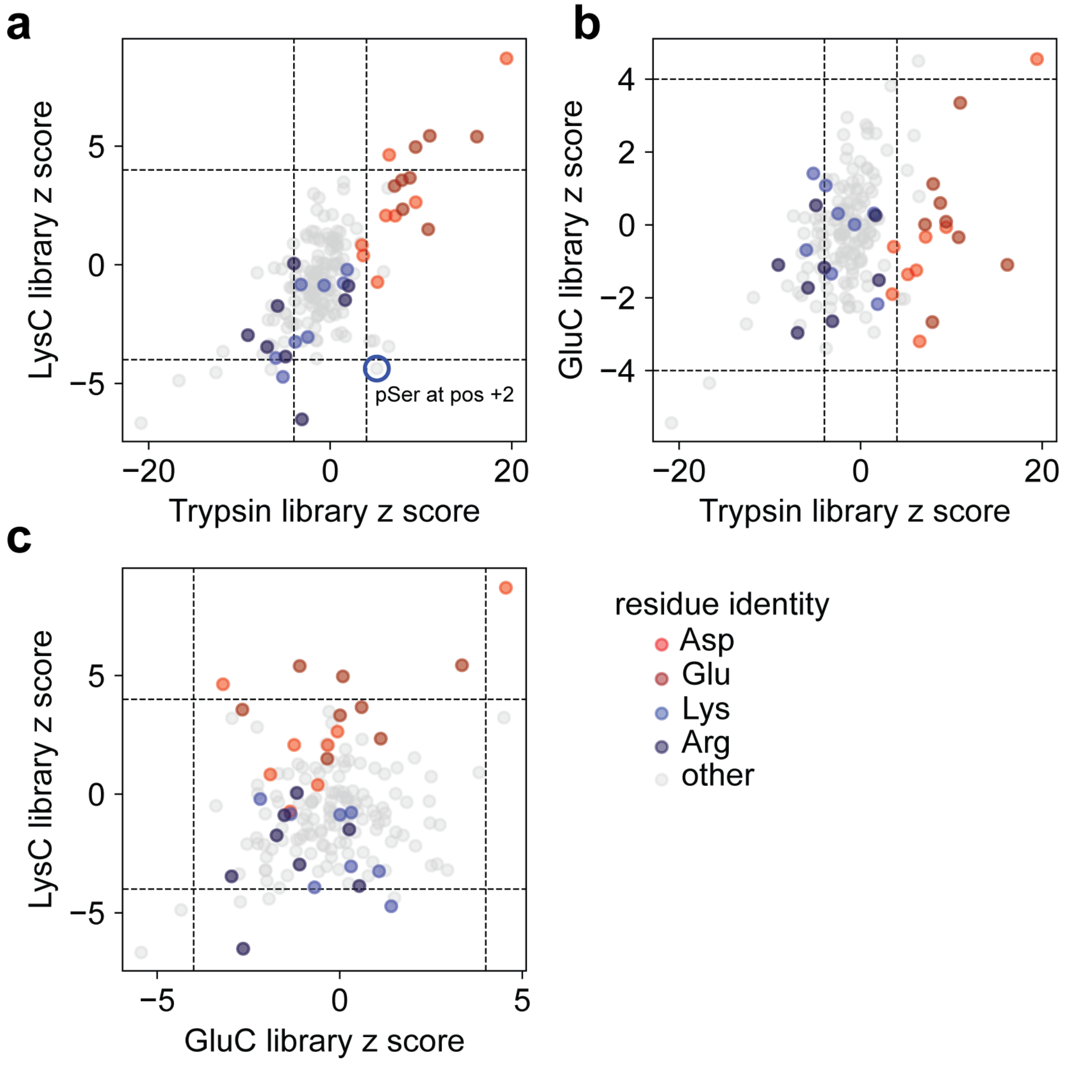

**Figure S38. Distribution of ptmRS site localization scores for β-eliminated sites in OspF-treated PhosPropels from pervanadate-treated HEK293T cells.** Scores were computed using the IMP-ptmRS node in Proteome Discoverer 2.4 (ThermoFisher Scientific). Only sites with scores >90 were included in the analysis.

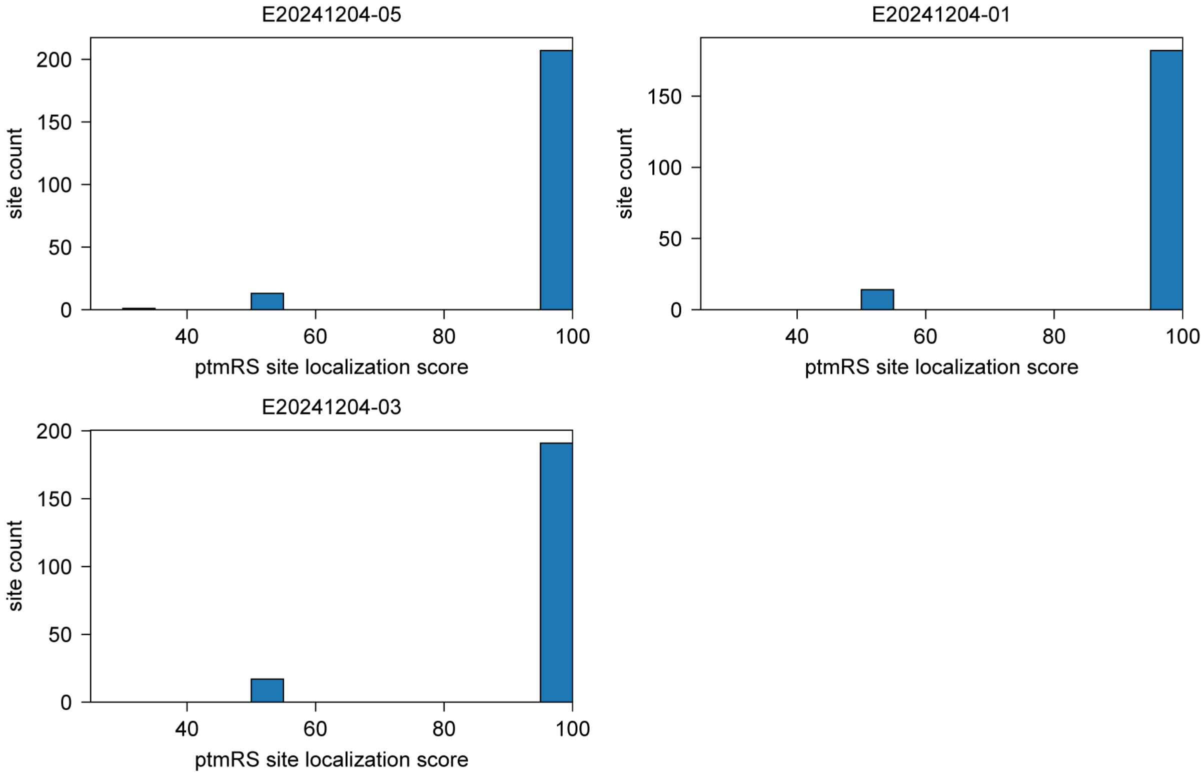

**Figure S39. OspF specificity profiling with non-tryptic PhosPropels.** a) Heatmap for specificity profiling of OspF at 24 h using chymotrypsin library generated from pervanadate-treated HEK293T cells. b) Heatmap for specificity profiling of OspF at 24 h min using LysC library generated from pervanadate-treated HEK293T cells. Z-scores were calculated by comparing positional frequencies to the 0 min timepoint using counts summed across n = 3 biological replicates. Residue-position combinations with Benjamini-Hochberg FDR-adjusted p-values < 0.0001 were considered significant and are outlined in black.

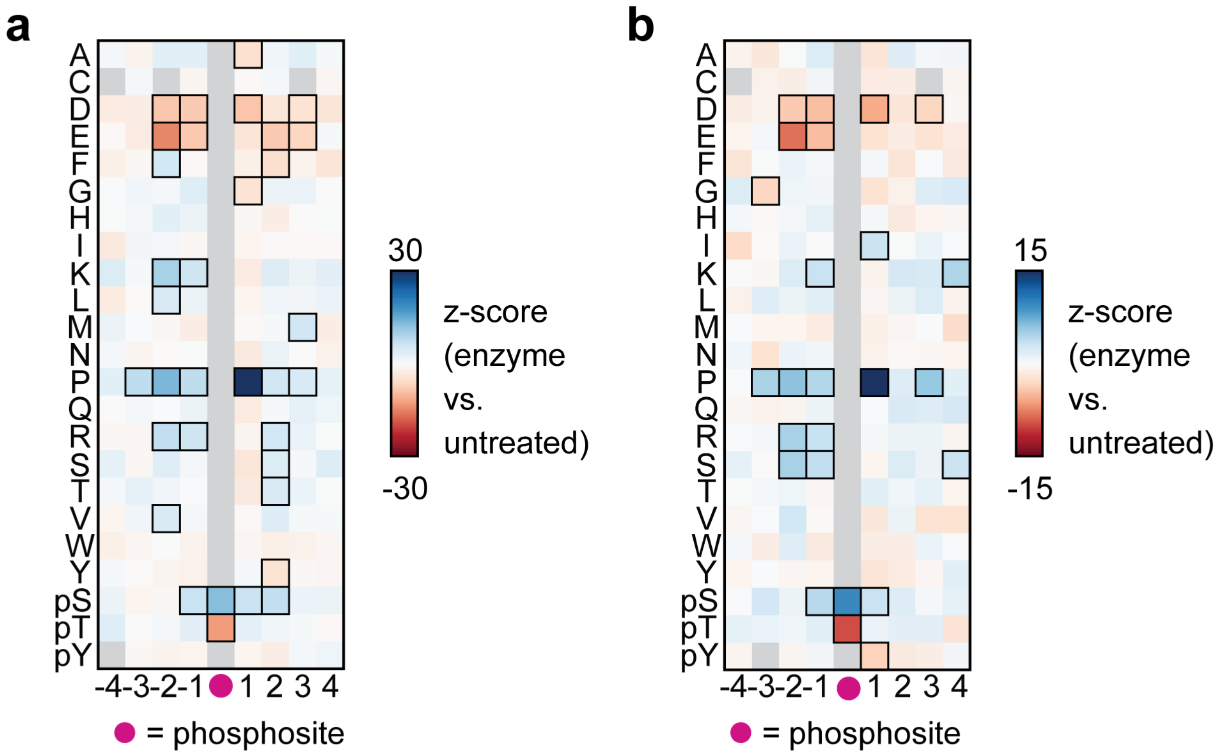

**Figure S40. Comparison of OspF specificity profiling experiments performed with PhosPropels generated with different proteases.** All experiments used PhosPropels generated from pervanadate-treated HEK293T cells. Dotted lines indicate z = ±4 (corresponding to α ≈ 0.0001). Points are colored according to residue identity (see legend). Points with |z| ≥ 4 of the same sign (upper right and lower left regions of the plot) represent residue-position combinations that were enriched or depleted consistently in both libraries. Points near the origin represent residue-position combinations with little or no enrichment or depletion in either dataset, whereas points exceeding the threshold in only one dataset indicate features enriched or depleted in one library but not the other. Residue-position combinations with large, opposing z-scores (|z| ≥ 4 in both datasets but with opposite sign) appear in the upper left and lower right regions of the plot and indicate protease-dependent differences in inferred specificity. These features are circled in red and labeled. a) Scatterplot comparing z scores obtained using trypsin PhosPropels and LysC PhosPropels. b) Scatterplot comparing z scores obtained using trypsin PhosPropels and GluC PhosPropels. c) Scatterplot comparing z scores obtained using GluC PhosPropels and LysC PhosPropels.

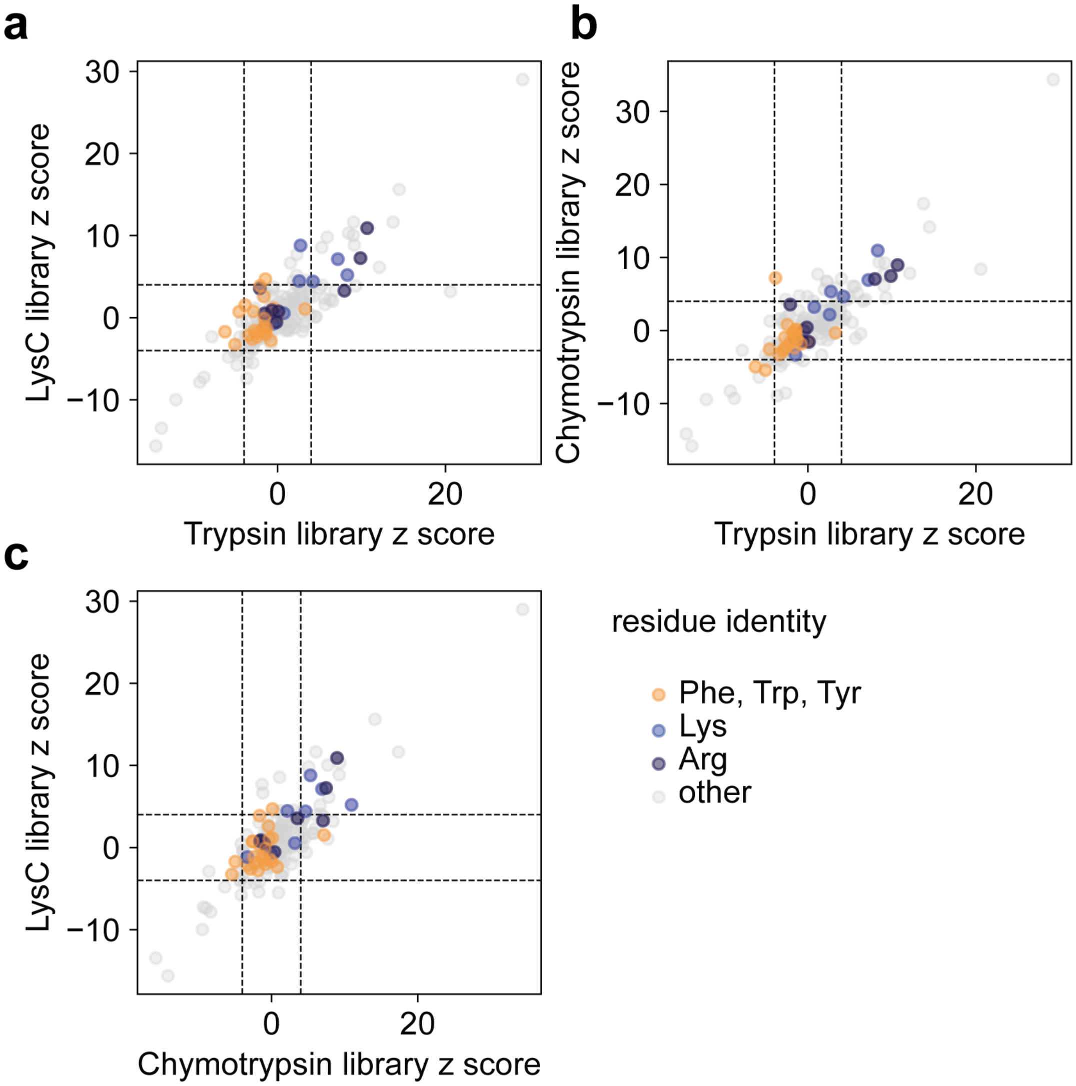

**Figure S41. Characterization of the pThr lyases SpvC and HopAI using PhosPropels.** a) SpvC catalyzes β-elimination on PhosPropels with a similar specificity profile to OspF. The fractions of sites β-eliminated in treated (purple) vs. untreated (grey) samples were compared using unpaired *t*-tests and p-values were corrected for multiple comparisons using the Holm-Šídák method. **** $p < 0.0001$. b) The specificity profile of SpvC reveals that pThr sites, sites with acidic flanking residues, and Phe, Tyr, or pTyr at +2 are favored, while +1 Pro is disfavored. c) HopAI catalyzes β-elimination on PhosPropels with lower activity than OspF or SpvC. The fractions of sites β-eliminated in treated (purple) vs. untreated (grey) samples were compared using unpaired *t*-tests and p-values were corrected for multiple comparisons using the Holm-Šídák method. * $p < 0.05$.

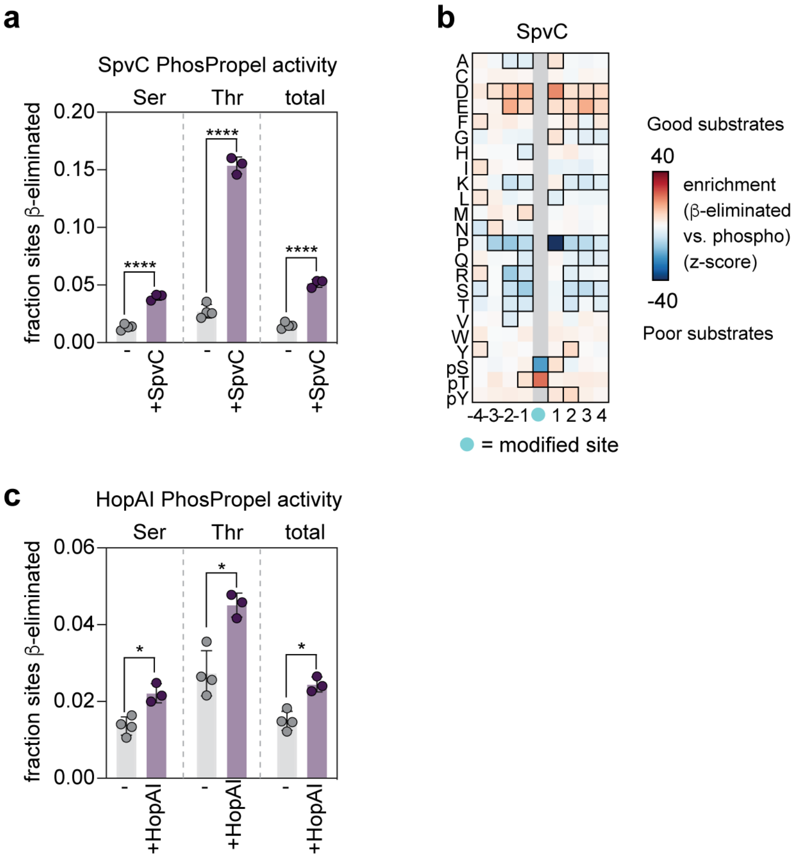

**Figure S42. Multiple sequence alignment of OspF, SpvC, and HopAI.** Multiple sequence alignment was performed with MUSCLE. Catalytic residues are highlighted in red, phosphate-coordinating residues are highlighting in blue, and other positions targeted for mutagenesis in OspF are highlighted in orange. OspF and SpvC share 63% sequence identify, while HopAI is more diverged.

```
HopAI           ------MLALKLNT-------SIAQAPLKKNAEAELRHMNHAEVRAHTPTRFTLNHRAPT 47
OspF            MPIKKPCLKLNLDSLNVVKS-EIPQMLSANERLKNNFNILYNQIRQYPAYYFKVASNVPT 59
SpvC            MPINRPNLNLNIPPLNIVAAYDGAEIPSTNKHLKNNFNSLHNQMRKMPVSHFKEALDVPD 60
                       * *:: .       . .:    ::   :  :  : ::*  .   *.    .*

HopAI           Y-EVAQSALGENHGGWTAVNKFKVTESEVFIHMERSDSRSKGDFAGDKIHLSVAPQHVAS 106
OspF            YSDICQF-FSVMYQGFQIVNH----SGDVFIHACRENPQSKGDFVGDKFHISIAREQVPL 114
SpvC            YSGMRQSGFFAMSQGFQLNNH----GYDVFIHARRESPQSQGKFAGDKFHISVLRDMVPQ 116
                *  : *  :     *:   *:      :****  *....*:*.*.***:*:*:  : *.

HopAI           AFNAIGKILQADDSPVDKWKVTDMSCASSDLQPEKKRVTQGAQFTLYAKPDRADNTYSPE 166
OspF            AFQILSGLLFSEDSPIDKWKITDMNRVS-----QQSRVGIGAQFTLYVKSDQECSQYSAL 169
SpvC            AFQALSGLLFSEDSPVDKWKVTDMEKVV-----QQARVSLGAQFTLYIKPDQENSQYSAS 171
                **: :. :* ::***:****:***. .      :: **  ******* *.*.  . **.

HopAI           YMGKMRGMISSIERELHTAGVQQSNNRPASDVAPGHWAYASYRNEHRSERAGSSSQANEL 226
OspF            LLHKIRQFIMCLESNLLRSKI-APGEYPASDVRPEDWKYVSYRNELRSDRDGSERQEQML 228
SpvC            FLHKTRQFIECLESRLSENGV-ISGQCPESDVHPENWKYLSYRNELRSGRDGGEMQRQAL 230
                 : * * :* .:*  *    :  ..: * *** *  * * ***** ** * *.. * : *

HopAI           EKEPFFQLVSFPDVAASPVKSGASSRSLMPPPWTR 261
OspF            REEPFYRLMIE------------------------ 239
SpvC            REEPFYRLMTE------------------------ 241
                 :***:.*:
```

* Fully conserved
: Conserved, highly similar
. Conserved, weakly similar

**Figure S43. OspF$_{27\text{-}239}$ specificity profiling with non-tryptic PhosPropels.** a) Heatmap for specificity profiling of OspF$_{27\text{-}239}$ at 24 h using chymotrypsin library generated from pervanadate-treated HEK293T cells. b) Heatmap for specificity profiling of OspF$_{27\text{-}239}$ at 24 h min using LysC library generated from pervanadate-treated HEK293T cells. Z-scores were calculated by comparing positional frequencies to the 0 min timepoint using counts summed across n = 3 biological replicates. Residue-position combinations with Benjamini-Hochberg FDR-adjusted p-values < 0.0001 were considered significant and are outlined in black.

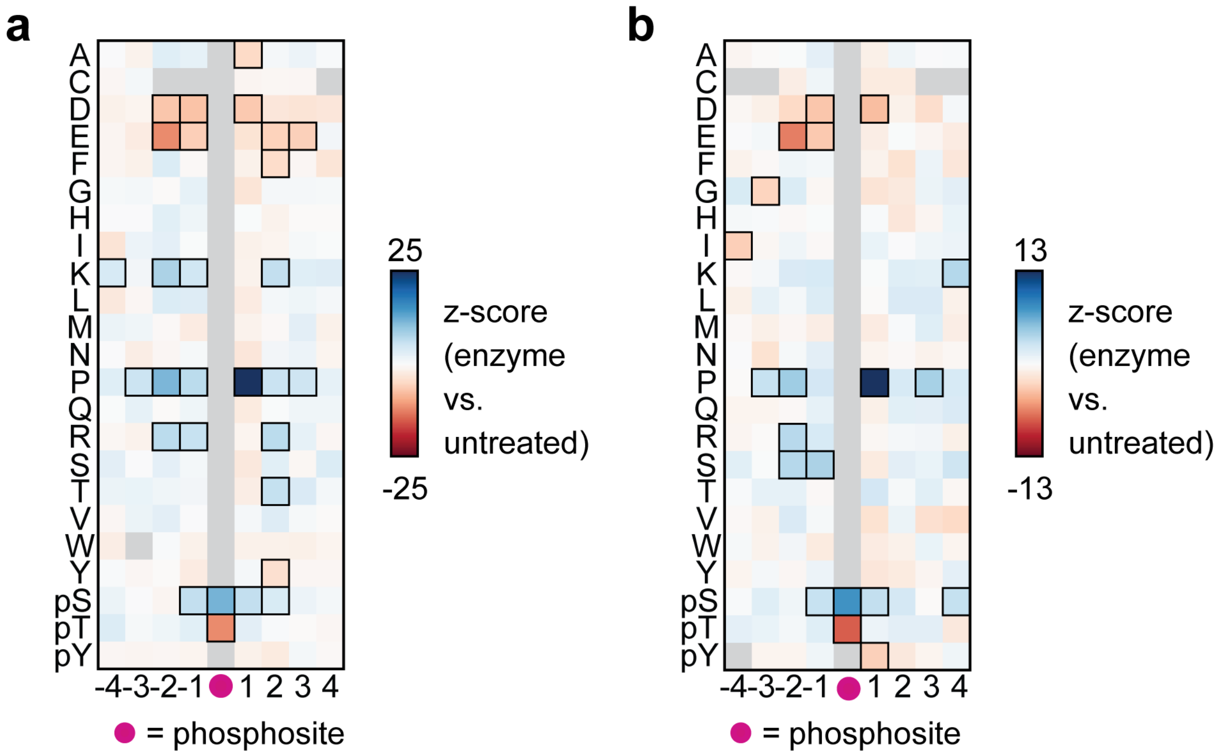

**Figure S44. Comparison of $OspF_{27-239}$ specificity profiling experiments performed with PhosPropels generated with different proteases.** All experiments used PhosPropels generated from pervanadate-treated HEK293T cells. Dotted lines indicate $z = \pm 4$ (corresponding to $\alpha \approx 0.0001$). Points are colored according to residue identity (see legend). Points with $|z| \geq 4$ of the same sign (upper right and lower left regions of the plot) represent residue-position combinations that were enriched or depleted consistently in both libraries. Points near the origin represent residue-position combinations with little or no enrichment or depletion in either dataset, whereas points exceeding the threshold in only one dataset indicate features enriched or depleted in one library but not the other. Residue-position combinations with large, opposing z-scores ($|z| \geq 4$ in both datasets but with opposite sign) appear in the upper left and lower right regions of the plot and indicate protease-dependent differences in inferred specificity. These features are circled in red and labeled. a) Scatterplot comparing z scores obtained using trypsin PhosPropels and LysC PhosPropels. b) Scatterplot comparing z scores obtained using trypsin PhosPropels and GluC PhosPropels. c) Scatterplot comparing z scores obtained using GluC PhosPropels and LysC PhosPropels.

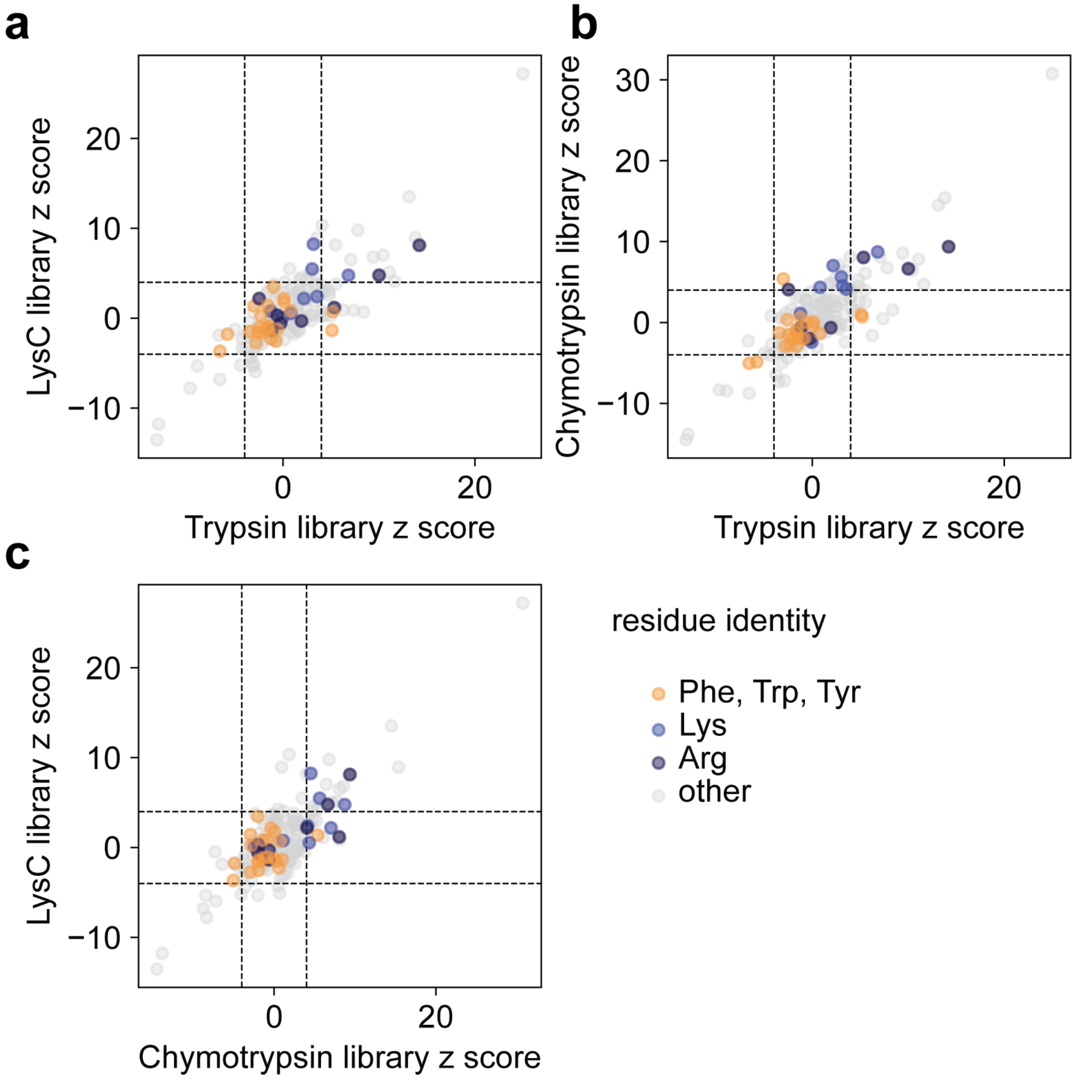

**Figure S45. Western blot analysis of MAPK activation loop phosphorylation state.** Experiments were performed using HEK293T cells expressing either wt OspF or the catalytic dead variant OspF-K134A under a doxycycline-inducible promoter. a) Cells were treated with epidermal growth factor (EGF) to induce Erk1/2 and p38 phosphorylation in the presence (+) or absence (-) of doxycycline to induce OspF expression. OspF expression was monitored via its V5 epitope tag, and β-tubulin served as a loading control. b) Cells were treated with anisomycin to induce phosphorylation of JNK in the presence (+) or absence (-) of doxycycline to induce OspF expression. OspF expression was monitored via its V5 epitope tag, and β-tubulin or α-actinin served as a loading control.

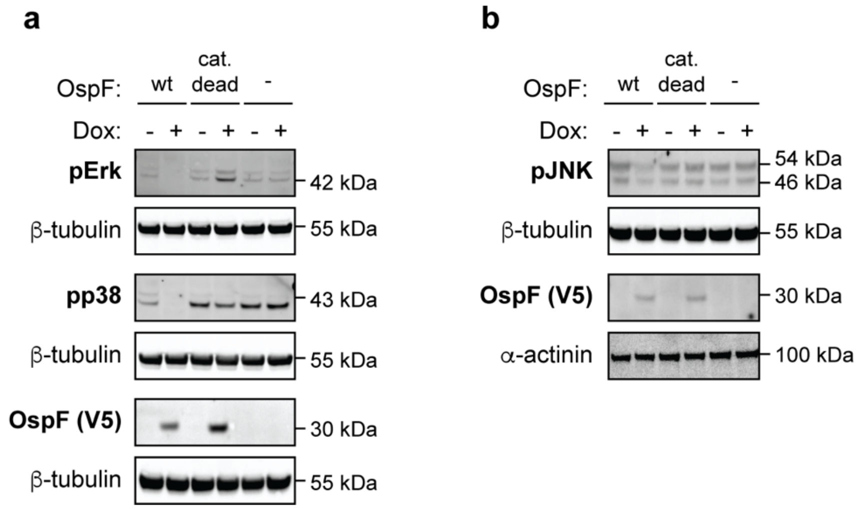

**Figure S46. Label-free quantification of the phosphoproteome in EGF-treated HEK293T cells expressing OspF under a doxycycline-inducible promoter.** The phosphoproteome was quantified in the presence or absence of OspF expression.

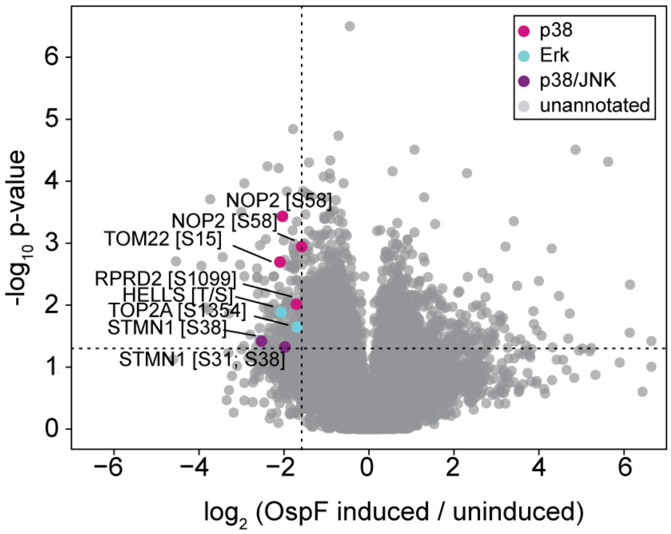

**Figure S47. Specificity profiles of OspF alanine variants compared to wild-type OspF.** Z-scores were calculated by comparing positional frequencies flanking β-eliminated sites in OspF variant-treated samples and wild-type OspF-treated samples. Counts were summed across n = 3 biological replicates. Residue-position combinations with Benjamini-Hochberg FDR-adjusted p-values < 0.0001 were considered significant and are outlined in black.

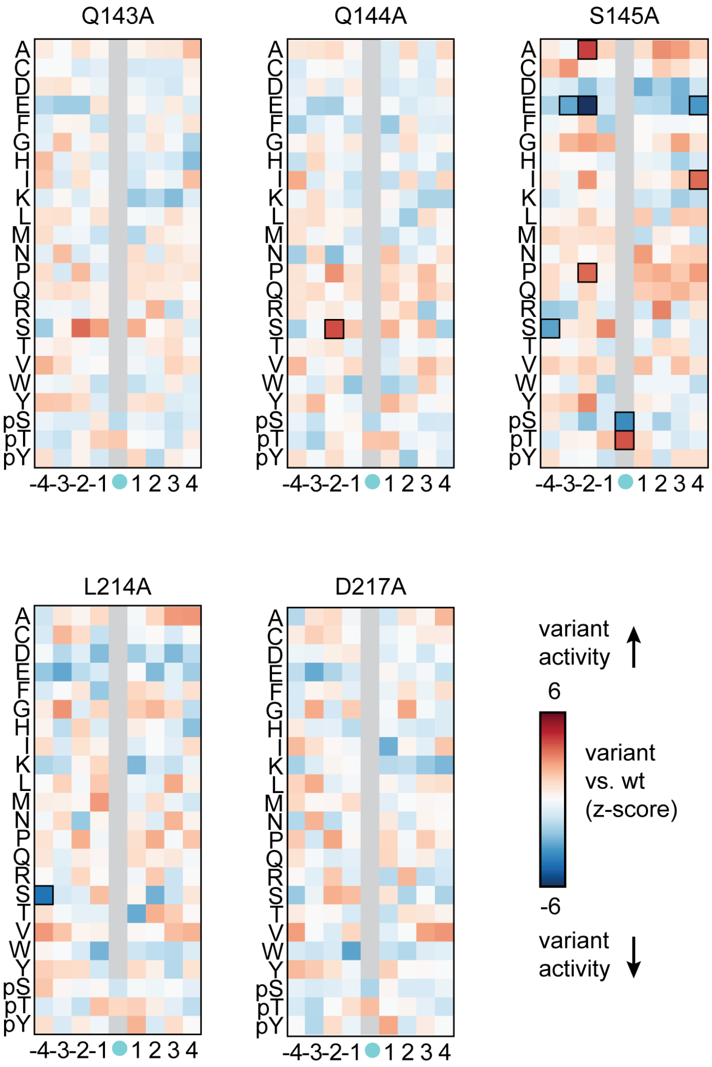

**Figure S48. Structural rendering of SpvC highlighting residues in OspF targeted for alanine scanning mutagenesis.** Numbering corresponds to OspF numbering. Residues discussed in the main text are show in pale cyan and residues not discussed in the main text are shown in pink (Q143A, Q144A, S145A, L214A, D217A). These residues had only modest effects on OspF substrate selectivity (see Fig. S36) and generally do not make contacts with the substrate. Figure was generated using PDB ID 2Q8Y.

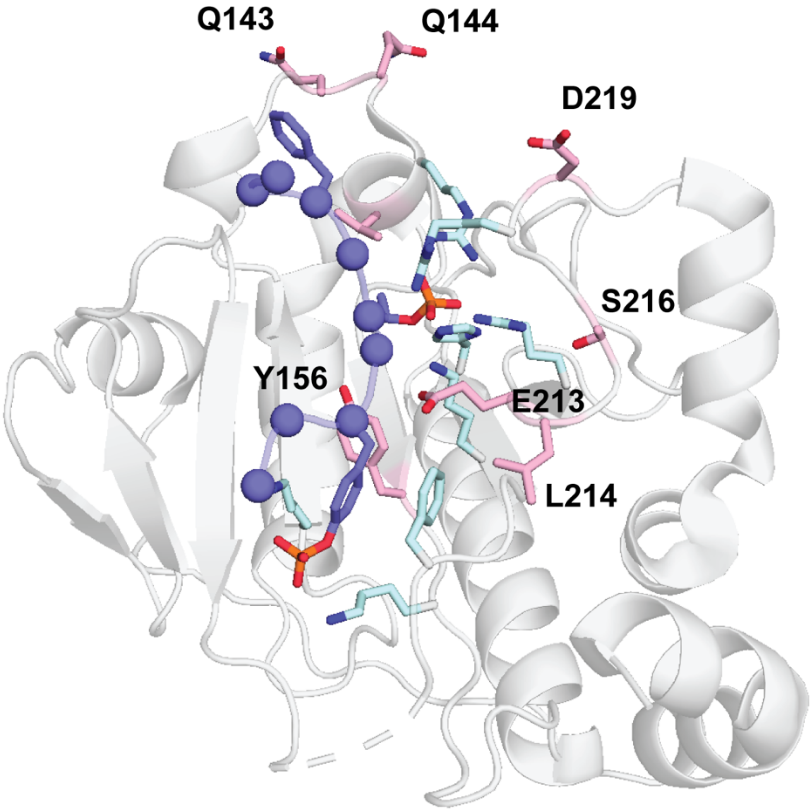

**Figure S49. Characterization of WipA and WipB.** a) SDS-PAGE analysis of purified WipA (lane 2) and WipB (lane 3). b) LC-TOF MS analysis of WipA. The major species has a mass that is -15 Da relative to the expected mass (*). We attribute this to an unexpected modification (e.g., methionine loss + carbamylation) or lower accuracy of the TOF in the higher mass range. c) LC-TOF MS analysis of WipB.

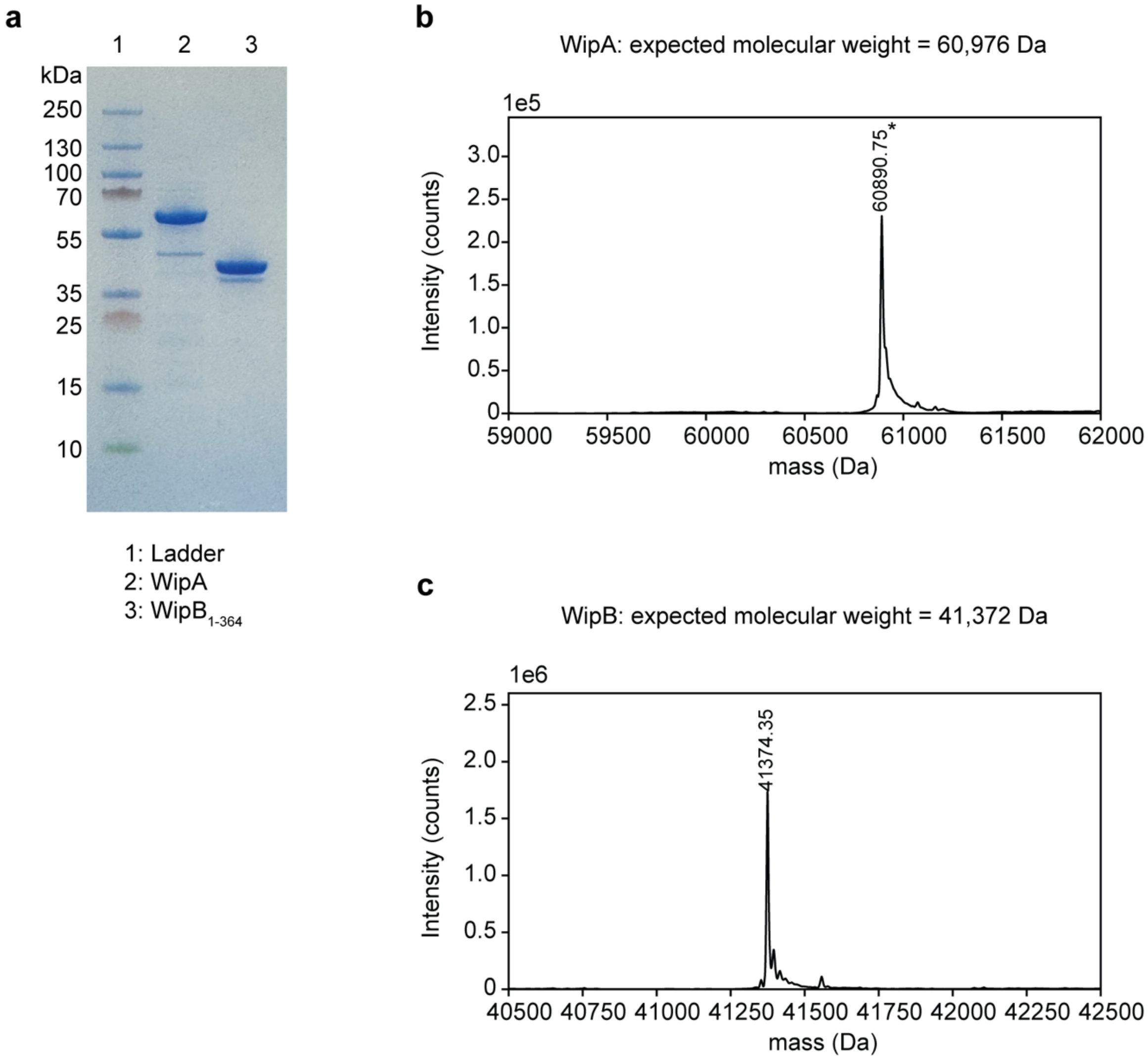

**Figure S50. Characterization of OspF, SpvC, and HopAI.** a) SDS-PAGE analysis of purified OspF (lane 2). b) LC-TOF MS analysis of OspF. c) SDS-PAGE analysis of purified SpvC (lane 2). d) LC-TOF MS analysis of SpvC. e) SDS-PAGE analysis of purified HopAI (lane 2). f) LC-TOF MS analysis of HopAI.

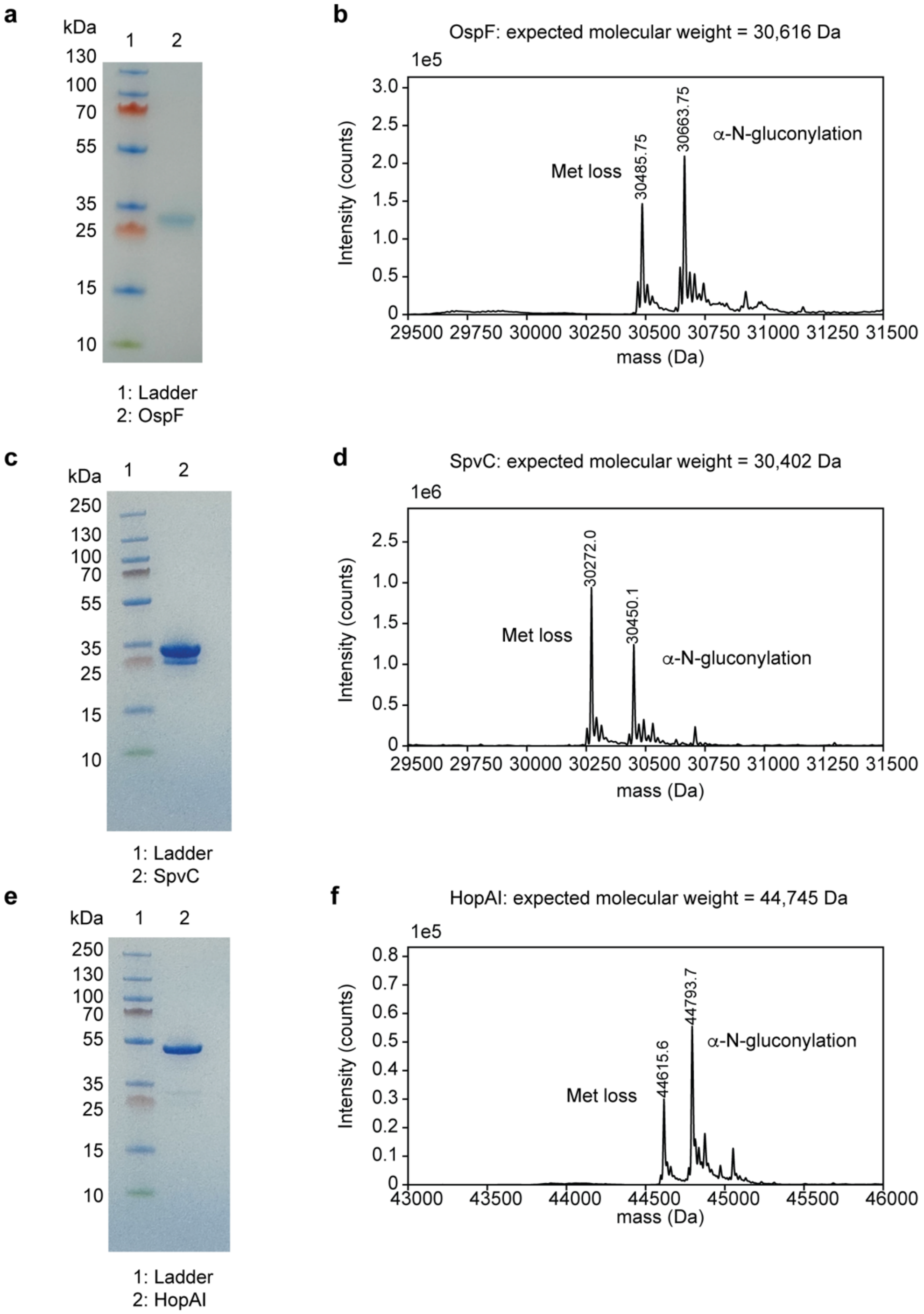

**Figure S51. Frequency heatmaps showing the sequence context of missed cleavage sites in trypsin, GluC, and LysC libraries.** Heatmaps show the frequency of each amino acid in each position flanking missed cleavage sites. Positions are label P5-P4' according to Schecter and Berget nomenclature, with the scissile bond of the missed cleavage site occurring between P1 and P1'. a) Missed cleavages in trypsin libraries. b) Missed cleavages in GluC libraries. c) Missed cleavages in LysC libraries.

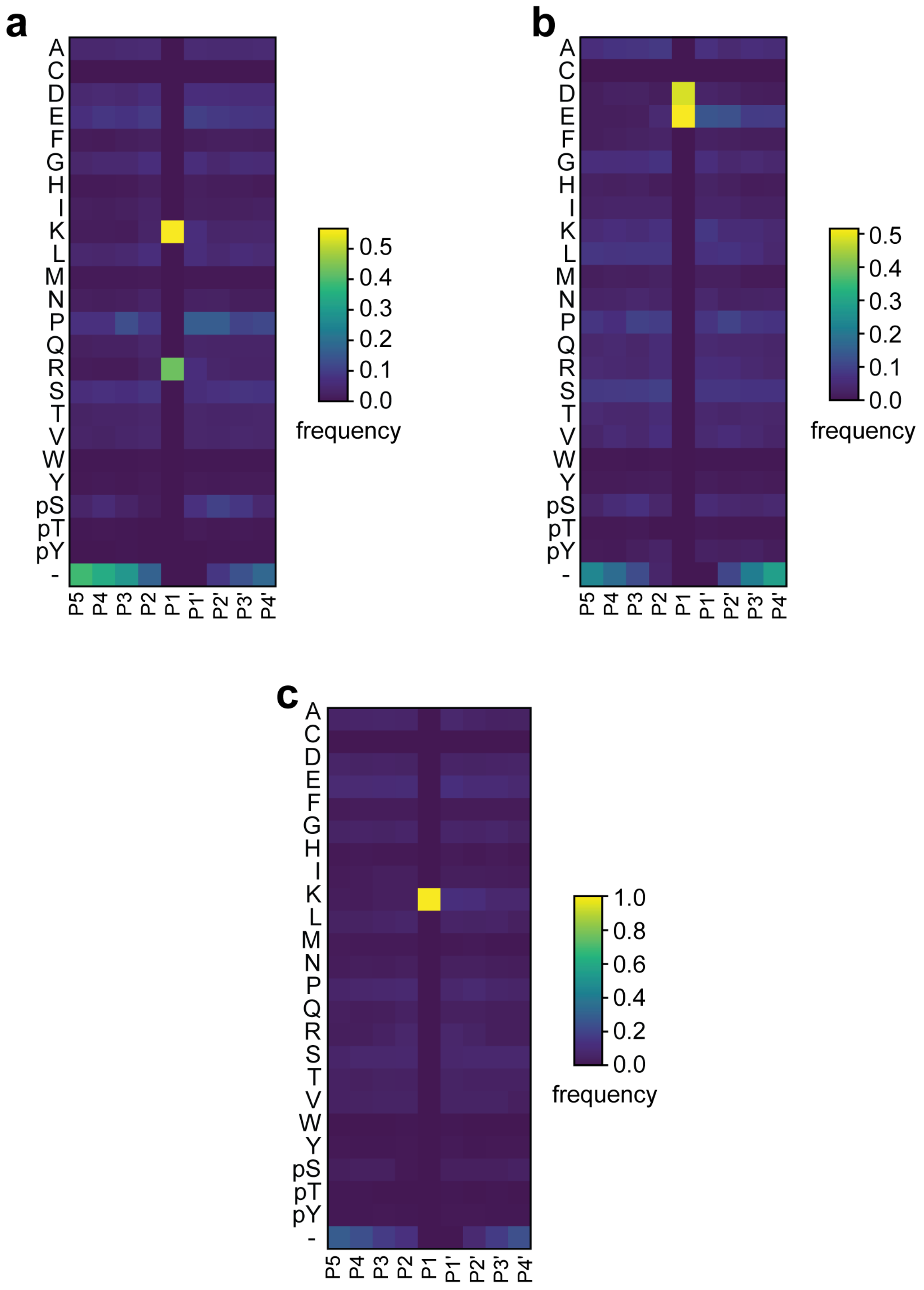

**Supplementary Appendix: Representative spectra for peptides containing dehydroalanine (Dha) and dehydrobutyrine (Dhb) sites.**

**Annotated spectrum for LADFGVAGQL-Dhb-DTQIK.** (A) Spectrum for monoisotopic m/z = 829.94029, z = 2. Matched y-ions are shown in blue and matched b-ions are shown in red. Ammonia loss peaks are marked with a * and water loss peaks are marked with a •. Ions $y_4$, $y_6$, $y_7$, $y_8$, $y_9$, $y_{10}$, $y_{11}$, $y_{12}$, and $y_{13}$ (bolded) are shifted by -18.0 Da relative to the theoretical m/z for the unmodified peptide, supporting localization of the modification at peptide position 13 (ptmRS site probability = 100%). (B) Theoretical m/z for y-ion and b-ion series of unmodified LADFGVAGQLTDTQIK and LADFGVAGQLTDTQIK in which Thr 13 has been converted to Dhb. Matched y-ions are colored in blue and matched b-ions are colored in red. Fragments with shifted m/z between the modified and unmodified peptides are bolded.

A

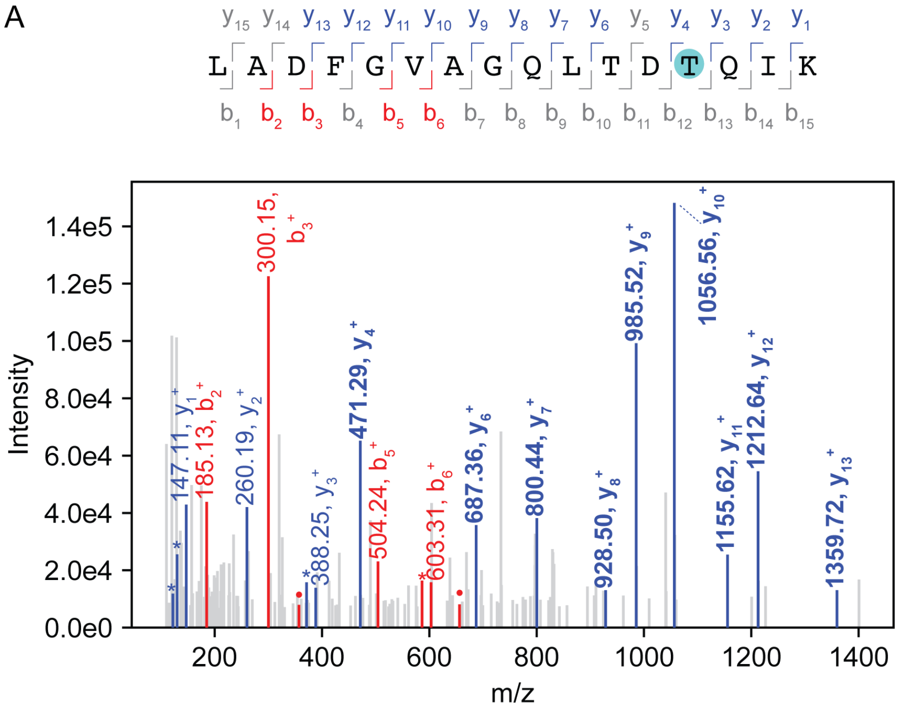


B

| b⁺, unmodified | b⁺, modified | b-ion # | | y-ion # | y⁺, unmodified | y⁺, modified |
|---|---|---|---|---|---|---|
| 114.09134 | 114.09134 | 1 | L | 16 | | |
| 185.128454 | 185.128454 | 2 | A | 15 | 1563.801313 | 1545.790748 |
| 300.155397 | 300.155397 | 3 | D | 14 | 1492.7642 | 1474.753635 |
| 447.223811 | 447.223811 | 4 | F | 13 | 1377.737257 | **1359.726692** |
| 504.245275 | 504.245275 | 5 | G | 12 | 1230.668843 | **1212.658278** |
| 603.313689 | 603.313689 | 6 | V | 11 | 1173.647379 | **1155.636814** |
| 674.350803 | 674.350803 | 7 | A | 10 | 1074.578965 | **1056.5684** |
| 731.372267 | 731.372267 | 8 | G | 9 | 1003.541851 | **985.531286** |
| 859.430844 | 859.430844 | 9 | Q | 8 | 946.520387 | **928.509822** |
| 972.514908 | 972.514908 | 10 | L | 7 | 818.46181 | **800.451245** |
| 1073.562587 | 1073.562587 | 11 | T | 6 | 705.377746 | **687.367181** |
| 1188.58953 | 1188.58953 | 12 | D | 5 | 604.330067 | 586.319502 |
| 1289.637208 | 1271.626643 | 13 | T | 4 | 489.303124 | **471.292559** |
| 1417.695786 | 1399.685221 | 14 | Q | 3 | 388.255446 | 388.255446 |
| 1530.77985 | 1512.769285 | 15 | I | 2 | 260.196868 | 260.196868 |
| | | 16 | K | 1 | 147.112804 | 147.112804 |

**Annotated spectrum for TPEELDD-Dha-DFETEDFDVR.** (A) Spectrum for monoisotopic m/z = 1070.95561, z = 2. Matched y-ions are shown in blue and matched b-ions are shown in red. Ammonia loss peaks are marked with a * and water loss peaks are marked with a •. Ions $y_{11}$-$y_{16}$, and $b_8$, $b_9$, and $b_{10}$ (bolded) are shifted by -18.0 Da relative to the theoretical m/z for the unmodified peptide, supporting localization of the modification at peptide position 8 (ptmRS site probability = 100%). (B) Theoretical m/z for y-ion and b-ion series of unmodified TPEELDDSDFETEDFDVR and TPEELDDSDFETEDFDVR in which Ser 8 has been converted to Dha. Matched y-ions are colored in blue and matched b-ions are colored in red. Fragments with shifted m/z between the modified and unmodified peptides are bolded.

A

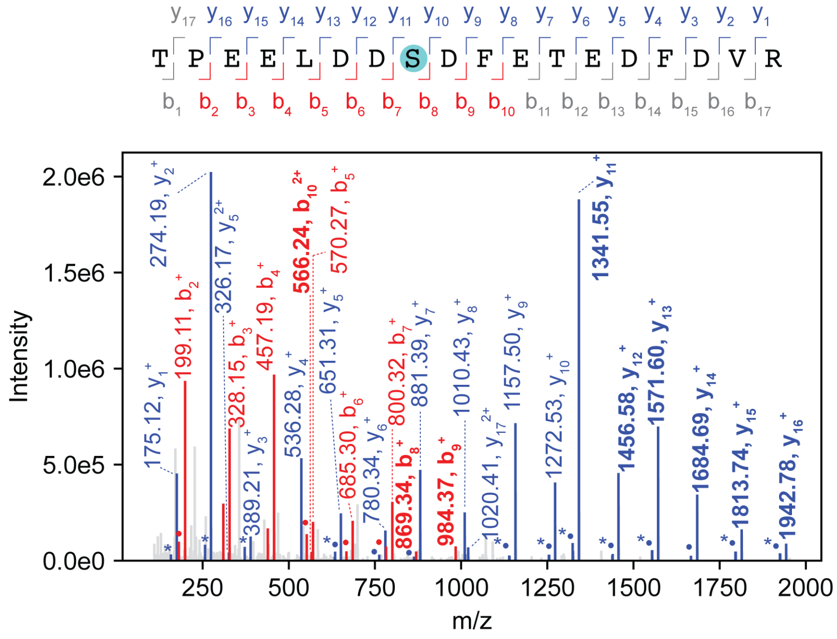


B

| b⁺, unmodified | b⁺, modified | b-ion # | | y-ion # | y⁺, unmodified | y⁺, modified |
|---|---|---|---|---|---|---|
| 102.055 | 102.055 | 1 | T | 18 | | |
| 199.1077 | 199.1077 | 2 | P | 17 | 2057.846 | 2039.835 |
| 328.1503 | 328.1503 | 3 | E | 16 | 1960.793 | **1942.782** |
| 457.1929 | 457.1929 | 4 | E | 15 | 1831.75 | **1813.74** |
| 570.277 | 570.277 | 5 | L | 14 | 1702.708 | **1684.697** |
| 685.3039 | 685.3039 | 6 | D | 13 | 1589.624 | **1571.613** |
| 800.3309 | 800.3309 | 7 | D | 12 | 1474.597 | **1456.586** |
| **887.3629** | 869.3523 | 8 | S | 11 | 1359.57 | **1341.559** |
| **1002.39** | 984.3793 | 9 | D | 10 | 1272.538 | 1272.538 |
| **1149.458** | 1131.448 | 10 | F | 9 | 1157.511 | 1157.511 |
| 1278.501 | 1260.49 | 11 | E | 8 | 1010.443 | 1010.443 |
| 1379.549 | 1361.538 | 12 | T | 7 | 881.3999 | 881.3999 |
| 1508.591 | 1490.581 | 13 | E | 6 | 780.3523 | 780.3523 |
| 1623.618 | 1605.607 | 14 | D | 5 | 651.3097 | 651.3097 |
| 1770.686 | 1752.676 | 15 | F | 4 | 536.2827 | 536.2827 |
| 1885.713 | 1867.703 | 16 | D | 3 | 389.2143 | 389.2143 |
| 1984.782 | 1966.771 | 17 | V | 2 | 274.1874 | 274.1874 |
| | | 18 | R | 1 | 175.119 | 175.119 |

**Annotated spectrum for HTDDEM-Dhb-G-pTyr-VATR.** (A) Spectrum for monoisotopic m/z = 779.30698, z = 2. Matched y-ions are shown in blue and matched b-ions are shown in red. Ammonia loss peaks are marked with a * and water loss peaks are marked with a •. Ions $y_5$ and $y_6$ are shifted by 80 Da relative to the theoretical m/z for the unmodified peptide, supporting localization of the phospho modification at peptide position 9 (ptmRS site probability = 99.5%). Ions $b_7$ and $b_8$ are shifted by -18 Da relative to the theoretical m/z for the unmodified peptide, supporting localization of the dehydration modification at position 7. Ions $y_7$-$y_{12}$ and $b_9$ are shifted by 62 Da (80 Da for phospho at position 9 and -18 Da for dehydration on position 7) relative to the theoretical m/z values for the unmodified peptide, supporting localization the dehydration modification at peptide position 7 (ptmRS site probability = 100%). (B) Theoretical m/z for y-ion and b-ion series of HTDDEM-Dhb-G-pTyr-VATR. Matched y-ions are colored in blue and matched b-ions are colored in red. Ions in bold have the dehydration modification, ions in italic have the phosphor modification, and ions in bold italic have both modifications.

A

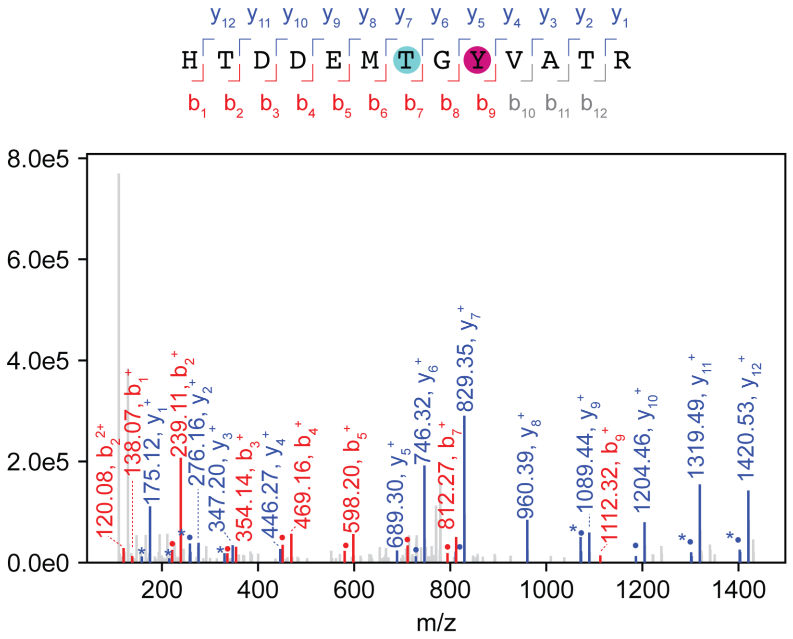


B

| b⁺ | b-ion # | | y-ion # | y⁺ |
|---|---|---|---|---|
| 138.0662 | 1 | H | 13 | |
| 239.1139 | 2 | T | 12 | ***1420.545*** |
| 354.1408 | 3 | D | 11 | ***1319.497*** |
| 469.1678 | 4 | D | 10 | ***1204.47*** |
| 598.2103 | 5 | E | 9 | ***1089.443*** |
| 729.2508 | 6 | M | 8 | ***960.4009*** |
| **812.2879** | 7 | T | 7 | ***829.3604*** |
| **869.3094** | 8 | G | 6 | *746.3233* |
| ***1112.339*** | 9 | Y | 5 | *689.3018* |
| 1211.407 | 10 | V | 4 | 446.2722 |
| 1282.445 | 11 | A | 3 | 347.2037 |
| 1383.492 | 12 | T | 2 | 276.1666 |
| | 13 | R | 1 | 175.119 |

**Supplementary Note: Statistical framework for phosphoeraser specificity profiling using PhosPropels.**

The PhosPropel approach enables statistical comparison of amino acid frequencies at positions surrounding a central modified site in an enzyme-treated sample versus an appropriate control. Here, we describe general considerations that make this comparison possible and the specific analyses that were performed in our manuscript.

**Background set**
Rather than relying on a theoretical or computationally derived background set, we used an experimentally measured control as the background for all statistical comparisons. This approach ensures that enrichment or depletion is assessed relative to the actual position-specific distribution of amino acids present in the phosphoproteome-derived peptide library. The experimental background set accounts for position-specific biases in amino acid composition surrounding phosphosites (e.g., Pro occurs much more frequently in the position following pSer than its overall frequency in the proteome) and for experimental treatments that alter phosphosite composition in the library (e.g. treatment with pervanadate or other phosphatase inhibitors).

**Sample set**
Sample sets are generated by treating the phosphoproteome-derived peptide library with a phosphoeraser enzyme of interest. To profile phosphatase specificity, the remaining phosphosites in the library can be analyzed and the position-specific frequencies of each amino acid in each position surrounding the phosphosites can be calculated. Good substrates contain features that are depleted from the library, while poor substrates contain features that persist in library after enzyme treatment. To profile phospholyase specificity, the appearance of β-eliminated Ser and Thr residues (dSer and dThr) can be analyzed and the position-specific frequency of each amino acid in each position surrounding the β-eliminated site can be calculated. Good substrates contain features that are enriched in positions surrounding the β-eliminated sites, while poor substrates contain features that appear less frequently surrounding β-eliminated sites.

**Statistical comparison to identify sequence features that influence phosphoeraser substrate preference**
In comparing the background set and the sample set, the null hypothesis is that the frequency of amino acid X at position i is the sample in the treated sample versus background sample, and that all amino acids at position i are equally likely to be found among substrates of the enzyme under study. The alternative hypothesis is that the frequency of amino acid X at position i is different in the treated sample versus background sample, reflecting enzyme-specific substrate preferences.

To test these hypotheses, we calculated z-scores for each amino acid-position pair according to the following formula:

$$z = \frac{p_{sample} - p_{control}}{SE}$$

where $p_{sample}$ is the observed frequency of amino acid X at position i in the enzyme-treated sample, $p_{control}$ is the observed frequency of amino acid X at position i in the background sample, and SE is calculated according to the formula:

$$SE = \sqrt{\frac{p_{sample}(1 - p_{sample})}{n_{sample}} + \frac{p_{control}(1 - p_{control})}{n_{control}}}.$$

We treated the frequency of each amino acid at each position as a binomial proportion, assuming that each peptide provides an independent observation of residue identity at a given position. Under this assumption, the variance of each frequency estimate can be calculated based on the binominal distribution, and differences in frequency can be standardized using the combined standard error. We applied the normal approximation to the binomial distribution for z-score calculation based on the central limit theorem. This approximation is appropriate given the large number of measurements in both conditions and the sufficient count of most amino acid-position combinations.

The z-score reflects how many standard errors the observed difference is from the null expectation (i.e., the background frequency). The z-score approach accounts for not only the difference in observed frequencies, but also for variability associated with the number of observations of each amino acid in each position due to uneven amino acid usage in the proteome and due to experimental considerations. Because z-scores incorporate the number of observations for each amino acid at each position, they effectively scale the confidence of each comparison. For example, a large difference in frequency between the treated sample and the untreated control that is supported by a large number of observations produces a higher-magnitude z-score, while differences based on only a few measurements produce z-scores of lower magnitudes.

We note that our statistical approach is very similar to that used to produce IceLogos with small changes in how SE is calculated based on our experimental design.

**Analysis of position-specific frequencies in PhosPropels compared to the proteome**
For analysis of the phosphosite-flanking residues in PhosPropels, we chose as our sample set all phosphosites confidently identified and localized using LC/MS analysis. We aligned these phosphosites and counted the frequency of each amino acid at each position four residues N-terminal (- side) and four residues C-terminal (+ side) to the phosphosite. As a control/background sample, we measured the overall frequency of each of the amino acid in the sample in a manner that was not position-specific. We favored this approach as opposed to using previously reported information about the abundance of each amino acid in the human as it allowed us to measure the frequency of occurrence of pSer, pThr, and pTyr in the sample, enabling us to ask whether these amino acids were enriched or depleted relative to other phosphosites.

**PhosPropel-based profiling of phosphatase specificity**
For analysis of phosphatase specificity using PhosPropels, we chose as our sample set all phosphosites confidently identified and localized using LC-MS/MS analysis. We chose as our control/background set all phosphosites confidently identified and localized using LC-MS/MS in an untreated or 0 min timepoint sample. To compare the distributions of phosphosite features, we aligned the phosphosites in both sample and control, computed the position-specific frequencies of each amino acid, and calculated z-scores as described above to compare them. We reasoned that features associated with good phosphatase substrates would be specifically depleted from the library upon phosphatase treatment, while features associated with poor substrates would become enriched in the library upon phosphatase treatment.

**PhosPropel-based profiling of phospholyase specificity**
For analysis of phospholyase specificity using PhosPropels, we chose as our sample set all β-eliminated sites that were confidently identified and localized using LC-MS/MS analysis. These sites could be identified based on a -18.01 Da mass shift relative to the unmodified amino acid. We chose as our control/background set all phosphosites confidently identified and localized using LC-MS/MS in the same sample. We aligned the β-eliminated sites in the sample and the phosphosites in the control, computed the position-specific frequencies of each amino acid, and calculated z-scores as described above to compare them. We reasoned that features associated with good phospholyase substrates would be specifically enriched among β-eliminated sites, while features associated with poor substrates would become enriched among phosphosites.

**Comparison of wild-type OspF vs. variant OspF specificity**
For comparison of the sequence specificity of wild-type OspF and OspF variants, we chose as our sample set all β-eliminated sites that were confidently identified and localized using LC-MS/MS analysis in the variant-treated sample. We chose as our control/background set all β-eliminated sites confidently identified and localized using LC-MS/MS in a wild-type OspF-treated sample. We aligned the β-eliminated sites in the sample and in the control, computed the position-specific frequencies of each amino acid, and calculated z-scores as described above to compare them. Features associated with improved variant activity compared to wild-type were specifically enriched in among β-eliminated sites in the variant-treated sample, while features associated with poor variant substrates substrates were specifically enriched among β-eliminated sites in the wild-type OspF-treated control.

**Estimating statistical power for specific residue-position combinations**
Library composition is influenced not only by protease cleavage specificity, but also by the proteome abundance of amino acids and the sequences preferences of kinases that install phosphosites. Because enrichment and depletion are evaluated relative to matched input libraries, these baseline compositional biases are internally controlled and do not influence inferences about phosphatase/phospholyase specificity. However, they do influence statistical sensitivity. We therefore examined how residue representation and sampling depth influence the minimum detectable effect size at a given residue-position combination. Because enrichment and depletion are evaluated relative to matched input libraries, assay sensitivity depends on both baseline residue frequency and the number of observations in the treated condition. Under the null hypothesis, the expected number of observations ($E_i$) of a given residue r at position *i* in the enzyme-treated sample is given by

$$E_i = p_{0,i} N_{treated}$$

where $p_{0,i}$ is the frequency of residue r at position *i* in the input library and $N_{treated}$ is the number of observations of position i under the enzyme-treated condition. We can approximate the minimum detectable fold-change at a two-sided significance level of α and power 1 - β as

$$FC_{min,i} = 1 + \frac{z_{1-\alpha/2} + z_{1-\beta}}{\sqrt{E_i}}$$

using the normal approximation to the binomial distribution (see Chow et al, *Sample Size Calculations in Clinical Research*, Taylor and Francis, 2017). Residues with larger expected counts $E_i$ allow for detection of smaller fold-changes, whereas sparse residue-position combinations require larger effect sizes to achieve statistical significance.

As an example, we used the above formula to assess the minimum detectable fold-change for Lys and Arg across positions flanking the phosphosite at 80% power and significance levels of α = 0.0001, 0.001, 0.01, and 0.05 for the lowest number of observations that we made across the panel of enzymes that we tested ($N_{treated}$ ~ 1000 sites/per position for OspF). Under these conditions, the minimum detectable fold-change at a very stringent α of 0.0001 is generally less than twofold, except at the +1 position, where the prevalence of proline leads to lower representation of other amino acids and the minimum detectable fold-change is approximately 2.2-fold at α = 0.0001 (see figure below). We note that for most of the enzymes tested, $N_{treated}$ was much higher for at least one time point, enabling robust detection of amino acid preferences.

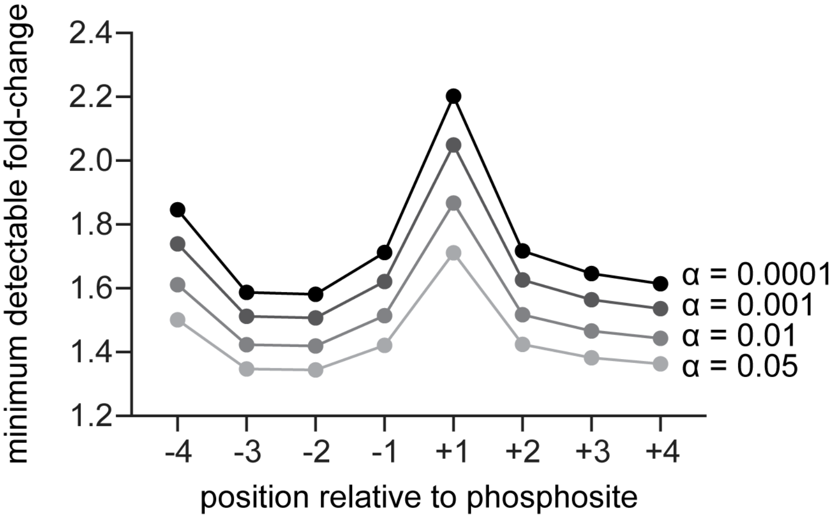


***Plot of minimum detectable fold-change as a function of phosphosite position at varying levels of statistical significance (α).*** *Calulations were perfomed assuming 80% statistical power and $N_{treated}$ = 1000 observations per position.*

**Evaluating and enhancing assay sensitivity**

The above-described fold-change calculation can be applied to any residue-position combination of interest to evaluate the sensitivity of the PhosPropel assay for assessing enzyme selectivity for that combination. Assay sensitivity can be increasing $N_{treated}$. This can be achieved by performing additional replicates or producing libraries with better representation of the residue-position combination of interest.

## SI References


1. Jersie-Christensen, R. R., Sultan, A. & Olsen, J. V. Simple and reproducible sample preparation for single-shot phosphoproteomics with high sensitivity. *Methods Mol. Biol.* **1355**, 251–260 (2016).
2. Rappsilber, J., Mann, M. & Ishihama, Y. Protocol for micro-purification, enrichment, pre-fractionation and storage of peptides for proteomics using StageTips. *Nat. Protoc.* **2**, 1896–1906 (2007).
3. Frazier, C. L., Deb, D., Leiter, W. E., Mondal, U. & Weeks, A. M. Engineered reactivity of a bacterial E1-like enzyme enables ATP-driven modification of protein and peptide C termini. *Nat. Chem.* **17**, 1371–1382 (2025).
4. UniProt: the universal protein knowledgebase in 2025. *Nucleic acids research* **53**, D609–D617 (2025).
5. Eng, J. K., McCormack, A. L. & Yates, J. R. An approach to correlate tandem mass spectral data of peptides with amino acid sequences in a protein database. *J. Am. Soc. Mass Spectrom.* **5**, 976–989 (1994).
6. Käll, L., Canterbury, J. D., Weston, J., Noble, W. S. & MacCoss, M. J. Semi-supervised learning for peptide identification from shotgun proteomics datasets. *Nat. Methods* **4**, 923–925 (2007).
7. Taus, T. *et al.* Universal and confident phosphorylation site localization using phosphoRS. *J. Proteome Res.* **10**, 5354–5362 (2011).
8. Johnson, J. L. *et al.* An atlas of substrate specificities for the human serine/threonine kinome. *Nature* **613**, 759–766 (2023).
9. Yaron-Barir, T. M. *et al.* The intrinsic substrate specificity of the human tyrosine kinome. *Nature* **629**, 1174–1181 (2024).
10. Metz, K. S. *et al.* Coral: Clear and customizable visualization of human kinome data. *Cell Syst.* **7**, 347-350.e1 (2018).
11. Abramson, J. *et al.* Accurate structure prediction of biomolecular interactions with AlphaFold 3. *Nature* **630**, 493–500 (2024).
